# Exploring Rigid and Flexible Scaffolds to Develop Potent Glucuronic Acid Glycodendrimers for Dengue Virus Inhibition

**DOI:** 10.1021/acs.bioconjchem.3c00309

**Published:** 2023-11-15

**Authors:** Alejandro Merchán, Pedro Ramírez-López, Carlos Martínez, José Ramón Suárez, Almudena Perona, María J. Hernáiz

**Affiliations:** Departamento de Química en Ciencias Farmacéuticas, Facultad de Farmacia, Universidad Complutense de Madrid, Plz. Ramón y Cajal s/n, Madrid, C.P. 28040, España

## Abstract

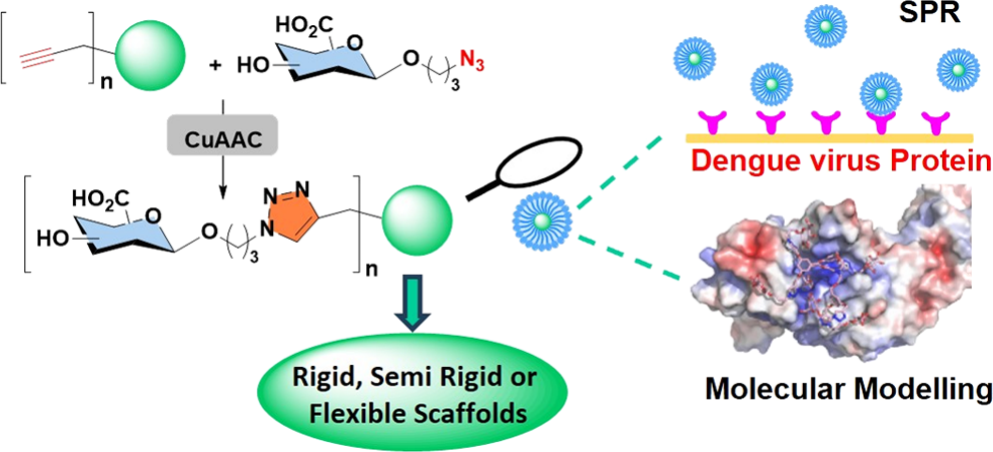

Multivalent glycodendrimers are valuable tools for studying carbohydrate–protein interactions, and their scaffolds represent important components to increase specificity and affinity. Previous work by our group described the preparation of a tetravalent glucuronic acid rigid dendron that binds with good affinity to the dengue virus envelope protein (*K*_D_ = 22 μM). Herein, the chemical synthesis and binding analysis of three new sets of rigid, semirigid, and flexible glucuronic acid-based dendrimers bearing different levels of multivalency and their interactions with the dengue virus envelope protein are described. The different oligoalkynyl scaffolds were coupled to glucuronic acid azides by a copper-catalyzed azide–alkyne cycloaddition reaction through optimized synthetic strategies to afford the desired glycodendrimers with good yields. Surface plasmon resonance studies have demonstrated that glycodendrimers **12b** and **12c**, with flexible scaffolds, give the best binding interactions with the dengue virus envelope protein (**12b**: *K*_D_ = 0.487 μM and **12c**: *K*_D_ = 0.624 μM). Their binding constant values were 45 and 35 times higher than the one obtained in previous studies with a rigid tetravalent glucuronic acid dendron (*K*_D_ = 22 μM), respectively. Molecular modeling studies were carried out in order to understand the difference in behavior observed for **12b** and **12c**. This work reports an efficient glycodendrimer chemical synthesis process that provides an appropriate scaffold that offers an easy and versatile strategy to find new active compounds against the dengue virus.

## Introduction

Heparan sulfate (HS) is a linear and anionic glycosaminoglycan (GAG) composed of repeating units of the disaccharides d-glucuronic acid (GlcA) and N-acetyl-d-glucosamine (GlcNAc) linked together by β-1,4 glycosidic bonds. Usually, it is found attached to a core protein, forming heparan sulfate proteoglycans (HSPGs) that are ubiquitously expressed on the surface of almost all mammalian cells and the extracellular matrix.^1,2^ By interacting with various proteins, HS plays critical roles in numerous pathological and physiological processes, such as embryonic development, inflammation, cancer, cardiovascular diseases, and infectious diseases.^3^ The HSPGs can serve as receptors for different flaviviruses, facilitating specific interactions between hosts and pathogens during the early stage of infection.^4−6^ In particular, the dengue virus (DENV) binds to HS through putative GAG-binding sites within their envelope proteins, with the electrostatic interaction between the negative charge of HS and positive regions on the envelope proteins being the main driving force for binding.^7,8^ The crystal structure of the DENV envelope protein closely resembles those of other flaviviruses, such as West Nile virus, Yellow fever virus, and Zika.^9−11^ Therefore, inhibition of the interaction between the virus envelope protein and HS has been proposed as a promising approach to mitigate these viral infections.^4,10,12^ It is worth mentioning that DENV is the most dangerous flavivirus disease in the world with 100–400 million infections occurring each year, and to date, no specific treatment and effective vaccine have been developed. For this reason, there is an urgent need for the synthesis and development of new antiviral agents against these viral infections.^10,13^

Glycodendrimers (GDs) are an important class of synthetic macromolecules that can be used to mimic many structural and functional features of cell surface glycoconjugates, and they have been increasingly used in recent years as a tool to study carbohydrate–protein interactions, allowing the identification of binding key factors, such as molecular structure, valency, and spatial organization of carbohydrate epitopes in GDs, which are major determinants of specificity and affinity in such interactions.^14−18^

In recent decades, there have been increasing efforts to design and synthesize GDs that could interfere with bacterial and viral infections, suggesting the feasibility of antiadhesive therapy for infectious diseases and the potential of multivalent carbohydrates in this context.^14,19−21^ The number of publications has significantly increased, and many potent antiadhesive glycoclusters, including glycodendrons and GDs, have been proposed. Our research group was the first to report an efficient^12^ and sustainable^22^ copper-catalyzed azide–alkyne cycloaddition reaction (CuAAC) for the synthesis of novel GlcA glycodendrons as potential active compounds in the treatment of DENV. Our previous work described the preparation of a tetravalent GlcA dendron that binds with good affinity to the DENV envelope protein (*K*_D_ = 22 μM).^12^ This dendron was composed of the aromatic methyl 3,5-dihydroxybenzoate scaffold, bearing four copies of the GlcA ligand, and was designed and synthesized to mimic the terminal moiety of the natural HS glycan, which is a major binding determinant of the DENV envelope protein.^12^

Despite these promising results, these multivalent systems were far too rigid, and a limited number of repeating units could be attached, being far from the size of the biologically active polysaccharides. To better mimic the biological activities of the natural products, multivalent systems showing different scaffolds and higher valency are required. Here, we have expanded our synthetic strategy for the preparation of new GlcA GDs bearing different levels of multivalency, polarity, and structural features. In addition, surface plasmon resonance (SPR) experiments in conjunction with molecular modeling were used to investigate the interaction of the new GlcA GDs with the DENV envelope protein.

## Results and Discussion

### Design and Synthesis of GlcA-Based Dendrimers

**Scheme 1:**
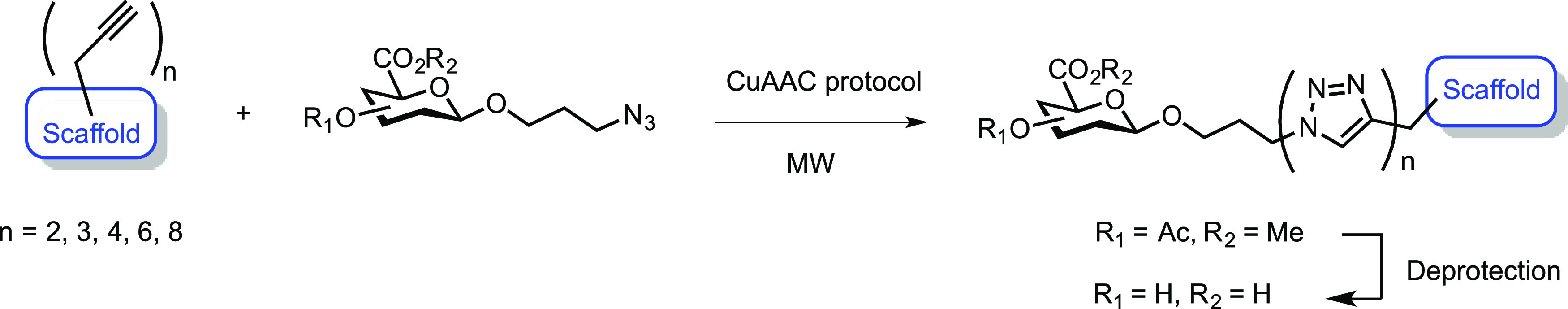
Schematic Synthesis of GlcA-Based Dendrimers

The CuAAC reaction has proven to be extremely useful in the synthesis of GDs due to the comparatively very facile functionalization of organic scaffolds with azides and alkynes, which remain unaffected throughout the subsequent transformations in the presence of highly functionalized biomolecules.^19,23,24^ The CuAAC reaction presents several advantages because this reaction is simple to perform, modular, wide scope, highly efficient, high yielding, and regiospecific, requires readily available alkynes/azides as starting materials and reagents such as copper catalysts, and is conducted in easily removable or benign solvents (e.g., H_2_O or biosolvent).^22,25^ Based on our previous results and with the intention of increasing the affinity of the glycodendrons toward the DENV envelope protein, three new sets of GlcA-based dendrimers bearing different levels of multivalency, polarity, and structural features were designed for affinity binding assays. Their chemical synthesis was performed sequentially in three stages, namely, (a) the chemical synthesis of the oligoalkynyl scaffolds, (b) assembling with fully protected GlcA-based azide units by using a microwave (MW)-assisted CuAAC protocol, and (c) sequential deprotection of hydroxyl and carboxylic acid groups (Scheme 1).

GlcA-derived azide was prepared using a reported procedure^12^ and different scaffolds, as indicated in the Supporting Information.

### Synthesis of Rigid Scaffold-Based GlcA Dendrimers

**Scheme 2:**
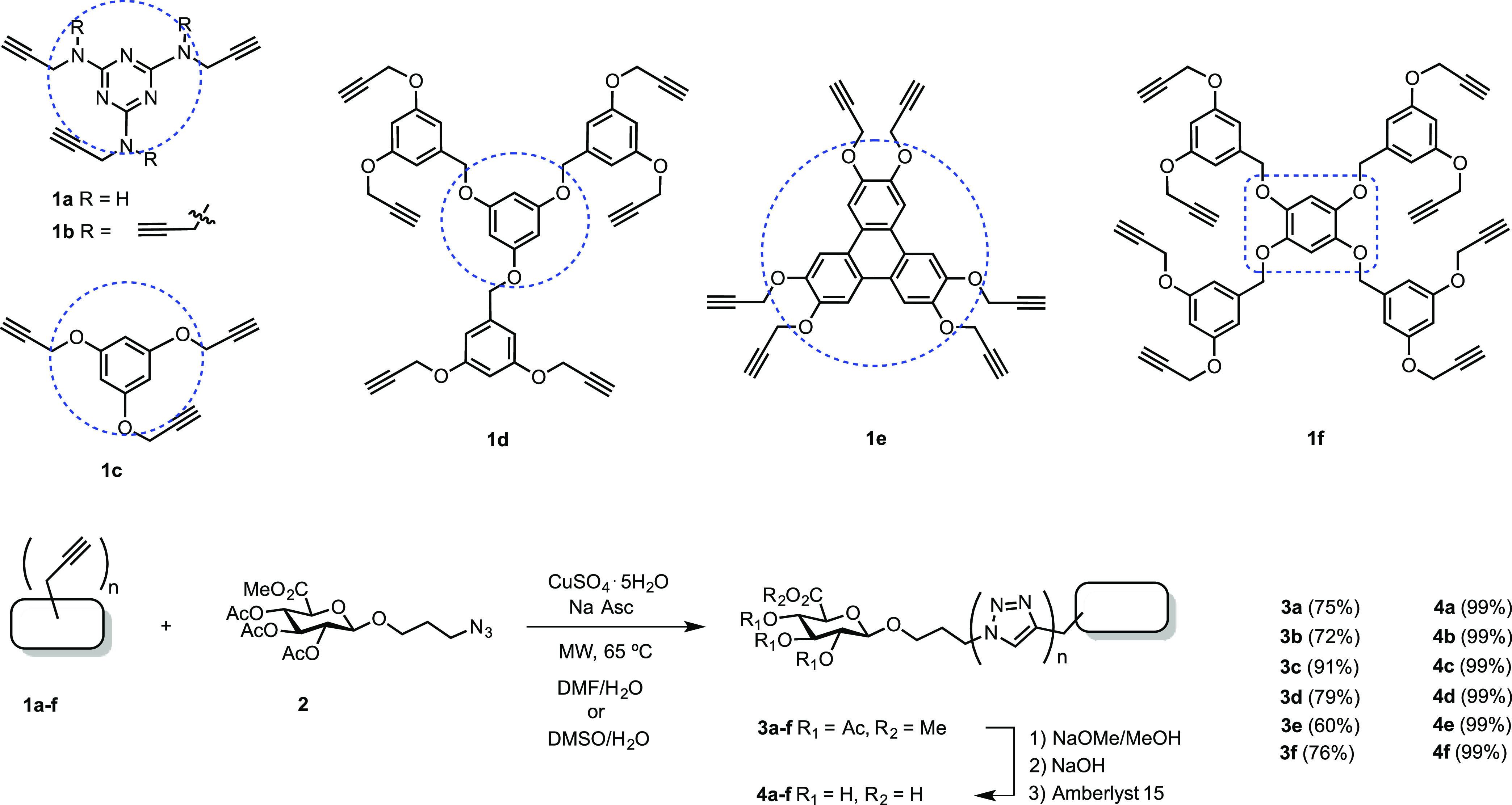
Structures of Rigid Scaffold-Based GlcA Dendrimers

The first topological set was built from starting commercial cyanuric chloride as a heteroaromatic core (compounds **1a** and **1b**) and readily available aromatic scaffolds such as phloroglucinol (compounds **1c**(12) and **1d**), triphenylene-2,3,6,7,10,11-hexaol (compound **1e**), and benzene-1,2,4,5-tetraol (compound **1f**), using classical propargylation strategies (Scheme 2 see the Supporting Information for experimental procedures). These rigid scaffolds will allow us to study the influence of multivalency as well as the rigidity and polarity of the core in the molecular recognition of these structures as a crucial part of multivalent GDs.

The aromatic and heteroaromatic dendritic alkynyl scaffold structures (**1a**–**f**) were assembled with protected GlcA-based azide **2** units by using a microwave (MW)-assisted CuAAC protocol, followed by full deprotection of hydroxyl and carboxylic acid groups, as shown in Scheme 2. GDs **4a**–**f** were obtained in good yields after microwave irradiation at 65 °C until completion of the reaction (90–120 min, depending on the type of scaffold; see details in the Supporting Information) and further deprotection.

### Synthesis of Semirigid Scaffold-Based GlcA Dendrimers

**Scheme 3:**
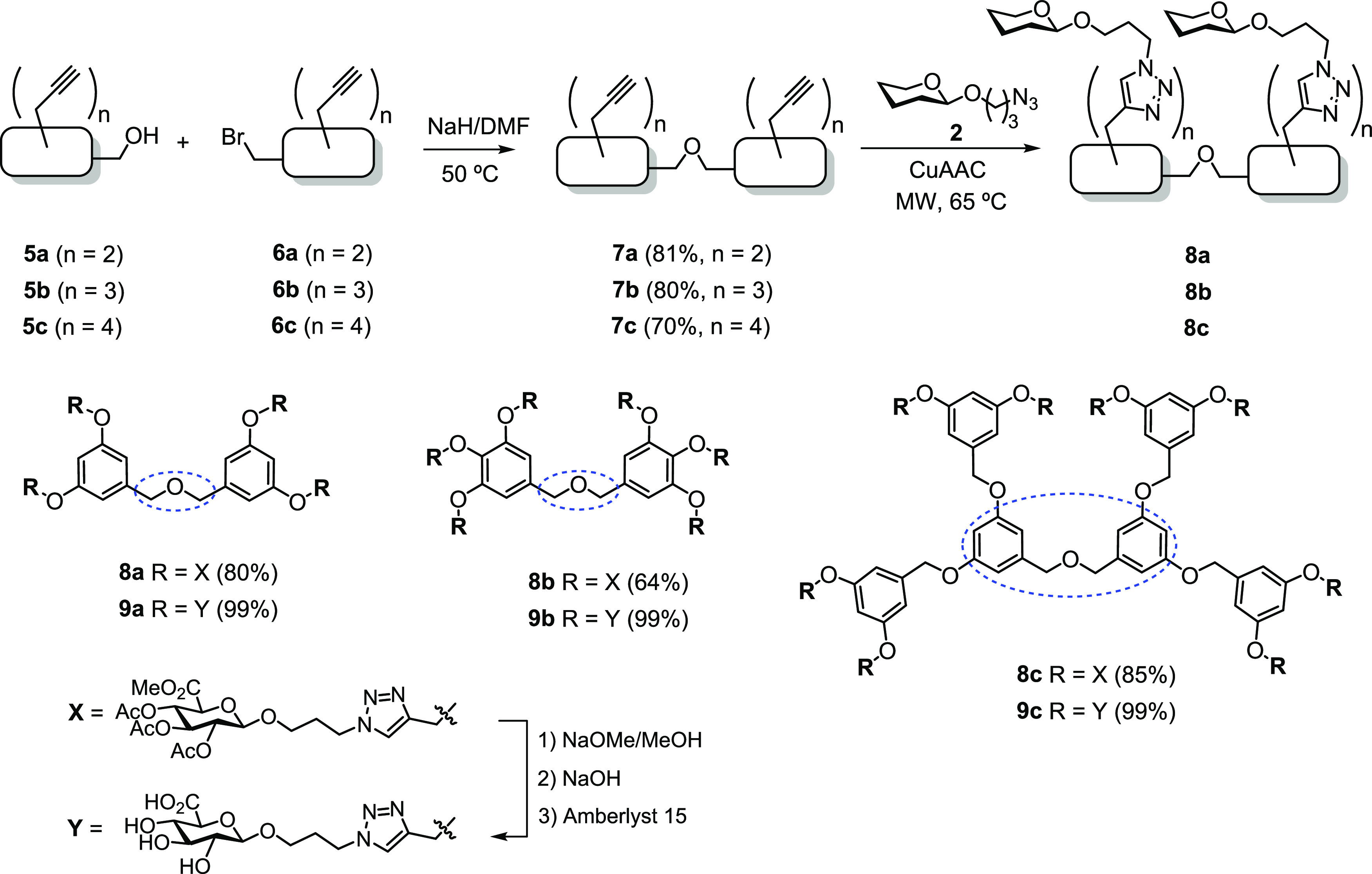
Synthesis of Semirigid Scaffold-Based GlcA Dendrimers

Once the first rigid scaffold-based GD group was prepared, we envisioned a new set of GlcA dendrimers bearing an oxybis(methylene) bridge to increase the flexibility between the aromatic rings in order to facilitate protein binding (Scheme 3). The production of such multivalent GDs started with the preparation of a series of novel C2-symmetric oligoalkynes via Williamson ether synthesis. Deprotonated hydroxyl (alkoxide) compounds **5a**–**c** were reacted with benzyl bromides **6a**–**c** to form corresponding ethers **7a**–**c**, respectively, in good yields. Subsequent coupling of alkynyl scaffolds **7a**–**c** with GlcA-derived azide **2** through CuAAC, followed by full deprotection of hydroxyl and carboxylic acid groups furnished water-soluble GlcA dendrimers **9a**–**c**, respectively (Scheme 3).

### Synthesis of Flexible Scaffold-Based GlcA Dendrimers

**Scheme 4:**
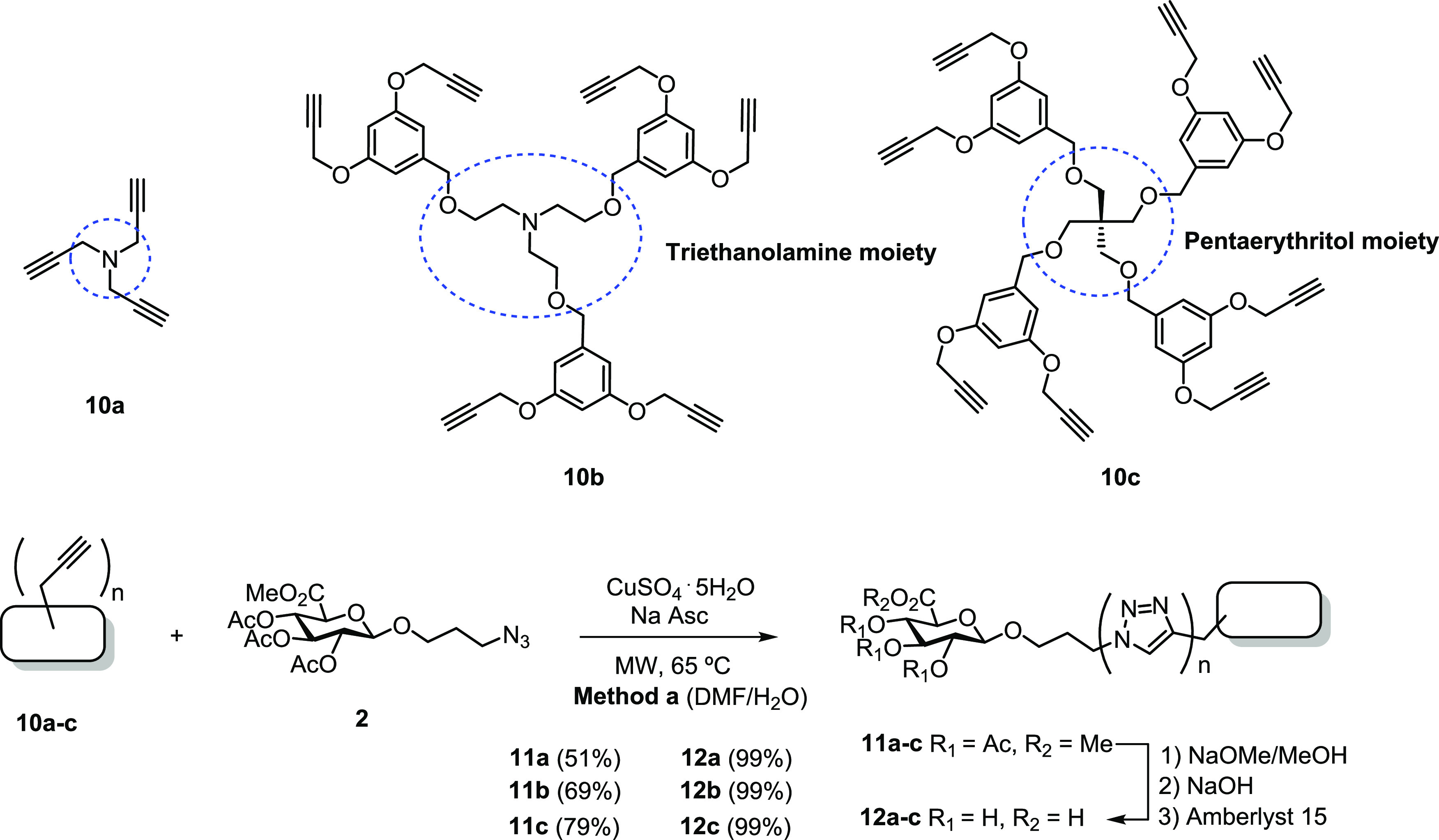
Synthesis of Flexible Scaffold-Based GlcA Dendrimers

The latter set of GlcA dendrimers was constructed from commercially available tri(prop-2-yn-1-yl)amine (**10a**), triethanolamine (**10b**), and pentaerythritol (**10c**) as flexible scaffolds (Scheme 4). Triethanolamine and pentaerythritol were subsequently branched with bisalkynyl-functionalized aryl groups. We hypothesized that compounds **10b** and **10c** bearing additional 3,5-bis(prop-2-yn-1-yloxy)benzene groups as branching elements might facilitate binding to the viral envelope protein through π–π stacking interactions.^26^

Aliphatic **10a** and mixed aliphatic–aromatic scaffolds **10b** and **10c** were linked to protected GlcA-based azide **2** via the above-mentioned CuAAC/deprotection sequence to give rise water-soluble GlcA dendrimers **12a**–**c**, respectively.

All compounds were purified by flash chromatography. The complete absence of terminal alkyne proton resonance at δ 2.2–2.5 ppm and the appearance of a new singlet at δ 8.1–7.7 ppm attributable to the triazole moiety suggested successful completion of the reaction.

### Surface Resonance Plasmon Studies

In order to evaluate the ability of GDs **4a**–**f**, **9a**–**c**, and **12a**–**c** to interact with dengue virus envelope protein 2 (DENV2), an SPR binding study was carried out. As mentioned above, previous studies have demonstrated that a tetravalent GlcA dendron binds efficiently to the DENV envelope protein with a *K*_D_ value of 22 μM.^12^

**Figure 1:**
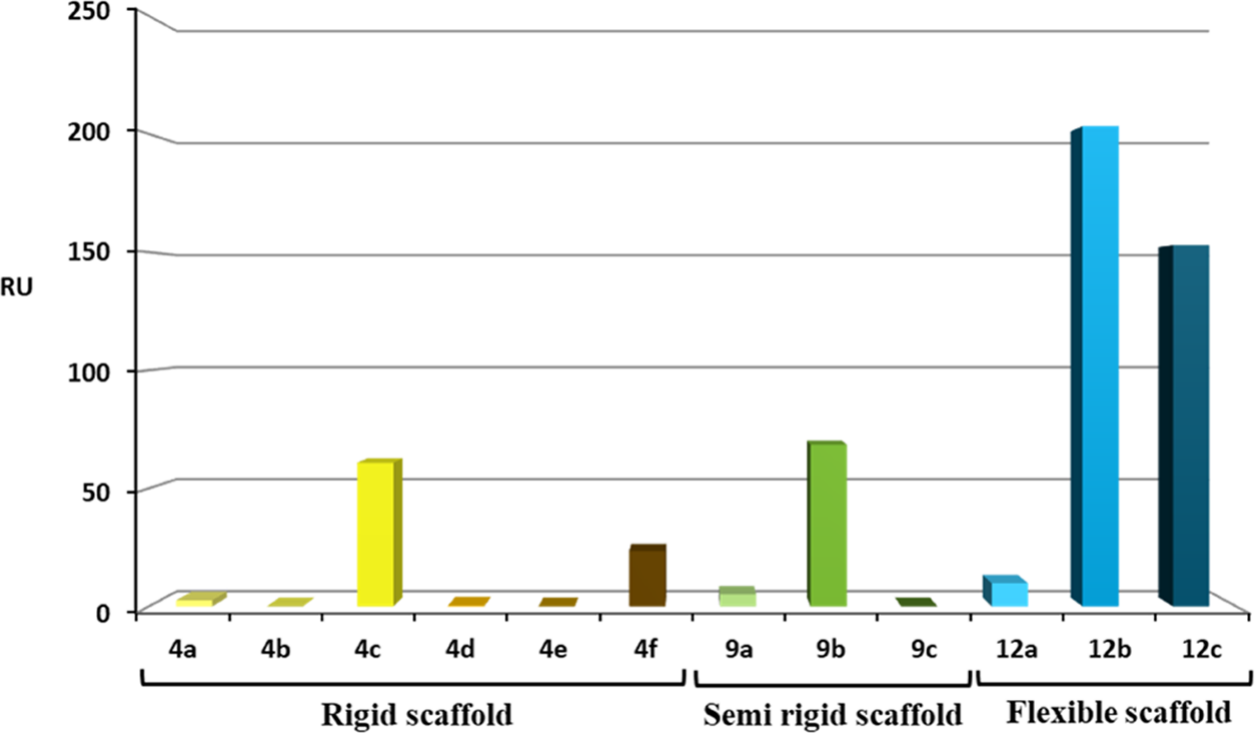
SPR measurement of GlcA GDs binding to immobilized DENV2 at 100 μM.

Compounds **4a**–**f**, **9a**–**c**, and **12a**–**c** were flowed on immobilized DENV2 at a concentration of 100 μM (Figure 1). As can be seen in Figure 1, the GDs binding to DENV2 depends on the scaffold type and valency. In the set of rigid GlcA GDs, compounds **4a**,**b** and **4d**–**f** showed a negligible binding response (2.6–0.0 RU) with the DENV2 envelope protein, whereas compound **4c** displayed a greater response (60.8 RU). Lastly, in the set of semirigid GDs, compounds **9a** and **9c** showed an insignificant binding response (**9a**, 5.0 RU and **9c**, 0.0 RU) with the DENV2 envelope protein, while compound **9b** evinced a good response (68.5 RU). Finally, in the set of flexible GDs, compound **12a** showed a low binding response (9.8 RU), but compounds **12b** and **12c** displayed a higher response, 200 and 150 RU, respectively (Figure 1).

Thus, compounds with a flexible scaffold and bearing six (compound **12b**, 203.4 RU) and eight GlcAs (compound **12c**, 153 RU) showed stronger binding than the rigid (compounds **4d**, 0.3 RU; **4e**, 0.0 RU with six GlcAs and **4f**, 23.4 RU with eight GlcAs) or semirigid scaffolds (compounds **9b**, 68.5 RU with six GlcAs and **9c**, 0.0 RU with eight GlcAs) at equal valency.

On the one hand, the high interaction obtained for GD **12b** having six GlcAs (*K*_D_ = 0.487 μM) and compound **12c** bearing eight GlcAs (*K*_D_ = 0.624 μM) suggested that the presence of the central aliphatic flexible scaffold favored the correct orientation of the negatively charged GDs to interact with the key residues in the binding site of the DENV2 protein. On the other hand, valency also has an important influence on the binding mode, and GDs with a higher number of GlcA (compounds **12b** and **9b** with six GlcAs and **12c** with eight GlcAs) showed a greater interaction.

These results indicate that the nature of the dendritic core and the valency exert influence on the interaction of these GDs and the DENV2 envelope protein. However, more SPR studies are required to better assess the behavior of these GDs. Then, different concentrations of the best compounds with flexible (**12b** and **12c**), semirigid (**9b**), and rigid scaffolds (**4c**) were flowed over the chip containing immobilized DENV2.

**Figure 2:**
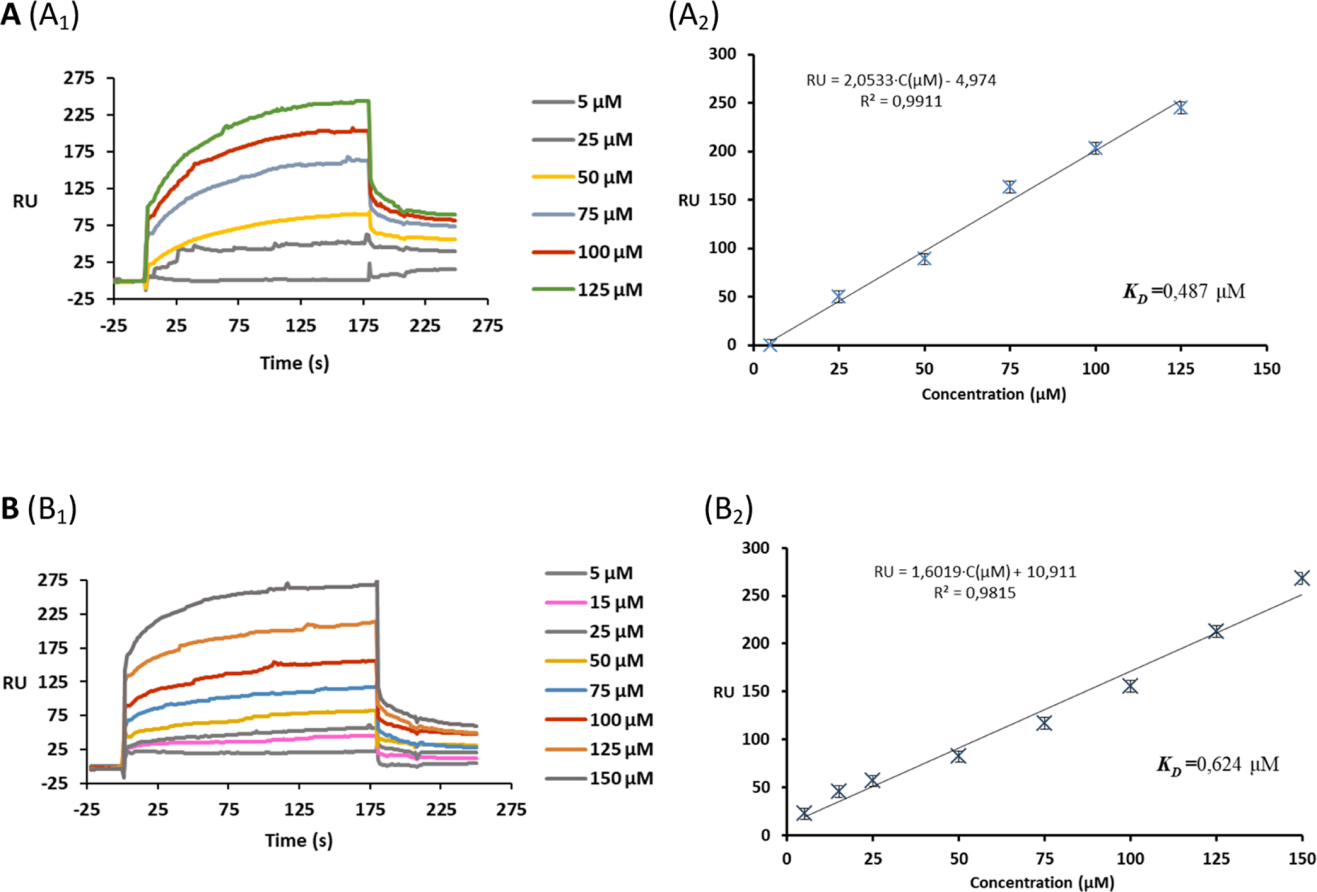
Binding response of different concentrations of GDs **12b** (A_1_) and **12c** (B_1_) with DENV2 immobilized on the CM4 chip, showing association and dissociation phases. Responses were reference-subtracted and blank-corrected. Steady-state affinity study of the interaction between GDs **12b** (A_2_) and **12c** (B_2_) and DENV2 immobilized on a CM4 chip.

Sensograms obtained showed increased interaction profiles with GDs **12b** and **12c** (Figure 2A). Using steady-state analysis of SPR measurements for the interaction between GDs (**12b** and **12c**) and DENV2, the dissociation constants (*K*_D_) were found to be *K*_D_ = 0.487 and 0.624 μM, respectively (Figure 2A,B).

**Figure 3:**
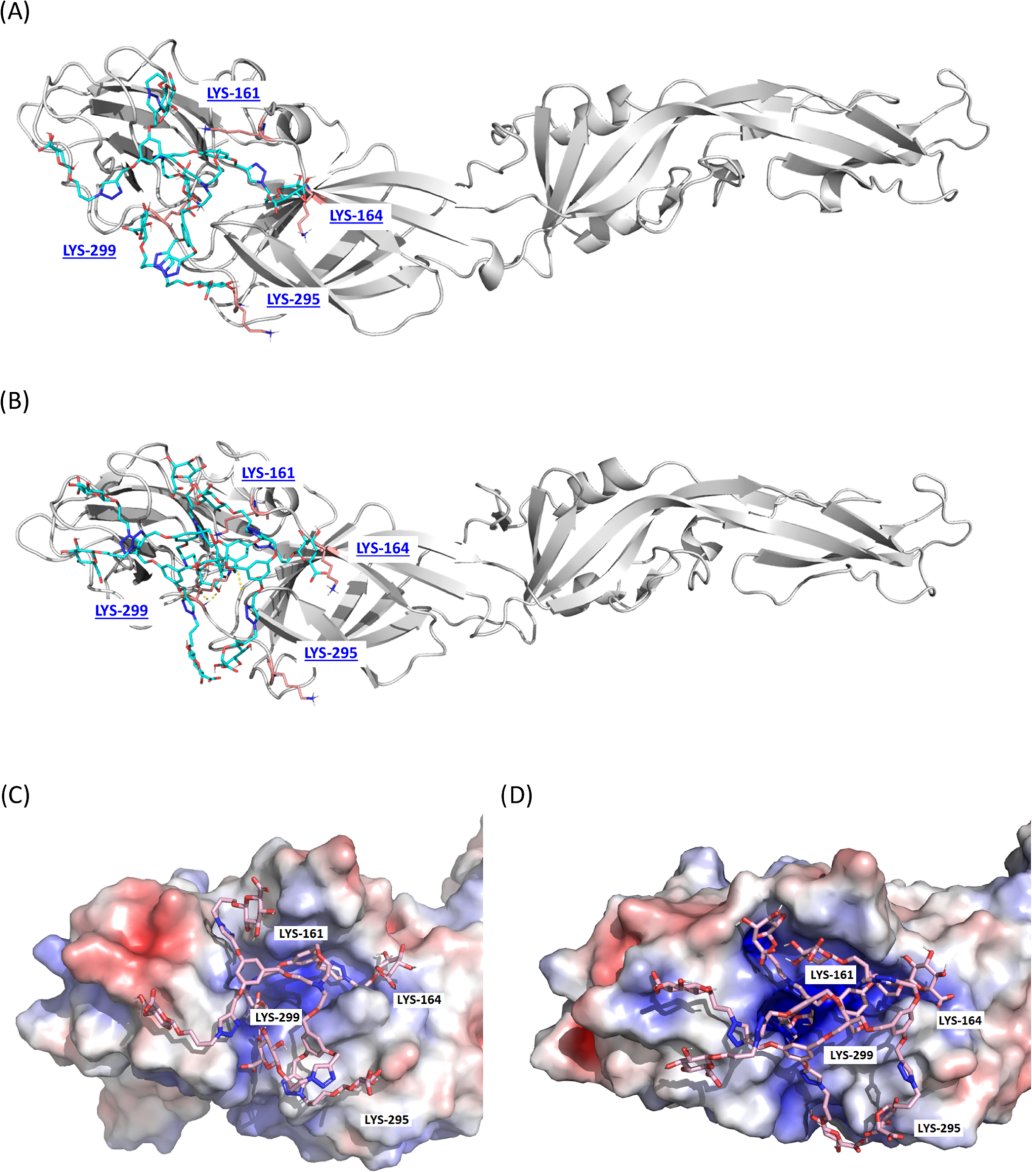
Final MD pose of GDs **12b** (A, C) and **12c** (B, D) in DENV2. In (A) and (B) representation, the DENV2 protein is depicted as a white cartoon, lysine residues as pink sticks, and GDs as glue sticks. In (C) and (D) representation, GDs are shown as pink sticks and the protein is shown as an electrostatic surface calculated by APBS (negatively charged surface in red; positively charged surface in blue; hydrophobic surface in white). The difference in intensity of the colors is due to the position of the side chain residues on the surface after the simulation.

The interaction of GDs **9b** and **4c** with the DENV2 envelope protein was also analyzed in the concentration range of 25–250 μM to allow the determination of the binding constant. In the steady-state analysis, the dissociation constants *K*_D_ were found to be 1.75 μM and 1.63 μM, respectively (Figure S1 in the Supporting Information).

Our results indicated a strong interaction between DENV2 and both GDs, **12b** and **12c**, which are 3.6–3.4 and 2.8–2.6 times better than that of compounds **4c** and **9b** and 45 and 35 times better than the one obtained in previous studies with a rigid tetravalent GlcA dendron (*K*_D_ = 22 μM).^12^

In a previous study, Marcks and co-workers studied the interaction of DENV2 to heparin (or heparan sulfate) by SPR and found a *K*_D_ of 56 nM under the same experimental conditions used in the current study.^8^ Our results for GDs **12b** and **12c** binding with the immobilized DENV2 envelope protein gave *K*_D_ values of 0.487 and 0.624 μM, respectively, weaker than that observed for heparin. The interaction of heparin with the immobilized DENV2 envelope protein yielded an 8.6–11-fold higher *K*_D_ than GDs **12b** and **12c**. This can be explained by the fact that heparin can make more than one interaction with the immobilized DENV2.

### Molecular Modeling

In order to explain the difference in the interactions observed in the binding of DENV2 to GDs **12b** and **12c**, a closer look at the DENV2–GD complex was carried out using molecular modeling studies. A structural rationale for tetravalent GlcA dendron and DENV2 interactions has already been published.^12^ Briefly, after docking and molecular dynamics (MD) simulations of the tetravalent GlcA dendron in a 3D validated model of the DENV2 protein, we concluded that the main interactions were due to electrostatic interactions between the carboxylate groups of GlcA-GD and LYS-299 and LYS-295 in the GAG-binding region of DENV2, which are the key residues involved in the heparin-binding region.^12^

In the present work, in order to try to explain the results obtained in SPR experiments, we performed docking and MD simulations of the GDs with the best interaction values in the GAG-binding region of DENV2. For this purpose, we used the already validated 3D model of DENV2 and focused the docking on the cavity formed by the sequence ^294^DKLQL**K**GMSYSMCTGKKFKVVVKEIAET^319^ containing the lysines responsible for GAG binding. After docking, the best-fitting poses of GDs **12b** and **12c** in the DENV2 cavity were chosen for MD simulations. After 50 ns of simulations, both complexes were structurally and energetically stables. The results of MD simulations suggest that the protein complexes (DENV2-**12b** and DENV2-**12c**) were stables, with RMSD values below 4 Å and without big fluctuation on particular residues, and there were no remarkable fluctuations affecting the ligand binding site (Figures S2–S5 in the Supporting Information). Regarding the GDs, we could observe fluctuations in the RMSD values due to the rearrangement of the conformation with respect to the docking pose, especially in GD **12b** (∼8 Å). However, once the new fit is adopted, the structure of both complexes was stable during the rest of the simulation; therefore, both dendrimers were capable of binding DENV2.

The binding energy results show that both complexes were energetically stable throughout the dynamics, with a better value between DENV2 and **12b** (−130 kcal/mol) than that between DENV2 and **12c** (−120 kcal/mol) (Figures S6 and S7 in the Supporting Information), values in agreement with SPR results.

Binding energy decomposition results suggest that one branch of the GDs would be lodged at the cavity where LYS-299 is located, and the rest of the molecule interacts with the surface of the protein by important van der Waals and electrostatic interactions including multiple hydrogen bonds (Figure 3 and Table S1 in the Supporting Information). Nevertheless, there is no observed interaction with LYS-295, even though the docking calculations initially positioned compounds **12b** and **12c** correctly for such an interaction.

The main interactions took place between the carboxylate groups of dendrimers and LYS-299, with values of −41.4 (kcal/mol) and −20.7 (kcal/mol) for GDs **12b** and **12c**, respectively. Moreover, **12b** has better interactions with the residues involved in the hotspot sequence for carbohydrate interactions with DENV2 (Figure S8 in the Supporting Information). In addition to the multiplicity of the dendrimers, the better fit of GD **12c** in the DENV2 cavity suggests that the flexibility of the core in the dendrimer is a key point for the interaction, which provides an explanation of the greater interaction observed for compound **12b** (*K*_D_ = 0.487 μM) compared to **12c** (*K*_D_ = 0.624 μM).

**Figure 4:**
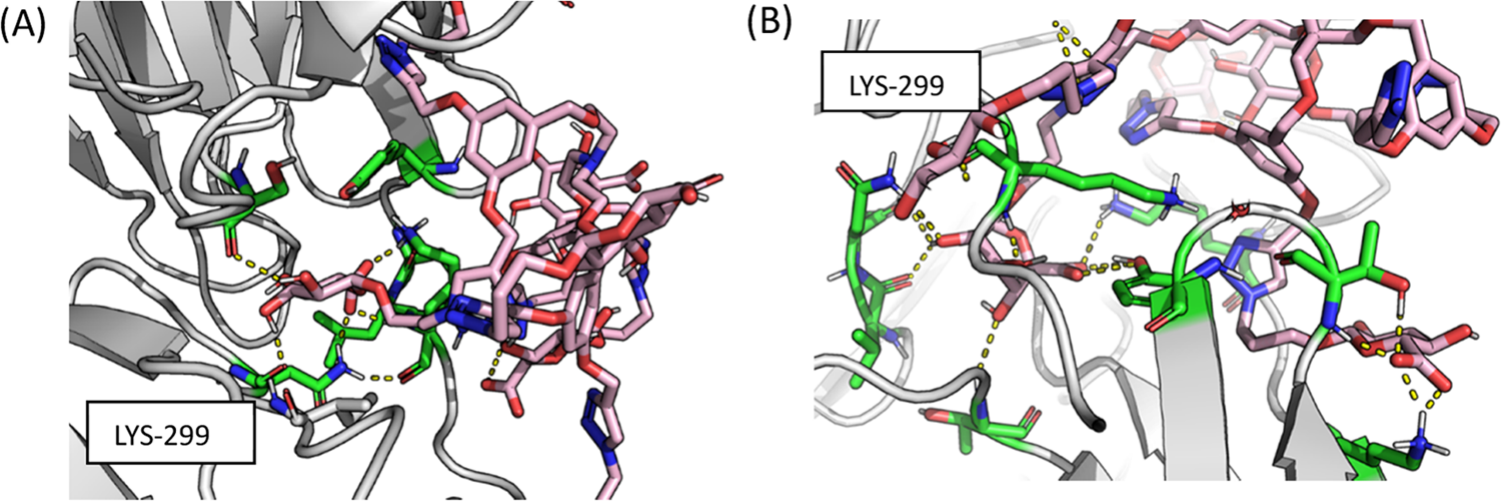
Final poses of DENV2 bound to dendrimers **12b** (A) and **12c** (B). Protein is shown in cartoon mode gray with the relevant residues shown as green sticks. Dendrimers are represented as pink sticks, and the electrostatic interactions are represented as yellow dash lines.

The geometry of compound **12b** enables the formation of a salt bridge interaction with the protonated nitrogen of LYS-299 of DENV2 and one of their GlcAs. Also noteworthy are the electrostatic interactions between the other GlcA of the dendrimer with LYS-164 (−16 kcal/mol), THR-180 (−12,7 kcal/mol), GLU-178 (−7,4 kcal/mol), SER-302 (−11,8 kcal/mol), TYR-303 (−10,7 kcal/mol), ASN-359 (−13,4), and LYS-161 that interact with the core (−13 kcal/mol; Figure 4).

In the case of compound **12c**, there is also a very important electrostatic interaction with LYS-299 but not so strong; the main interactions are also with the GlcA moiety as the interaction with LYS-164 (−14,7 kcal/mol), THR-180 (−13,9 kcal/mol), and LYS-161 (−29,8 kcal/mol) that also interact with the core.

## Conclusions

In this report, the efficient synthesis of three sets of GlcA-based GDs bearing different levels of multivalency, polarity, and structural features were described using rigid (**1a**–**f**), semirigid (**7a**–**c**), and flexible scaffolds (**10a**–**c**) and Cu-catalyzed click chemistry. Their chemical synthesis was performed sequentially in three stages: (a) the chemical synthesis of the alkynyl scaffolds, (b) assembling with fully protected GlcA-based azide units by using a MW-assisted CuAAC protocol, and (c) sequential deprotection of hydroxyl and carboxylic acid groups. The CuAAC coupling conditions were optimized with these scaffolds, and maximum conversions were obtained at short reaction times (90–120 min).

GlcA GDs **4a**–**f** (rigid), **9a**–**c** (semirigid), and **12a**–**c** (flexible) were evaluated in their ability to interact with DENV2 using surface plasmon resonance technology. The binding capacity of GDs **4a**–**f**, **9a–c**, and **12a** is relatively low compared to GlcA GDs **12b**–**c**, which confirms the importance of the flexibility of the scaffold in such interactions. At the same time, valency is another factor to take into consideration in the interaction between GlcA GDs and DENV2; higher binding was observed for the GDs bearing six GlcAs (*K*_D_ = 0.487 μM) and eight GlcAs (*K*_D_ = 0.624 μM), i.e., compounds **12b** and **12c**, respectively. There is therefore no major difference between these two GDs, which indicates that the main factor that increases the affinity is the type of scaffold and flexibility.

Molecular modeling provided an explanation for the difference in binding observed between GDs **12b** and **12c** and the GAG-binding region of DENV2, which is a basic-residue-rich region of DENV2. In a previous study, we demonstrated that the main type of interactions that took place between a GlcA glycodendron and the protein were electrostatic through LYS-299 and LYS-295.^12^ Based on this study, we envision that with GDs **12b** and **12c**, we would have the same type of behavior. The evaluation of the interaction energies of the residues involved in such binding shows that the main interactions took place between the carboxylate groups of GDs and LYS-299 (−41.4 kcal/mol for **12b** and −20.7 kcal/mol for **12c**). Moreover, the geometry of GD **12b** enables to have better interactions with LYS-299, as well as other interactions with surrounding residues, which increases the strength of the binding with GD **12b**. These results indicate that the flexibility of the scaffold in the dendrimer is a key point for the interaction with DENV2 and the higher affinity observed for compound **12b** (*K*_D_ = 0.487 μM) compared to **12c** (*K*_D_ = 0.624 μM).

This novel multivalent approach using a simple monosaccharide GlcA as the ligand and the appropriate flexible scaffold and valency has shown to be an excellent strategy to develop GDs that strongly interact with DENV2. Based on these studies, analogous elaboration of scaffolds with proper geometry and valency could enable the generation of multivalent antagonists selective for a variety of enveloped virus protein-recognizing HS receptors.

## Experimental Section

### General Methods

Unless noted otherwise, all manipulations were carried out under an argon atmosphere. All reagents were obtained from commercial sources and used without further purification. Solvents were HPLC or synthesis grade and used without further purification. A Monowave 50, 315 W, Anton Paar Microwave was employed for microwave-assisted CuAAC procedures. ^1^H and ^13^C NMR spectra were recorded at 250, 300, 500, or 700 MHz (^1^H NMR) and at 62.5, 75, or 100 MHz (^13^C NMR) using CDCl_3_ and DMSO-d_6_ as solvents with the residual solvent signal as the internal reference (CDCl_3_, 7.26 and 77.0 ppm and DMSO-d_6_, 2.50, and 39.5 ppm). The following abbreviations are used to describe peak patterns when appropriate: s (singlet), d (doublet), t (triplet), q (quadruplet), m (multiplet), and br (broad). Complete signal assignments from 1D and 2D NMR spectroscopy were based on COSY, HSQC, and HMBC correlation experiments. Thin layer chromatography (TLC) was carried out on aluminum sheets coated with silica gel 60 F254 (Merck). TLC plates were inspected under UV light (λ = 254 nm) and developed by treatment with sulfuric acid in methanol (10%) or by KMnO_4_/H_2_O solution, followed by heating. Flash column chromatography was performed by using silica gel (230–400 mesh). Mass spectra were recorded using electrospray (ES) chemical ionization techniques in positive mode unless noted otherwise.

Dengue virus envelope protein 2 (DENV2) was purchased from the Native Antigen Company. SPR sensor chips CM4 (carboxymethylated dextran) and other reagents used in SPR experiments were purchased from GE Healthcare. SPR experiments were performed with a Biacore 3000.

#### General Procedure for a Microwave-Assisted CuAAC Reaction: Synthesis of Compounds **3a**–**f**, **8a**–**c**, and **11a**–**c**

CuSO_4_·5H_2_O (0.20 equiv/alkyne) and sodium ascorbate (0.35–0.4 equiv/alkyne) were sequentially added to a solution of the appropriate alkyne (1.0 equiv) and GlcA-derived azide **2** (1.15–1.5 equiv/alkyne) in the appropriate solvent (DMF/H_2_O 98:2; DMSO/H_2_O 5:1; or DMA/H_2_O 98:2, details are described in the Supporting Information; 2 mL) at room temperature. The resulting mixture was placed in a Monowave 50 microwave (Anton Paar) at 315 W and irradiated at 65 °C until completion of the reaction (TLC analysis). After cooling to room temperature, the reaction mixture was diluted with AcOEt (20 mL) and washed with a solution of Na_2_EDTA/NaOH aq. (2 × 10 mL) and brine (2 × 10 mL). The combined organic layers were dried over anhydrous Na_2_SO_4_, filtered, and concentrated under vacuum. The residue was purified by flash chromatography on silica (hexane/AcOEt 1:1 to DCM/MeOH 10:1 or AcOEt/MeOH 10:1, details are described in the Supporting Information), affording corresponding acetyl-protected dendrimers **3a**–**f**, **8a**–**c**, and **11a**–**c**.

#### General Method for Acetal and Methyl Ester Deprotection: Synthesis of Compounds **4a**–**f**, **9a**–**c**, and **12a**–**c**

To a solution of the corresponding protected dendrimer in anhydrous MeOH, a 0.5 N NaOMe/MeOH solution was added (1.5 equiv of NaOMe/AcO), and the resulting mixture was stirred for 4 h. Then, a 0.2 M NaOH aq solution was added (1.5 equiv/CO_2_Me), and the mixture was stirred overnight. Amberlyst 15 was added at room temperature until pH = 5, the mixture was filtered, and the solvent was removed under vacuum, affording corresponding unprotected GDs **4a–f**, **9a**–**c**, and **12a**–**c.**

### Surface Resonance Plasmon Studies

SPR experiments were performed at 25 °C with a Biacore 3000 (GE Healthcare). PBST (10 mm phosphate, pH 7.40, 150 mm NaCl, and 0.005% v/v surfactant P20) was used as a running buffer. A solution with the dengue virus serotype 2 protein (DENV2) was adjusted to 20 μg/mL in 10 mM citrate pH 4.00 buffer, and the protein was immobilized in flow cell 1 of a CM4 sensor chip by following the amine coupling method according to the manufacturer’s instructions. Prior to injection over the sensor chip, DENV2 was mixed with a 4-fold molar excess of HS, for 30 min at 4 °C, to protect the GAG-binding domains of the envelope protein. The immobilization response was 2000 RU, and then, the sensor surface was washed with 1 M NaCl to remove the bound HS. Sensor chip flow cell 2 was activated, blocked, and used as a reference surface. Blank samples and concentration series were injected on a CM4 chip at a flow rate of 40 μL/min for 180 s, and dissociation was registered for 180 s.

Initially, compounds **4a**–**f**, **9a**–**c**, and **12a**–**c** were flowed on the immobilized DENV2 at a 100 μM concentration. Then different concentration of GDs (5–125 μM) were flowed on the immobilized DENV2 (compound **12b**: 5–125 μM; **12c**: 5–150 μM; **9b**: 25–250 μM; **4c**: 25-250 μM).25–250 μM). Data processing and analysis were carried out with Biaevaluation v.4.1.1 (GE Healthcare).

All signals were blank-subtracted, reference-corrected, and globally adjusted to an adequate kinetic model to obtain binding parameters. As expected, the registered sensorgrams showed complex binding profiles consistent with the multivalent nature of the glycodendrimer–DENV2 interaction that failed to globally fit to conventional binding models. Instead, a steady-state analysis kinetic analysis was carried out assuming the 1:1 Langmuir model. The value of *K*_A_ is obtained by fitting a plot of RU in the plateau region against the GD concentration, which generates the following equations.
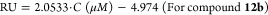

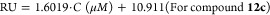

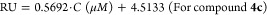

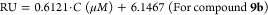
*K*_D_ is calculated as the inverse of *K*_A_ (*K*_D_ = 1/*K*_A_).

### Molecular Modeling Studies

Molecular modeling procedures are described in the Supporting Information.

## Supporting Information Available

Synthesis and characterization of alkynyl scaffolds and glucuronic acid glycodendrimers; and computational methods and figures (PDF)
