# Supplementary material for: Exploring Rigid and Flexible Scaffolds to Develop Potent Glucuronic Acid Glycodendrimers for Dengue Virus Inhibition
Source: Bioconjug Chem. 2023 Nov 15;35(1):34–42. doi: 10.1021/acs.bioconjchem.3c00309 (PMC10797590; doi:10.1021/acs.bioconjchem.3c00309)
Supplement: Supplementary file 1 — bc3c00309_si_001.pdf [file bc3c00309_si_001.pdf]

Supporting Information

Exploring Rigid and Flexible Scaffolds to Develop Potent Glucuronic Acid Glycodendrimers for Dengue virus Inhibition

Alejandro Merchán, Pedro Ramírez-López, Carlos Martínez, José Ramón Suárez, Almudena Perona, and María J. Hernáiz*

Departamento de Química en Ciencias Farmacéuticas, Facultad de Farmacia, Universidad Complutense de Madrid, Plz. Ramón y Cajal s/n, Madrid, C.P. 28040, España

Corresponding author's email address: mjhernai@ucm.es

Table of Contents

1. Preparation of Alkynyl Scaffolds **1a-f, 7a-c and 10a-c**. S2-S8
2. GlcA Glycodendrimers Synthesis and characterization (**3a-f, 8a-c and 11a-c**) S8-S16
3. Synthesis and characterization of compounds **4a-f, 9a-c and 12a-c** S17-S24
4. Figure S1 S-25
5. Computational Methods S26-S27
6. Figures S2-S8 S28-S29
7. Table S1 S30
8. References S31
9. NRM Spectra of final GDs……………………………………………………S32

1. Preparation of Alkynyl scaffolds 1a-f:

The scaffolds mentioned below were prepared using reported procedures:

(3,5-bis(prop-2-yn-1-yloxy)phenyl)methanol,[1]
phloroglucinol triacetate[2]
(3,5-bis(prop-2-yn-1-yloxy)phenyl)chloride[3]
1-(bromomethyl)-3,5-bis(prop-2-yn-1-yloxy)benzene,[1]
(3,4,5-tris(prop-2-yn-1-yloxy)phenyl)methanol,[4]
(3,5-bis((3,5-bis(prop-2-yn-1-yloxy)benzyl)oxy)phenyl)methanol,[1]
5,5'-(((5-(bromomethyl)-1,3-phenylene)bis(oxy))bis(methylene))bis(1,3-bis(prop-2-yn-1-yloxy)benzene),[1]
2,4,6-tri(prop-2-yn-1-yl)-1,3,5-triazine,[5]
1,3,5-tris(prop-2-yn-1-yloxy)benzene,[6]
and 2,3,6,7,10,11-hexakis(prop-2-yn-1-yloxy)triphenylene.[7]

Synthesis of methyl 3,4,5-tris(prop-2-yn-1-yloxy)benzoate

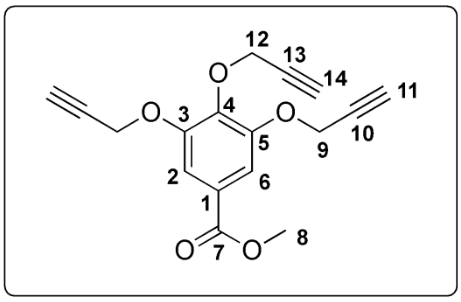

A stirred mixture of methyl 3,4,5-trihydroxybenzoate (2.45 g, 13.30 mmol, 1.0 equiv), propargyl bromide (7.12 g, 59.87 mmol, 4.5 equiv) and anhydrous K_2CO_3 (8.27 g, 59.87 mmol, 4.5 equiv) in dry DMF (25 mL) was heated at 60 ºC under argon for 24 h. The reaction crude was allowed to reach room temperature and then AcOEt (100 mL) was added. The mixture was washed with brine (2 x 100 mL), a 0.5 N NaOH aq solution (2 x 100 mL) and water (2 x 100 mL) and the resulting organic phase was dried over anhydrous Na_2SO_4, filtered and concentrated under vacuum affording methyl 3,4,5-tris(prop-2-yn-1-yloxy)benzoate as beige solid (3.77 g, 95%).

^{1}H-NMR ($CDCl_3$, 250 MHz): **δ** = 7.47 (s, 2, H-2, H-6), 4.82 (d, J = 2.4 Hz, 2H, H-12), 4.81 (d, J = 2.4 Hz, 4H, H-9), 3.91 (s, 3H, H-8), 2.53 (t, J = 2.4 Hz, 2H, H-11), 2.46 (t, J = 2.4 Hz, 1H, H-14).

^{13}C-NMR ($CDCl_3$, 62.5 MHz): **δ** = 166.3 (C, C-7), 151.3 (C, C-3, C-5), 141.0 (C, C-4), 125.8 (C, C-1), 109.8 (CH, C-2, C-6), 78.7 (C, C-13), 78.0 (C, C-10), 76.3 (CH, C-11), 75.7 (CH, C-14), 60.3 (CH_2, C-12), 57.0 (CH_2, C-9), 52.4 (CH_3, C-8).

Synthesis of 5-(bromomethyl)-1,2,3-tris(prop-2-yn-1-yloxy)benzene

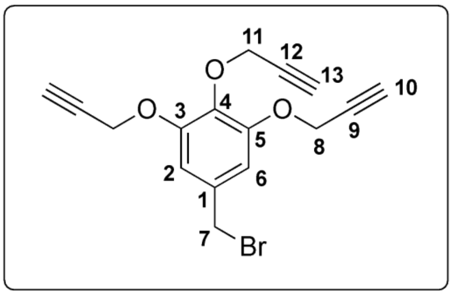

To a stirred solution of the (3,4,5-tris(prop-2-yn-1-yloxy)phenyl)methanol[4] (1.86 g, 6.88 mmol, 1.0 equiv) in dry THF (50 mL) was added CBr_4 (2.85 g, 8.60 mmol, 1.25 equiv) followed by the portionwise addition of PPh_3 (2.26 g, 8.60 mmol, 1.25 equiv). The reaction was stirred at room temperature for 1 h and then quenched with 50 mL of water. THF was evaporated, and the crude product was extracted with dichloromethane (2 × 50 mL). The organic layer was dried with Na_2SO4 and evaporated to dryness. The residue was purified by flash chromatography (hexanes/DCM 2:1 to 1:2) affording 5-(bromomethyl)-1,2,3-tris(prop-2-yn-1-yloxy)benzene as a white solid (2.05 g, 90%).

^{1}H-NMR ($CDCl_3$, 250 MHz): **δ** = 6.79 (s, 2, H-2, H-6), 4.76 (d, J = 2.4 Hz, 4H, H-8), 4.72 (d, J = 2.4 Hz, 2H, H-11), 4.45 (s, 2H, H-7), 2.53 (t, J = 2.4 Hz, 2H, H-10), 2.47 (t, J = 2.4 Hz, 1H, H-13).

^{13}C-NMR ($CDCl_3$, 62.5 MHz): **δ** = 151.7 (C, C-3, C-5), 137.8 (C, C-4), 133.8 (C, C-1), 109.5 (CH, C-2, C-6), 79.1 (C, C-12), 78.3 (C, C-9), 76.2 (CH, C-10), 75.5 (CH, C-13), 60.5 (CH_2, C-11), 57.1 (CH_2, C-8), 33.8 (CH_3, C-7).

a) **Preparation of scaffold 1a**

Scaffold **1a** was prepared using reported procedure.[5]

b) **Synthesis of 1b**

***N*^{2},*N*^{2},*N*^{4},*N*^{4},*N*^{6},*N*^{6}-hexa(prop-2-yn-1-yl)-1,3,5-triazine-2,4,6-triamine**

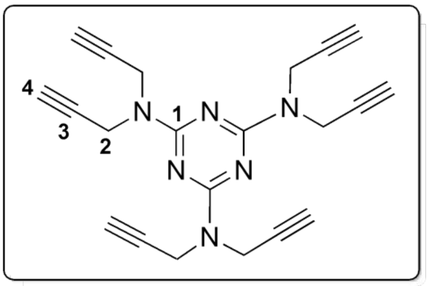

Dipropargylamine (0.192 mL, 1.862 mmol, 3.3 equiv) and NEt_3 (0.236 mL, 1.692 mmol, 3.0 equiv) were sequentially added dropwise for 10 min at room temperature to a solution of cyanuric chloride (104 mg, 0.564 mmol, 1.0 equiv) in dry toluene (4.0 mL) under argon. After stirring the resulting brownish reaction mixture at reflux for 20 h the hot crude mixture was immediately filtered under vacuum, giving rise to a white powder which was purified through a short pad of SiO_2 (hexanes/AcOEt 5:1) affording hexaalkyne **1b** as a white solid (120 mg, 64 %)

^{1}H-NMR ($CDCl_3$, 250 MHz): **δ** = 4.55 (d, J = 2.4 Hz, 6H, H-2), 2.19 (t, J = 2.4 Hz, 3H, H-4).

^{13}C-NMR ($CDCl_3$, 62.5 MHz): **δ** = 164.8 (C-1), 79.5 (C-3), 71.6 (C-2), 34.8 (C-4).

c) Synthesis of 1c

Scaffold **1c** was prepared using reported procedure.[6]

d) Synthesis of 1d

Synthesis of 1,3,5-tris((3,5-bis(prop-2-yn-1-yloxy)benzyl)oxy)benzene

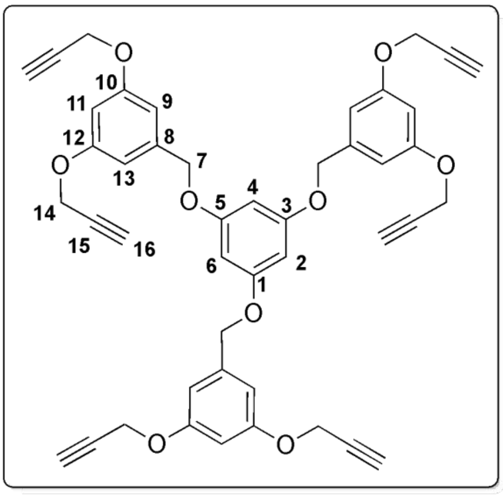

NaH 60% suspension (75 mg, 1.87 mmol, 7.2 equiv) was added to a mixture of phloroglucinol triacetate (66 mg, 0.26 mmol, 1.0 equiv)[2] and (3,5-bis(prop-2-yn-1-yloxy)phenyl)chloride[3] (220 mg, 0.937 mmol, 3.6 equiv) in dry DMF (4.0 mL) at room temperature under argon. The mixture was then cooled to 0 ºC and H_2O was added (14 µL, 0.78 mmol, 3.0 equiv). The crude mixture was stirred at room temperature for 3 h, diluted with AcOEt (5 mL) and washed with water (5 mL x 2) and brine (5 mL x 2) and the resulting organic phase was dried over anhydrous Na_2SO_4, filtered and concentrated under vacuum affording hexaalkyne **1d** as white solid (150 mg, 83%).

^{1}H-NMR ($CDCl_3$, 250 MHz): **δ** = 6.66 (d, J = 2.3 Hz, 6H, H-9, H-13), 6.56 (t, J = 2.3 Hz, 3H, H-11), 6.21 (s, 3H, H-2, H-4, H-6), 4.95 (s, 6H, H-7), 4.67 (d, J = 2.4 Hz, 12H, H-14), 2.52 (t, J = 2.4 Hz, 6H, H-16).

^{13}C-NMR ($CDCl_3$, 62.5 MHz): **δ** = 160.5 (C, C-5), 159.0 (C, C-10, C-12), 139.5 (C, C-8), 107.0 (CH, C-9, C-13), 101.9 (CH, C-11), 95.2 (CH, C-2, C-4, C-6), 78.4 (C, C-15), 75.9 (CH, C-16), 69.9 (CH_2, C-7), 56.1 (CH_2, C-14).

e) Synthesis of 1e:

Scaffold **1e** was prepared using reported procedure.[7]

f) Synthesis of 1f:

Synthesis of 1,2,4,5-tetra((3,5-bis(prop-2-yn-1-yloxy)benzyl)oxy)benzene

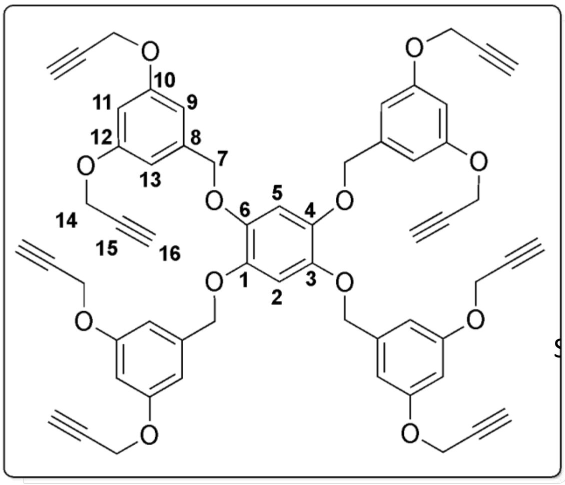

A mixture of benzene-1,2,4,5-tetraol (76 mg, 0.535 mmol, 1.0 equiv), 1-(bromomethyl)-3,5-bis(prop-2-yn-1-yloxy)benzene[1] (896 mg, 3.209 mmol, 6.0 equiv) and anhydrous K_2CO_3 (296 mg, 2.14 mmol, 4.0 equiv) in dry DMF (5.0 mL) was heated at 65 ºC under argon for 60 h. The reaction crude was allowed to

reach room temperature and then AcOEt (25 mL) was added. The mixture was washed with a 0.5 N NaOH aq solution (2 x 15 mL) and brine (2 x 20 mL) and the resulting organic phase was dried over anhydrous Na_2SO_4, filtered and concentrated under vacuum. The residue was purified by flash chromatography (hexanes/AcOEt 3:2) affording octaalkyne **1f** as a white solid (410 mg, 82%).

^{1}H-NMR ($CDCl_3$, 250 MHz): **δ** = 6.63 (d, *J* = 2.3 Hz, 8H, H-9, H-13), 6.54 (t, *J* = 2.3 Hz, 4H, H-11), 6.48 (s, 2H, H-2, H-5), 4.67 (d, *J* = 2.4 Hz, 16H, H-14), 4.64 (s, 8H, H-7), 2.53 (t, *J* = 2.4 Hz, 8H, H-16).

^{13}C-NMR ($CDCl_3$, 62.5 MHz): **δ** = 159.0 (C, C-10, C-12), 143.7 (C, C-1, C-3, C-4, C-6), 106.3 (CH, C-9, C-13), 101.9 (CH, C-2, C-5), 101.6 (CH, C-11), 78.5 (C, C-15), 75.8 (CH, C-16), 65.3 (CH_2, C-7), 56.0 (CH_2, C-14).

g) Preparation of scaffold 7a:

Synthesis of 5,5'-(oxybis(methylene))bis(1,3-bis(prop-2-yn-1-yloxy)benzene)

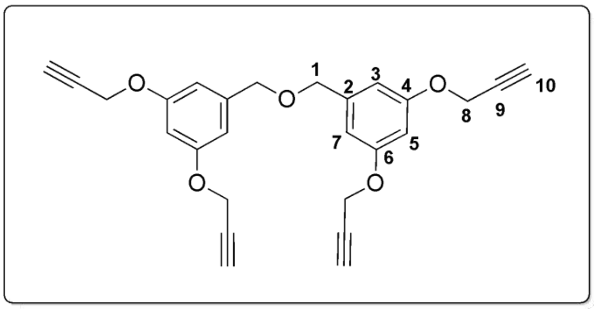

NaH 60% suspension (34.7 mg, 0.868 mmol, 1.25 equiv) was added to a solution of (3,5-bis(prop-2-yn-1-yloxy)phenyl)methanol[1] (150.0 mg, 0.694 mmol, 1.0 equiv) in dry DMF (3 mL) at 0 °C under argon. The reaction was allowed to reach room temperature for 30 min. Then 1-(bromomethyl)-3,5-bis(prop-2-yn-1-yloxy)benzene (290.4 mg, 1.041 mmol, 1.5 equiv) was added in portions for 5 min and the resulting mixture was heated at 50 °C for 20 h. The reaction crude was cooled to 0 ºC, quenched with a few drops of water, diluted with Et_2O (25 mL), washed with brine (2 x 20 mL) and the resulting organic phase was dried over anhydrous Na_2SO_4, filtered and concentrated under vacuum. The residue was purified by flash chromatography (hexanes/AcOEt 4:1 to 7:3) affording tetraalkyne **7a** as a white solid (233.3 mg, 81%).[1]

^{1}H-NMR ($CDCl_3$, 250 MHz): **δ** = 6.64 (d, *J* = 2.4 Hz, 4H, H-3, H-7), 6.55 (t, *J* = 2.4 Hz, 2H, H-5), 4.67 (d, *J* = 2.4 Hz, 8H, H-8), 4.50 (s, 4H, H-1), 2.54 (t, *J* = 2.4 Hz, 4H, H-10).

^{13}C-NMR ($CDCl_3$, 62.5 MHz): **δ** = 158.9 (C, C-4, C-6), 140.9 (C, C-2), 107.2 (CH, C-3, C-7), 101.7 (CH, C-5), 78.5 (C, C-9), 75.9 (CH, C-10), 71.9 (CH_2, C-1), 56.0 (CH_2, C-8).

h) Synthesis of 7b:

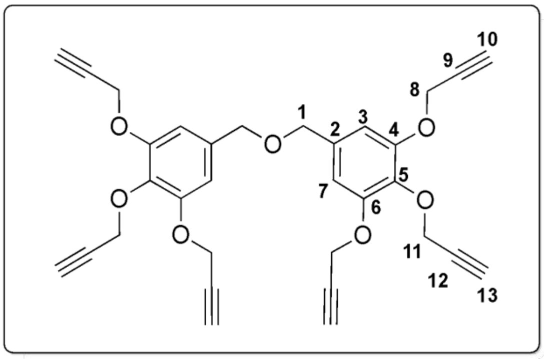

Synthesis of 5,5'-(oxybis(methylene))bis(1,2,3-tris(prop-2-yn-1-yloxy)benzene)

NaH 60% suspension (16.7 mg, 0.694 mmol, 1.25 equiv) was added to a solution of (3,4,5-tris(prop-2-yn-1-yloxy)phenyl)methanol[4] (150.0 mg, 0.555 mmol, 1.0 equiv) in dry DMF (3 mL) under argon at 0 °C. The reaction was allowed to reach room temperature for 30 min. Then 5-(bromomethyl)-1,2,3-tris(prop-2-yn-1-yloxy)benzene (277.5 mg, 0.833 mmol, 1.5 equiv) was added in portions for 5 min and the resulting mixture was heated at 50 °C for 40 h. The reaction crude was cooled to 0 ºC, quenched with a few drops of water, diluted with Et_2O (25 mL), washed with brine (2 x 20 mL) and the resulting organic phase was dried over anhydrous Na_2SO_4, filtered and concentrated under vacuum. The residue was purified by flash chromatography (hexanes/AcOEt 7:3 to 3:2) affording hexaalkyne **7b** as a white solid (231.5 mg, 80%).

^{1}H-NMR ($CDCl_3$, 250 MHz): **δ** = 6.77 (s, 4H, H-3, H-7), 4.75 (d, *J* = 2.4 Hz, 8H, H-8), 4.71 (d, *J* = 2.4 Hz, 4H, H-11), 4.50 (s, 4H, H-1), 2.51 (t, *J* = 2.4 Hz, 4H, H-10), 2.46 (t, *J* = 2.4 Hz, 2H, H-13)

^{13}C-NMR ($CDCl_3$, 62.5 MHz): **δ** = 151.7 (C, C-4, C-6), 136.6 (C, C-5), 134.5 (C, C-2), 108.0 (C, C-3, C-7), 79.2 (C, C-12), 78.6 (C, C-9), 76.0 (CH, C-10), 75.3 (CH, C-13), 71.9 (CH_2, C-1), 60.4 (CH_2, C-11), 57.1 (CH_2, C-8).

i) Synthesis of 7c

5,5',5'',5'''-((((oxybis(methylene))bis(benzene-5,1,3-triyl))tetrakis(oxy))tetrakis (methylene)) tetrakis(1,3-bis(prop-2-yn-1-yloxy)benzene)

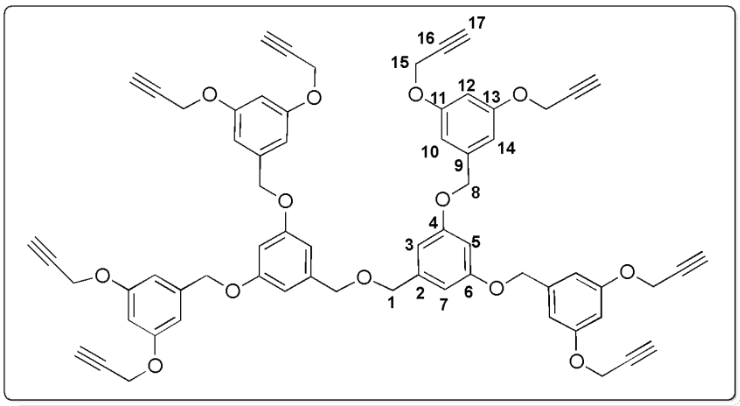

NaH 60% suspension (9.1 mg, 0.227 mmol, 1.25 equiv) was added to a solution of (3,5-bis((3,5-bis(prop-2-yn-1-yloxy)benzyl)oxy)phenyl) methanol[1] (97.6 mg, 0.182 mmol, 1.0 equiv) in dry DMF (2.5 mL) under argon at 0 °C. The reaction was allowed to reach room temperature for 30 min.

Then 5,5'-(((5-(bromomethyl)-1,3-phenylene)bis(oxy))bis(methylene))bis(1,3-bis(prop-2-yn-1-yloxy)benzene)[1] (163.7 mg, 0.273 mmol, 1.5 equiv) was added in portions for 5 min and the resulting mixture was heated at 50 °C for 48 h. The reaction crude was cooled to 0 ºC, quenched with a few drops of water, diluted with Et_2O (25 mL), washed with brine (2 x 20 mL) and the resulting organic phase was dried over anhydrous Na_2SO_4, filtered and concentrated under vacuum. The residue was purified by two sequential flash

chromatographies (hexanes/AcOEt 7:3 to 1:1 and hexanes/DCM 1:1 to DCM) affording octaalkyne **7c** as a white solid (134 mg, 70%).

^{1}H-NMR ($CDCl_3$, 250 MHz): **δ** = 6.67 (d, J = 2.3 Hz, 8H, H-10, H-14), 6.59 (d, J = 2.3 Hz, 4H, H-3, H-7), 6.55 (t, J = 2.3 Hz, 4H, H-12), 6.51 (t, J = 2.3 Hz, 2H, H-5), 4.98 (s, 8H, H-8), 4.66 (d, J = 2.4 Hz, 16H, H-15), 4.46 (s, 4H, H-1), 2.51 (t, J = 2.4 Hz, 8H, H-17).

^{13}C-NMR ($CDCl_3$, 62.5 MHz): **δ** = 159.9 (C, C-4, C-6), 158.9 (C, C-11, C-13), 140.8 (C, C-2), 139.5 (C, C-9), 106.9 (CH, C-10, C-14), 106.8 (CH, C-3, C-7), 101.9 (CH, C-12), 101.6 (CH, C-5), 78.4 (C, C-16), 75.9 (CH, C-17), 72.1 (CH_2, C-1), 69.9 (CH_2, C-8), 56.0 (CH_2, C-15).

j) Synthesis of 10b:

tris(2-((3,5-bis(prop-2-yn-1-yloxy)benzyl)oxy)ethyl)amine

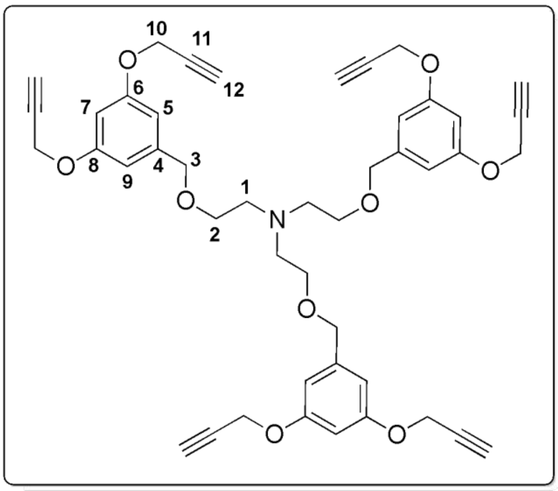

NaH 60% dispersion (20 mg, 0.500 mmol, 1.25 equiv) was added to a solution of triethanolamine (53 µL, 0.400 mmol, 1.0 equiv) in dry DMF (5.0 mL) under argon at room temperature and the resulting mixture was stirred for 1 h. Then, 1-(bromomethyl)-3,5-bis(prop-2-yn-1-yloxy)benzene[1] (112 mg, 0.400 mmol, 1.0 equiv) was added and the reaction was stirred for 2 h at the same temperature. This procedure was repeated twice until a total of 3.75 equiv of NaH and 3.0 equiv of **5** were added. DMF was removed under reduced pressure and the resulting crude was purified by flash chromatography (hexanes/AcOEt 1:5) affording hexaalkyne **10b** as a colorless oil, (181 mg, 61%).

^{1}H-NMR ($CDCl_3$, 250 MHz): **δ** = 6.57 (d, J = 2.3 Hz, 6H, H-5, H-9), 6.51 (t, J = 2.3 Hz, 3H, H-7), 4.64 (d, J = 2.4 Hz, 12H, H-10), 4.44 (s, 6H, H-3), 3.56 (t, J = 5.9 Hz, 4H, H-2), 2.86 (t, J = 5.9 Hz, 4H, H-1), 2.52 (t, J = 2.4 Hz, 6H, H-12).

^{13}C-NMR ($CDCl_3$, 62.5 MHz): **δ** = 158.8 (C, C-6, C-8), 141.2 (C, C-4), 106.9 (CH, C-5, C-9), 101.5 (CH, C-7), 78.5 (C, C-11), 75.8 (CH, C-12), 72.9 (CH_2, C-3), 68.9 (CH_2, C-2), 55.9 (CH_2, C-10), 54.8 (CH_2, C-1).

k) Synthesis of 10c

5,5'-(((2,2-bis(((3,5-bis(prop-2-yn-1-yloxy)benzyl)oxy)methyl)propane-1,3-diyl)bis (oxy))bis(methylene))bis(1,3-bis(prop-2-yn-1-yloxy)benzene)

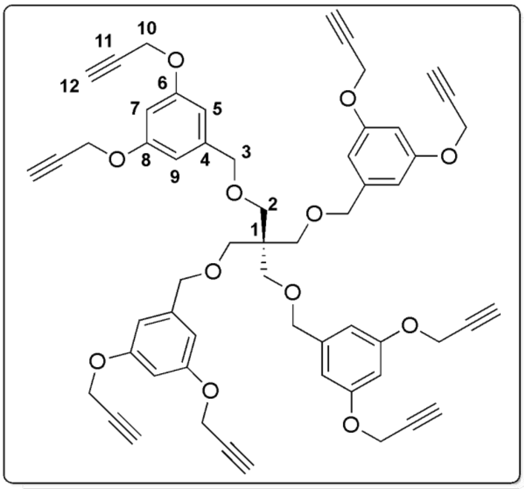

NaH 60% dispersion (24 mg, 0.595 mmol, 1.0 equiv) was added to a solution of pentaerythritol (81 mg, 0.595 mmol, 1.0 equiv) in dry DMF (10.0 mL) under argon and the resulting mixture was stirred at room temperature for 30 min. Then, 1-(bromomethyl)-3,5-bis(prop-2-yn-1-yloxy)benzene[1] (166 mg, 0.595 mmol, 1.0 equiv) was added and the reaction was stirred for 1 h at the same temperature. This procedure was repeated three times, until a total of 4.0 equiv of NaH and 4.0 equiv of 1-(bromomethyl)-3,5-bis(prop-2-yn-1-yloxy)benzene[1] were added. After removing DMF under reduced pressure AcOEt (25 mL) was added and the resulting mixture was washed with brine (2 x 20 mL) and distilled water (2 x 20 mL), dried over anhydrous Na_2SO_4, filtered and concentrated under vacuum. The crude was purified by flash chromatography (hexanes/AcOEt 1:2) affording octaalkyne **10c** as a white solid (641 mg, 69%).

^{1}H-NMR ($CDCl_3$, 250 MHz): **δ** = 6.54 (d, *J* = 2.2 Hz, 8H, H-5, H-9), 6.51 (t, *J* = 2.2 Hz, 3H, H-7), 4.63 (d, *J* = 2.4 Hz, 16H, H-10), 4.44 (s, 8H, H-3), 3.57 (s, 8H, H-2), 2.51 (t, *J* = 2.4 Hz, 8H, H-12).
^{13}C-NMR ($CDCl_3$, 62.5 MHz): **δ** = 158.8 (C, C-6, 8), 141.2 (C, C-4), 106.8 (CH, C-5, 9), 101.4 (CH, C-7), 78.5 (C, C-11), 75.9 (CH, C-12), 73.3 (CH_2, C-3), 68.9 (CH_2, C-2), 71.0 (CH_2, C-2), 56.0 (CH_2, C-10), 45.2 (C, C-1).

2. Glucuronic Acid Glycodendrimers Synthesis and Characterization.

a) Glycodendrimer 3a

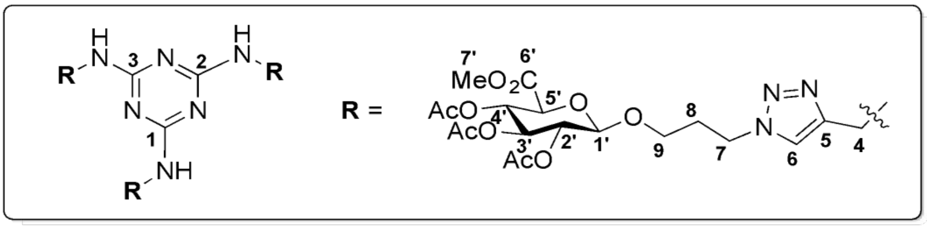

Following the general procedure A a mixture of azide **2** (319.1 mg, 0.765 mmol, 4.5 equiv), 2,4,6-tri(prop-2-yn-1-yl)-1,3,5-triazine[5] (40.4 mg, 0.168 mmol, 1.0 equiv), sodium (*L*)-ascorbate (34.9 mg, 0.176 mmol, 1.05 equiv) and $CuSO_4{\cdot}5H_2O$ (31.6 mg, 0.126 mmol, 0.75 equiv) in DMF/H_2O 98:2 (2 mL) was irradiated at 65 ºC for 90 min. The resulting residue was purified (SiO_2, hexanes/AcOEt 1:2 to DCM/MeOH 10:1) to yield **3a** as a white foam solid (180.5 mg, 72%).

^{1}H-NMR ($CDCl_3$, 500 MHz): **δ** = 7.73 (br s, 3H, H-6), 5.27 (t, *J* = 9.5 Hz, 3H, H-3'), 5.19 (t, *J* = 9.5 Hz, 3H, H-4'), 5.01 (dd, *J* = 9.5, 7.7 Hz, 3H, H-2'), 4.67 (br s, 6H, H-4), 4.56 (d, *J* = 7.7 Hz, 3H, H-1'), 4.47-4.39 (m, 3H, H-7a), 4.39-4.31 (m, 3H, H-7b), 4.05 (d, *J* = 9.5 Hz, 3H, H-5'), 3.87-3.79 (m, 3H, H-9a), 3.73 (s, 9H, H-7'), 3.51-3.44 (m, 3H, H-9b), 2.22-2.08 (m, 6H, H-8), 2.06 (s, 9H, OAc), 2.02 (s, 9H, OAc), 2.01 (s, 9H, OAc).

^{13}C-NMR ($CDCl_3$, 125 MHz): **δ** = 170.2 (CH_3*C*O), 169.6 (CH_3*C*O), 169.6 (CH_3*C*O), 167.5 (*C*O_2CH_3, C-6'), 166.0 (C_{Ar}, C-1, C-2, C-3), 146.1 ($C_{Triazole}$, C-5), 123.0 ($CH_{Triazole}$, C-6), 100.7 (CH, C-1'), 72.4 (CH, C-5'), 72.1 (CH, C-3'), 71.3 (CH, C-2'), 69.5 (CH, C-4'), 66.1 (CH_2, C-9), 53.1 (OCH_3, C-7'), 46.6 (CH_2, C-7), 36.2 (CH_2, C-4), 30.2 (CH_2, C-8), 20.8 (*C*H_3CO), 20.7 (*C*H_3CO), 20.6 (*C*H_3CO).

m/z calcd for $C_{60}H_{81}N_{15}O_{30}H_2$ [M+2H]$^{2+}$ 746.7710, **found MS(ESI)**: **[M+2H]$^{2+}$** = 746.7732

b) Glycodendrimer 3b

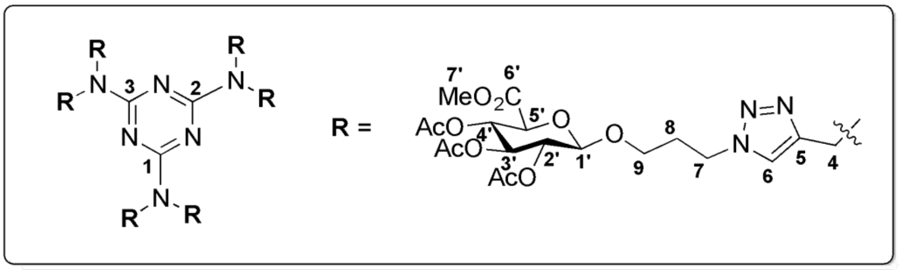

Following the general procedure A a mixture of azide **2** (275.0 mg, 0.659 mmol, 9.0 equiv), alkyne **1b** (26.0 mg, 0.073 mmol, 1.0 equiv), sodium (*L*)-ascorbate (30.4 mg, 0.153 mmol, 2.1 equiv) and $CuSO_4{\cdot}5H_2O$ (27.3 mg, 0.109 mmol, 1.5 equiv) in DMF/H_2O 98:2 (2 mL) was irradiated at 65 ºC for 120 min. The resulting residue was purified (SiO_2, hexanes/AcOEt 1:2 to DCM/MeOH 10:2) to yield **3b** as a white foam solid (189.9 mg, 91%).

^{1}H-NMR ($CDCl_3$, 250 MHz): **δ** = 8.09 (br s, 6H, H-6), 5.28 (t, *J* = 9.5 Hz, 6H, H-3'), 5.17 (t, *J* = 9.5 Hz, 6H, H-4'), 5.00 (dd, *J* = 9.5, 7.7 Hz, 6H, H-2'), 4.90, (br s, 12H, H-4), 4.61 (d, *J* = 7.7 Hz, 6H, H-1'), 4.48-4.31 (m, 12H, H-7), 4.08 (d, *J* = 9.5 Hz, 6H, H-5'), 3.96-3.82 (m, 6H, H-9a), 3.71 (s, 18H, H-7'), 3.55-3.41 (m, 6H, H-9b), 2.21-2.08 (m, 12H, H-8), 2.06 (s, 18H, OAc), 2.01 (s, 36H, OAc).

^{13}C-NMR ($CDCl_3$, 62.5 MHz): **δ** = 170.2 (CH_3*C*O), 169.6 (CH_3*C*O), 169.5 (CH_3*C*O), 167.4 (*C*O_2CH_3, C-6'), 164.6 (C_{Ar}, C-1, C-2, C-3), 144.3 ($C_{Triazole}$, C-5), 124.5 (C $CH_{Triazole}$, C-6), 100.7 (CH, C-1'), 72.4 (CH, C-5'), 72.1 (CH, C-3'), 71.3 (CH, C-2'), 69.5 (CH, C-4'), 66.4 (CH_2, C-9), 53.0 (OCH_3, C-7'), 46.9 (CH_2, C-7), 40.9 (CH_2, C-4), 30.3 (CH_2, C-8), 20.9 (*C*H_3CO), 20.7 (*C*H_3CO), 20.6 (*C*H_3CO).
m/z calcd for $C_{117}H_{156}N_{24}O_{60}H$ [M+H]$^{+}$ 2857.9966, **found MS(MALDI-TOF)**: **[M+H]$^{+}$** = 2858.0103

c) Glycodendrimer 3c

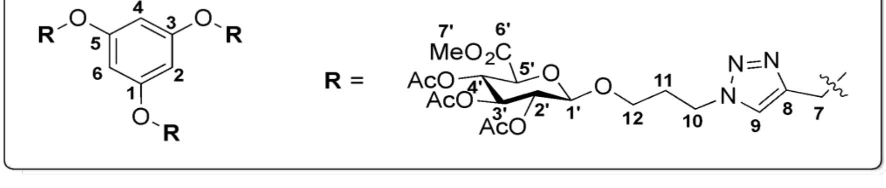

Following the general procedure A a mixture of azide **2** (394.2 mg, 0.945 mmol, 4.5 equiv), alkyne **1c** (50.9 mg, 0.212 mmol, 1.0 equiv), sodium (*L*)-ascorbate (44.2 mg, 223 mmol, 1.05 equiv) and $CuSO_4{\cdot}5H_2O$ (39.7 mg, 0.159 mmol, 0.75 equiv) in DMF/H_2O 98:2 (2 mL) was irradiated at 65 ºC for 90 min. The resulting residue was purified (SiO_2,

hexanes/AcOEt 1:2 to DCM/MeOH 10:1) to yield **3c** as a white foam solid (237 mg, 75%).

^{1}H-NMR ($CDCl_3$, 500 MHz): **δ** = 7.77 (br s, 3H, H-9), 6.27 (s, 3H, H-2, H-4, H-6), 5.25 (t, *J* = 9.6 Hz, 3H, H-3'), 5.18 (t, *J* = 9.6 Hz, 3H, H-4'), 5.12 (br s, 6H, H-7), 5.01 (dd, *J* = 9.6, 7.7 Hz, 3H, H-2'), 4.55 (d, *J* = 7.7 Hz, 3H, H-1'), 4.52-4.46 (m, 3H, H-10a), 4.44-4.38 (m, 3H, H-10b), 4.02 (d, *J* = 9.6 Hz, 3H, H-5'), 3.85-3.79 (m, 3H, H-12a), 3.75 (s, 9H, H-7'), 3.55-3.49 (m, 3H, H-12b), 2.21-2.11 (m, 6H, H-11), 2.06 (s, 9H, OAc), 2.01 (s, 18H, OAc).

^{13}C-NMR ($CDCl_3$, 125 MHz): **δ** = 170.1 ($CH_3\mathit{C}O$), 169.6 ($CH_3\mathit{C}O$), 169.5 ($CH_3\mathit{C}O$), 167.4 (CO_2CH_3, C-6'), 160.2 (C_{Ar}, C-1, C-3, C-5), 143.7 ($C_{Triazole}$, C-8), 124.1 ($CH_{Triazole}$, C-9), 100.6 (CH, C-1'), 95.2 (C_{Ar}, C-2, C-4, C-6), 72.4 (CH, C-5'), 72.0 (CH, C-3'), 71.3 (CH, C-2'), 69.5 (CH, C-4'), 65.9 (CH_2, C-12), 61.9 (CH_2, C-7), 53.0 (OCH_3, C-7'), 46.7 (CH_2, C-10), 30.2 (CH_2, C-11), 20.8 ($\mathit{C}H_3CO$), 20.7 ($\mathit{C}H_3CO$), 20.6 ($\mathit{C}H_3CO$).
m/z calcd for $C_{63}H_{81}N_9O_{33}H$ [M+H]$^+$ 1492.5010, **found MS(MALDI-TOF)**: **[M+H]$^+$** = 1492.5016

d) Glycodendrimer 3d

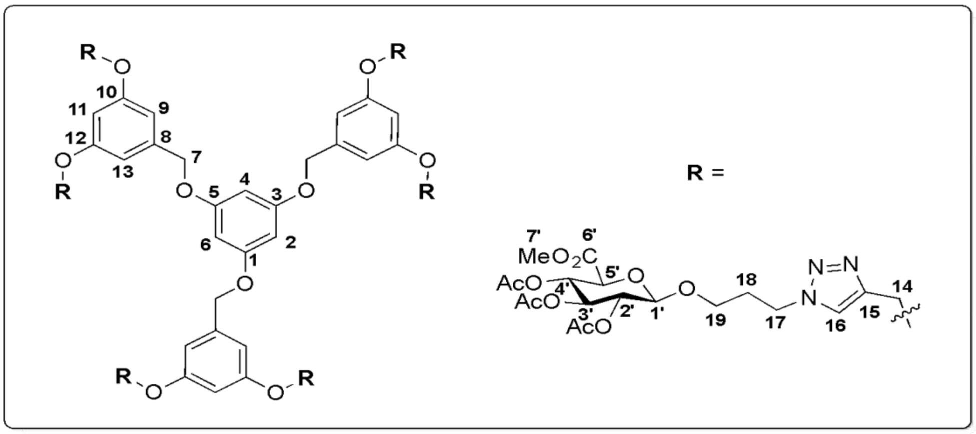

Following the general procedure A a mixture of azide **2** (232.8 mg, 0.630 mmol, 10.2 equiv), alkyne **1d** (44.7 mg, 0.062 mmol, 1.0 equiv), sodium (*L*)-ascorbate (26 mg, 0.131 mmol, 2.1 equiv) and $CuSO_4{\cdot}5H_2O$ (23.3 mg, 0.093 mmol, 1.5 equiv) in DMF/H_2O 98:2 (2 mL) was irradiated at 65 ºC for 120 min. The resulting residue was purified (SiO_2, hexanes/AcOEt 1:2 to DCM/MeOH 10:1) to yield **3d** as a white foam solid (158.0 mg, 79%).
^{1}H-NMR ($CDCl_3$, 250 MHz): **δ** = 7.82 (br s, 6H, H-16), 6.67 (d, *J* = 2.2 Hz, 6H, H-9, H-13), 6.60 (t, *J* = 2.2 Hz, 3H, H-11), 6.21 (s, 3H, H-2, H-4, H-6), 5.26 (t, *J* = 9.6 Hz, 6H, H-3'), 5.18 (t, *J* = 9.6 Hz, 6H, H-4'), 5.17 (br s, 12H, H-14), 5.01 (dd, *J* = 9.6, 7.7 Hz, 6H, H-2'), 4.94 (s, 6H, H-7), 4.64-4.46 (m, 6H, H-17a), 4.55 (d, *J* = 7.7 Hz, 6H, H-1'), 4.46-4.33 (m, 6H, H-17b), 4.02 (d, *J* = 9.6 Hz, 6H, H-5'), 3.88-3.79 (m, 6H, H-19a), 3.68 (s, 18H, H-7'), 3.56-3.47 (m, 6H, H-19b), 2.21-2.12 (m, 12H, H-18), 2.06 (s, 18H, OAc), 2.01 (s, 18H, OAc), 2.01 (s, 18H, OAc).

^{13}C-NMR ($CDCl_3$, 62.5 MHz): **δ** = 170.1 ($CH_3\mathit{C}O$), 169.6 ($CH_3\mathit{C}O$), 169.6 ($CH_3\mathit{C}O$), 167.4 ($\mathit{C}O_2CH_3$, C-6'), 160.6 (C_{Ar}, C-1, C-3, C-5), 159.7 (C_{Ar}, C-10, C-12), 143.5 ($C_{Triazole}$, C-15), 139.6 (C_{Ar}, C-8), 124.2 ($CH_{Triazole}$, C-16), 106.6 (C_{Ar}, C-9, C-13), 101.0

(C_{Ar}, C-11), 100.6 (CH, C-1'), 95.1 (CH_{Ar}, C-2, C-4, C-6), 72.4 (CH, C-5'), 72.0 (CH, C-3'), 71.3 (CH, C-2'), 71.3 (CH_2, C-7), 69.5 (CH, C-4'), 65.9 (CH_2, C-19), 61.9 (CH_2, C-14), 53.1 (OCH_3, C-7'), 46.9 (CH_2, C-17), 30.2 (CH_2, C-18), 20.9 (*C*H_3CO), 20.7 (*C*H_3CO), 20.6 (*C*H_3CO).
m/z calcd for $C_{141}H_{174}N_{18}O_{69}H$ [M+H]$^+$ 3224.0733, **found MS(MALDI-TOF)**: **[M+H]$^+$** = 3224.0741

e) Glycodendrimer 3e

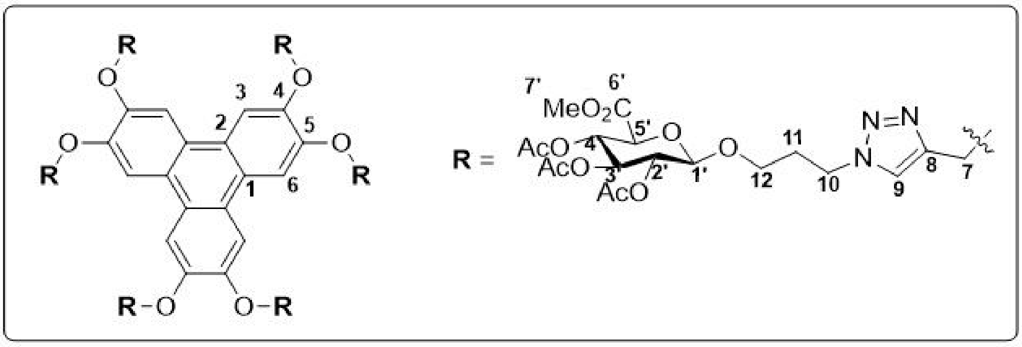

Following the general procedure B a mixture of azide **2** (166.6 mg, 0.339 mmol, 7.5 equiv), alkyne **1e** (25.0 mg, 0.0452 mmol, 1.0 equiv), sodium (*L*)-ascorbate (21.5 mg, 0.109 mmol, 2.4 equiv) and $CuSO_4·5H_2O$ (13.6 mg, 0.054 mmol, 1.2 equiv) in DMSO/H_2O 5:1 (3 mL) was irradiated at 65 ºC for 90 min. The resulting residue was purified (SiO_2, AcOEt to AcOEt/MeOH 20:1) to yield **3e** as a brown foam solid (83 mg, 60%).

^{1}H-NMR ($CDCl_3$, 250 MHz): **δ** = 8.10 (br s, 6H, H-9), 7.89 (s, 6H, H-3, H-6), 5.50 (s, 12H, H-7), 5.21-5.07 (m, 12H, H-3', H-4'), 4.95 (t, *J* = 8.4 Hz, 6H, H-2'), 4.56-4.37 (m, 12H, H-10), 4.31 (d, *J* = 7.7 Hz, 6H, H-1'), 3.82 (d, *J* = 9.4 Hz, 6H, H-5'), 3.77-3.73 (m, 6H, H-12a), 3.67 (s, 18H, H-7'), 3.42-3.34 (m, 6H, H-12b), 2.22-2.15 (m, 12H, H-11). 2.04 (s, 18H, OAc), 2.02 (s, 18H, OAc), 2.00 (s, 18H, OAc).

^{13}C-NMR ($CDCl_3$, 62.5 MHz): **δ** = 170.4 (CH_3*C*O), 169.9 (CH_3*C*O), 169.8 (CH_3*C*O), 167.6 (CO_2CH_3, C-6'), 148,5 (C_{Ar}, C-4, C-5), 144.1 ($C_{Triazole}$, C-8), 124,7 ($CH_{Triazole}$, C-9), 123,9 (C_{Ar}, C-1, C-2), 108,4 (CH_{Ar}, C-3, C-6), 100.7 (CH, C-1'), 72.5 (CH, C-5’), 72.2 (CH, C-3’), 71.5 (CH, C-2’), 69.7 (CH, C-4’), 66.2 (CH_2, C-12), 63.4 (CH_2, C-7), 53.3 (OCH_3, C-7'), 47.0 (CH_2, C-10), 30.5 (CH_2, C-11), 21.1 (*C*H_3CO), 21.0 (*C*H_3CO), 20.9 (*C*H_3CO).

m/z calcd for $C_{132}H_{162}N_{18}O_{66}H$ [M+H]$^+$ 3055.9946**, found MS(MALDI-TOF)**: **[M+H]$^+$** = 3056.0012

f) Glycodendrimer 3f

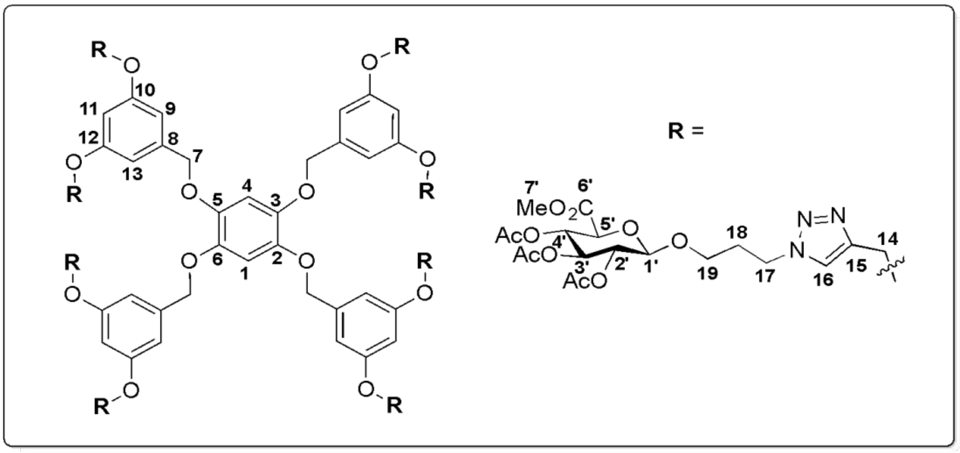

Following the general procedure A a mixture of azide **2** (234.3 mg, 0.562 mmol, 12.0 equiv), alkyne **1f** (43.8 mg, 0.047 mmol, 1.0 equiv), sodium (*L*)-ascorbate (26.4 mg, 0.133 mmol, 2.8 equiv) and $CuSO_4·5H_2O$ (23.6 mg, 0.095 mmol, 2.0 equiv) in DMF/H_2O 98:2 (2 mL) was irradiated at 65 ºC for 180 min. The resulting residue was purified (SiO_2, hexanes/AcOEt 1:2 to DCM/MeOH 20:1) to yield **3f** as a white foam solid (152.8 mg, 76%).

^{1}H-NMR ($CDCl_3$, 250 MHz): **δ** = 7.75 (br s, 8H, H-16), 6.62 (d, *J* = 2.2 Hz, 8H, H-9, H-13), 6.49 (t, *J* = 2.2 Hz, 4H, H-11), 6.41 (s, 2H, H-1, H-4), 5.27-5.09 (m, 16H, H-3', H-4'), 5.14 (s, 16H, H-14), 5.00 (dd, *J* = 9.6, 7.7 Hz, 8H, H-2'), 4.64-4.31 (m, 16H, H-17), 4.61 (s, 8H, H-7), 4.50 (d, *J* = 7.7 Hz, 8H, H-1'), 4.00 (d, *J* = 9.6 Hz, 8H, H-5'), 3.88-3.79 (m, 13H, H-19a), 3.68 (s, 30H, H-7'), 3.56-3.47 (m, 14H, H-19b), 2.18-2.09 (m, 18H, H-18), 2.05 (s, 24H, OAc), 2.00 (s, 24H, OAc), 1.99 (s, 24H, OAc).

^{13}C-NMR ($CDCl_3$, 62.5 MHz): **δ** = 170.2 (CH_3*C*O), 169.6 (CH_3*C*O), 169.5 (CH_3*C*O), 167.3 (*C*O_2CH_3, C-6'), 159.6 (C_{Ar}, C-10, C-12), 144.1 (C_{Ar}, C-2, C-3, C-5, C-6), 143.7 ($C_{Triazole}$, C-15), 137.6 (C_{Ar}, C-8), 124.0 ($CH_{Triazole}$, C-16), 105.9 (C_{Ar}, C-9, C-13), 105.7 (C_{Ar}, C-1, C-4), 101.1 (C_{Ar}, C-11), 100.6 (CH, C-1'), 72.3 (CH, C-5'), 72.0 (CH, C-3'), 71.2 (CH, C-2'), 69.4 (CH, C-4'), 65.9 (CH_2, C-19), 64.9 (CH_2, C-7), 61.9 (CH_2, C-14), 53.1 (OCH_3, C-7'), 46.7 (CH_2, C-17), 30.1 (CH_2, C-18), 20.9 (*C*H_3CO), 20.7 (*C*H_3CO), 20.6 (*C*H_3CO).

m/z calcd for $C_{186}H_{230}N_{24}O_{92}H$ [M+H]$^+$ 4272.4130, **found MS(MALDI-TOF)**: **[M+H]$^+$** = 4272.3988

g) Glycodendrimer 8a

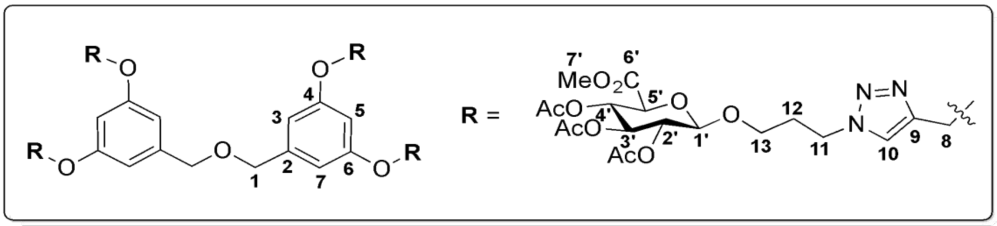

Following the general procedure B a mixture of azide **2** (231.5 mg, 0.555 mmol, 4.6 equiv), alkyne **7a** (50.0 mg, 0.121 mmol, 1.0 equiv), sodium (*L*)-ascorbate (38.2 mg, 0.193 mmol, 1.6 equiv) and $CuSO_4·5H_2O$ (24.1 mg, 0.096 mmol, 0.8 equiv) in DMSO/H_2O 5:1 (3 mL) was irradiated at 65 ºC for 90 min. The resulting residue was

purified (SiO_2, AcOEt to AcOEt/MeOH 20:1) to yield **8a** as a white foam solid (200.2 mg, 80%).

^{1}H NMR ($CDCl_3$, 250 MHz) **δ** = 7.78 (s, 4H, H-10), 6.62 (d, *J* = 2.2 Hz, 4H, H-3, H-7), 6.56 (t, *J* = 2.3 Hz, 2H, H-5), 5.33-4.94 (m, 8H, H-3’, H-4’), 5.15 (s, 8H, H-8), 5.01 (dd, *J* = 8.8, 7.9 Hz 4H, H-2’), 4.54 (d, *J* = 7.7 Hz, 4H, H-1’), 4.50-4.33 (m, 8H, H-11), 4.46 (s, 4H, H-1), 4.02 (d, *J* = 9.4 Hz, 4H, H-5’), 3.90-3.77 (m, 4H, H-13a), 3.68 (s, 12H, H-7’), 3.60-3.42 (m, 4H, H-13b), 2.24-2.13 (m, 8H, H-12), 2.06 (s, 12H, OAc), 2.01 (s, 24H, OAc).

^{13}C NMR ($CDCl_3$, 62.5 MHz): **δ** = 170.2 (CH_3*C*O), 169.6 (CH_3*C*O), 169.5 (CH_3*C*O), 167.4 (*C*O_2CH_3, C-6'), 159.7 (C_{Ar}, C-4, 6), 143.8 (C_{Ar}, C-2), 140.9 ($C_{Triazole}$ C-9), 124.0 ($CH_{Triazole}$, C-10), 106.9 (C_{Ar}, C-3, 7), 101.4 (C_{Ar}, C-5), 100.6 (CH, C-1'), 72.4 (CH, C-5’), 72.0 (CH, C-3’), 71.3 (CH, C-2’), 69.5 (CH, C-4'), 66.0 (CH_2, C-13), 62.0 (CH_2, C-1), 60.6 (CH_2, C-8), 53.1 (O*C*H_3, C-7'), 46.7 (CH_2, C-11), 30.3 (CH_2, C-12), 20.9 (*C*H_3-CO), 20.8 (*C*H_3CO), 20.6 (*C*H_3CO).

m/z calcd for $C_{90}H_{114}N_{12}O_{45}H$ [M+H]$^+$ 2083.7074**, found MS(MALDI-TOF)**: **[M+H]$^+$** = 2083.6981

h) Glycodendrimer 8b

i)

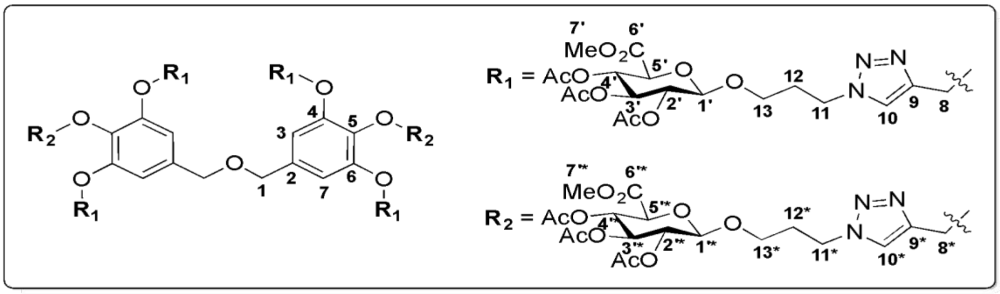

Following the general procedure a mixture of azide **2** (207.2 mg, 0.491 mmol, 6.9 equiv), alkyne **7b** (37.6 mg, 0.072 mmol, 1.0 equiv), sodium (*L*)-ascorbate (34.3 mg, 0.173 mmol, 2.4 equiv) and $CuSO_4 \cdot 5H_2O$ (21.6 mg, 0.086 mmol, 1.2 equiv) in DMSO/H_2O 5:1 (3 mL) was irradiated at 65 ºC for 90 min. The resulting residue was purified (SiO_2, AcOEt to AcOEt/MeOH 20:1) to yield **8b** as a white foam solid (138.5 mg, 64%).

^{1}H NMR ($CDCl_3$, 250 MHz): **δ** = 7.95 (s, 4H, H-10), 7.88 (s, 2H, H-10*), 6.79 (s, 4H, H-3, H-7), 5.33-5.06 (m, 24H, H-3’, H-3’*, H-4’, H-4’*, H-8, H-8*), 5.00 (dd, *J* = 9.3, 7.6 Hz, 6H, H-2’, H-2’*), 4.60 (d, *J* = 7.8 Hz, 4H, H-1’), 4.58 (d, *J* = 7.7 Hz, 2H, H-1’*), 4.54-4.30 (m, 16H, H-1, H-11, H-11*), 4.06 (d, *J* = 9.7 Hz, 2H, H-5’*), 4.05 (d, *J* = 9.5 Hz, 4H, H-5’), 3.93-3.79 (m, 6H, H-13a, H-13a*), 3.69 (s, 6H, H-7’*), 3.67 (s, 12H, H-7’), 3.60-3.40 (m, 6H, H-13, H-13b*), 2.22-2.10 (m, 12H, H-12, H-12*), 2.05 (s, 18H, OAc), 2.00 (s, 36H, OAc). (164H)

^{13}C NMR ($CDCl_3$, 62.5 MHz): **δ** = 170.1 (CH_3*C*O), 169.6 (CH_3*C*O), 169.6 (CH_3*C*O*), 167.4 (CH_3*C*O), 167.4 (CH_3*C*O*), 152.4 (*C*O_2CH_3, C-6', C-6’*), 144.7 (C, C-2), 143.8 ($C_{Triazole}$ C-9, C-9*), 137.4 (C, C-5), 134.4 (C, C-4, C-6), 124.4 ($CH_{Triazole}$, C-10), 124.3 ($CH_{Triazole}$, C-10*), 107.7 (C_{Ar}, C-3, C-7), 100.6 (CH, C-1'), 100.5 (CH, C-1'*), 72.3 (CH, C-5’, C-5’*), 72.0 (CH, C-3', C-3’*), 71.3 (CH, C-2’, C-2’*), 69.5 (CH, C-4', C-4’*), 66.4 (CH_2, C-13, C-13*), 66.1 (CH_2, C-1), 63.2 (CH_2, C-8, C-8*), 53.0 (OCH_3, C-7'), 53.0

(OCH$_3$, C-7'*), 46.7 (CH$_2$, C-11, C-11*), 30.2 (CH$_2$, -12, C-12*), 20.9 (*C*H$_3$CO), 20.7 (*C*H$_3$CO), 20.6 (*C*H$_3$CO).
m/z calcd for $C_{128}H_{164}N_{18}O_{67}H$ [M+H]$^+$ 3026.0052, **found MS(MALDI-TOF)**: **[M+H]$^+$** = 3026.0104

j) Glycodendrimer 8c

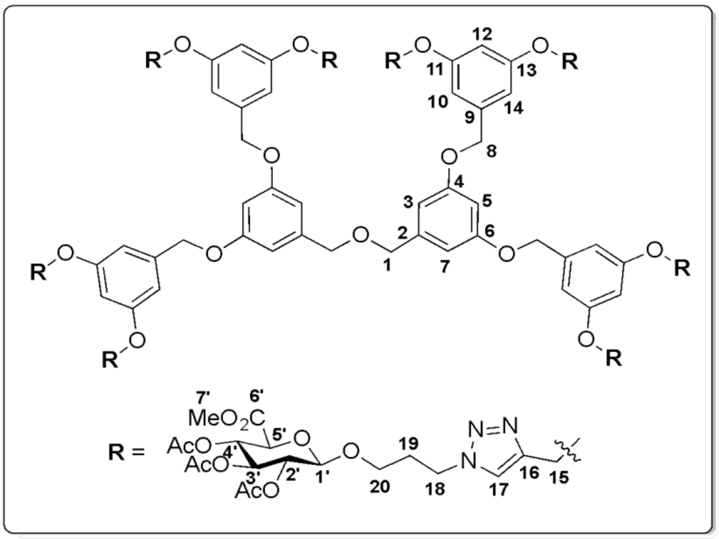

Following the general procedure B a mixture of azide **2** (169.6 mg, 0.406 mmol, 9.2 equiv), alkyne **7c** (46.6 mg, 0.044 mmol, 1.0 equiv), sodium (*L*)-ascorbate (28.1 mg, 0.141 mmol, 3.2 equiv) and $CuSO_4 \cdot 5H_2O$ (17.7 mg, 0.070 mmol, 1.6 equiv) in DMSO/H_2O 5:1 (3 mL) was irradiated at 65 ºC for 90 min. The resulting residue was purified (SiO_2, AcOEt to AcOEt/MeOH 10:1) to yield **8c** as a white foam solid (159.0 mg, 85%).

^{1}H NMR (CDCl$_3$, 250 MHz)**: δ** = 7.77 (s, 8H, H-17), 6.66 (d, *J* = 2.1 Hz, 8H, H-10, H-14), 6.58 (m, 8H, H-3, H-7, H-12), 6.49 (t, *J* = 2.2 Hz, 2H, H-5), 5.25-5.15 (m, 16H, H-3', H-4'), 5.13 (s, 16H, H-15), 5.00 (dd, *J* = 9.2, 7.8 Hz, 8H, H-2'), 4.95 (s, 8H, H-8), 4.53 (d, *J* = 7.8 Hz, 8H, H-1'), 4.48 (s, 4H, H-1) 4.47-4.32 (m, 16H, H-18), 4.01 (d, *J* = 9.4 Hz, 8H, H-5'), 3.88-3.77 (m, 8H, H-20a), 3.67 (s, 24H, H-7'), 3.56-3.42 (m, 8H, H-20b), 2.18-2.09 (m, 16H, H-19), 2.05 (s, 24H, OAc), 2.00 (s, 24H, OAc), 1.99 (s, 24H, OAc).

^{13}C NMR (D$_2$O, 62.5 MHz): **δ** = 170.1 (CH$_3$*C*O), 169.6 (CH$_3$*C*O), 169.5 (CH$_3$*C*O), 167.3 (*C*O$_2$CH$_3$, C-6'), 160.0 (C$_{Ar}$, C-4, C-6), 159.7 (C$_{Ar}$, C-11, C-13), 143.6 (C$_{Triazole}$, C-16), 140.9 (C$_{Ar}$, C-2), 139.5 (C$_{Ar}$, C-9), 124.0 (CH$_{Triazole}$, C-17), 106.7 (C$_{Ar}$, C-3, C-7), 106.6 (C$_{Ar}$, C-10, C-14), 101.5 (C$_{Ar}$, C-3, C-7), 101.4 (C$_{Ar}$, C-12), 100.6 (CH, C-1'), 72.3 (CH, C-5'), 72.2, (CH$_2$, C-1), 72.0 (CH, C-3'), 71.2 (CH, C-2'), 69.9 (CH$_2$, C-8), 69.5 (CH, C-4'), 65.9 (CH$_2$, C-20), 61.9 (CH$_2$, C-15), 53.0 (O*C*H$_3$, C-7'), 46.7 (CH$_2$, C-18) 30.2 (CH$_2$, C-19), 20.8 (*C*H$_3$CO), 20.7 (*C*H$_3$CO), 20.6 (*C*H$_3$CO).

m/z calcd for $C_{194}H_{238}N_{24}O_{93}H$ [M+H]$^+$ 4392.4705, **found MS(MALDI-TOF)**: **[M+H]$^+$** = 4392.4666

k) Glycodendrimer 11a

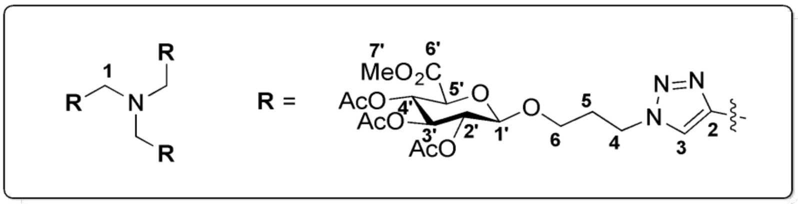

Following the general procedure A a mixture of azide **2** (407.4 mg, 0.976 mmol, 4.5 equiv), alkyne **10a** (28.5 mg, 0.217 mmol, 1.0 equiv), sodium (*L*)-ascorbate (45.2 mg, 0.228 mmol, 1.05 equiv) and $CuSO_4{\cdot}5H_2O$ (40.6 mg, 0.163 mmol, 0.75 equiv) in DMF/H_2O 98:2 (2 mL) was irradiated at 65 ºC for 90 min. The resulting residue was purified (SiO_2, hexanes/AcOEt 1:2 to DCM/MeOH 10:2) to yield **11a** as a white foam solid (150.1 mg, 51%).

^{1}H-NMR ($CDCl_3$, 250 MHz): **δ** = 7.79 (br s, 3H, H-3), 5.30 (t, *J* = 9.5 Hz, 3H, H-3'), 5.19 (t, *J* = 9.5 Hz, 3H, H-4'), 5.03 (dd, *J* = 9.5, 7.7 Hz, 3H, H-2'), 4.66 (d, *J* = 7.7 Hz, 3H, H-1'), 4.44 (br s, 6H, H-1), 4.08 (d, *J* = 9.5 Hz, 3H, H-5'), 3.95-3.86 (m, 3H, H-6a), 3.79-3.73 (m, 3H, H-1), 3.72 (s, 9H, H-7'), 3.56-3.45 (m, 3H, H-6b), 2.26-2.12 (m, 6H, H-5), 2.07 (s, 9H, OAc), 2.01 (s, 18H, OAc).

^{13}C-NMR ($CDCl_3$, 62.5 MHz): **δ** = 170.2 (CH_3*C*O), 169.6 (CH_3*C*O), 169.5 (CH_3*C*O), 167.4 (*C*O_2CH_3, C-6'), 144.1 ($C_{Triazole}$, C-2), 130.9 ($CH_{Triazole}$, C-3), 100.7 (CH, C-1'), 72.5 (CH, C-5'), 72.1 (CH, C-3'), 71.3 (CH, C-2'), 69.6 (CH, C-4'), 66.2 (CH_2, C-6), 53.1 (OCH_3, C-7'), 46.7 (CH_2, C-4), 46.6 (CH_2, C-1), 30.3 (CH_2, C-5), 20.9 (*C*H_3CO), 20.7 (*C*H_3CO), 20.6 (*C*H_3CO).
m/z calcd for $C_{57}H_{78}N_{10}O_{30}H_2$ $[M+2H]^{2+}$ 692.2515, **found MS(ESI)**: **$[M+2H]^{2+}$** = 692.2525

l) Glycodendrimer 11b

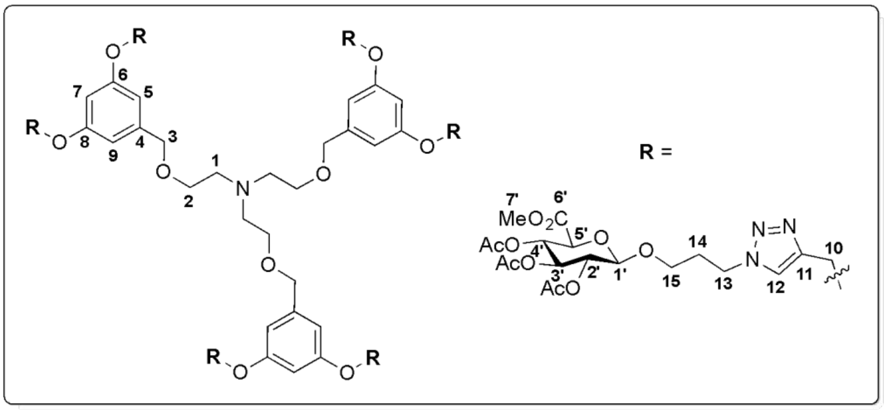

Following the general procedure A a mixture of azide **2** (231.0 mg, 0.561 mmol, 9.0 equiv), alkyne **10b** (46.0 mg, 0.062 mmol, 1.0 equiv), sodium (*L*)-ascorbate (25.7 mg, 0.130 mmol, 2.1 equiv) and $CuSO_4{\cdot}5H_2O$ (23.2 mg, 0.093 mmol, 1.5 equiv) in DMF/H_2O 98:2 (2 mL) was irradiated at 65 ºC for 120 min. The resulting residue was purified (SiO_2, hexanes/AcOEt 1:1 to DCM/MeOH 10:1) to yield **11b** as a white foam solid (103.0 mg, 69%).

^{1}H-NMR ($CDCl_3$, 250 MHz): **δ** = 7.73 (br s, 6H, H-12), 6.55 (d, *J* = 2.2 Hz, 6H, H-5, H-9), 6.51 (t, *J* = 2.2 Hz, 3H, H-7), 5.29-5.13 (m, 12H, H-3', H-4'), 5.10 (s, 12H, H-10), 4.98 (t, *J* = 8.3 Hz, 6H, H-2'), 4.52 (d, *J* = 7.7 Hz, 6H, H-1'), 4.47-4.30 (m, 12H, H-13), 4.40 (s, 6H, H-3), 4.00 (d, *J* = 9.4 Hz, 6H, H-5'), 3.86-3.74 (m, 6H, H-15a), 3.66 (s, 18H,

H-7'), 3.56-3.47 (m, 12H, H-15b, H-2), 2.86-2.76 (m, 6H, H-1), 2.18-2.07 (m, 12H, H-14), 2.03 (s, 18H, OAc), 1.98 (s, 36H, OAc).

^{13}C-NMR ($CDCl_3$, 62.5 MHz): **δ** = 170.0 ($CH_3\mathit{C}O$), 169.5 ($CH_3\mathit{C}O$), 169.4 ($CH_3\mathit{C}O$), 167.3 ($\mathit{C}O_2CH_3$, C-6'), 159.5 (C, C-6, C-8), 143.7 ($C_{Triazole}$, C-11), 141.1 (C, C-4), 123.9 ($CH_{Triazole}$, C-12), 106.5 (CH, C-5, C-9), 100.5 (CH, C-7, C-1'), 72.9 (CH_2, C-3), 72.3 (CH, C-5'), 71.9 (CH, C-3'), 71.2 (CH, C-2'), 69.4 (CH, C-4'), 65.9 (CH_2, C-15), 65.8 (CH_2, C-2), 61.8 (CH_2, C-10), 54.6 (CH_2, C-1), 52.9 (OCH_3, C-7'), 46.6 (CH_2, C-13), 30.1 (CH_2, C-14), 20.8 ($\mathit{C}H_3CO$), 20.6 ($\mathit{C}H_3CO$), 20.5 ($\mathit{C}H_3CO$).

m/z calcd for $C_{141}H_{183}N_{19}O_{69}H$ $[M+H]^+$ 3247.1468, **found MS(MALDI-TOF)**: **$[M+H]^+$** = 3247.1363

m) Glycodendrimer 11c

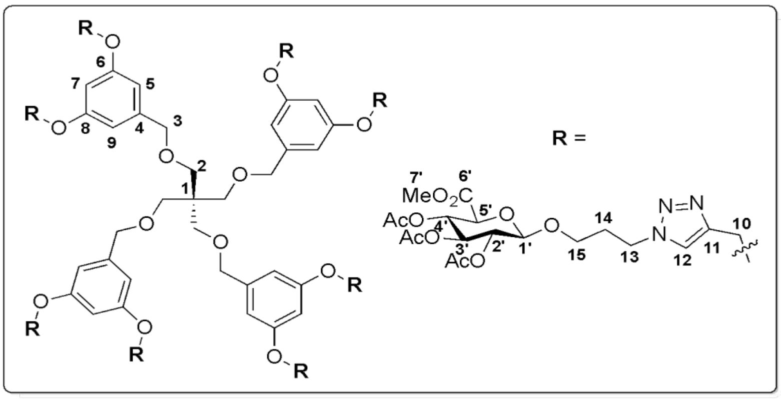

Following the general procedure A a mixture of azide **2** (289.0 mg, 0.693 mmol, 11.3 equiv), alkyne **10c** (57.0 mg, 0.061 mmol, 1.0 equiv), sodium (*L*)-ascorbate (32.1 mg, 0.162 mmol, 2.7 equiv) and $CuSO_4{\cdot}5H_2O$ (28.9 mg, 0.116 mmol, 1.90 equiv) in DMF/H_2O 98:2 (2 mL) was irradiated at 65 ºC for 180 min. The resulting residue was purified (SiO_2, hexanes/AcOEt 1:1 to DCM/MeOH 10:2) to yield **11c** as a white foam solid (151.6 mg, 79%).

^{1}H-NMR ($CDCl_3$, 250 MHz): **δ** = 7.77 (br s, 8H, H-12), 6.54 (d, *J* = 2.2 Hz, 6H, H-5, H-9), 6.52 (t, *J* = 2.2 Hz, 3H, H-7), 5.26 (t, *J* = 9.5 Hz, 8H, H-3'), 5.17 (t, *J* = 9.5 Hz, 8H, H-4'), 5.09 (s 16H, H-10), 5.02 (dd, *J* = 9.5, 7.7 Hz, 8H, H-2'), 4.56 (d, *J* = 7.7 Hz, 8H, H-1'), 4.50-4.34 (m, 16H, H-13), 4.42 (s, 8H, H-3), 4.03 (d, *J* = 9.4 Hz, 6H, H-5'), 3.88-3.78 (m, 8H, H-15a), 3.69 (s, 24H, H-7'), 3.59-3.46 (m, 16H, H-15b, H-2), 2.20-2.12 (m, 16H, H-14), 2.06 (s, 24H, OAc), 2.01 (s, 48H, OAc).

^{13}C-NMR ($CDCl_3$, 62.5 MHz): **δ** = 170.0 ($CH_3\mathit{C}O$), 169.5 ($CH_3\mathit{C}O$), 169.4 ($CH_3\mathit{C}O$), 167.3 ($\mathit{C}O_2CH_3$, C-6'), 159.5 (C, C-6, C-8), 143.6 ($C_{Triazole}$, C-11), 141.1 (C, C-4), 123.9 ($CH_{Triazole}$, C-12), 106.2 (CH, C-5, C-9), 100.5 (CH, C-1'), 100.4 (CH, C-7), 72.2 (CH, C-5'), 72.1 (CH_2, C-3), 71.9 (CH, C-3'), 71.1 (CH, C-2'), 69.4 (CH, C-4'), 65.9 (CH_2, C-15), 65.9 (CH_2, C-2), 61.7 (CH_2, C-10), 52.9 (OCH_3, C-7'), 46.6 (CH_2, C-13), 44.9 (C, C-1), 30.1 (CH_2, C-14), 20.7 ($\mathit{C}H_3CO$), 20.6 ($\mathit{C}H_3CO$), 20.5 ($\mathit{C}H_3CO$).

m/z calcd for $C_{185}H_{236}N_{24}O_{92}H$ $[M+H]^+$ 4266.4599, **found MS(MALDI-TOF)**: **$[M+H]^+$** = 4266.4608

3. Synthesis and characterization of compounds 4a-f, 9a-c and 12a-c.

a) Glycodendrimer 4a

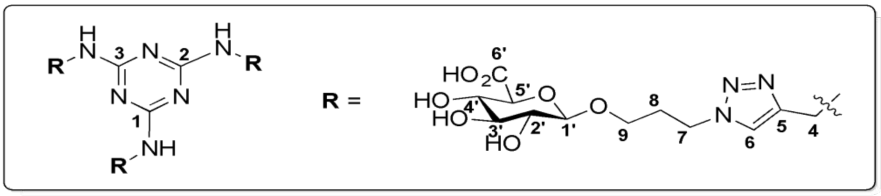

Following the general procedure a solution of protected trimer **3a** (100 mg, 0.0664 mmol) in 2 mL of anhydrous MeOH was treated sequentially with 0.5 N NaOMe/MeOH solution (13.5 equiv) and 0.2 M NaOH aq solution (4.5 equiv) to afford glycodendrimer **4a** in quantitative yields as a white powder.

^{1}H-NMR (D_2O, 700 MHz): **δ** = 7.75 (br s, 3H, H-9), 4.74 (s br, 6H, H-4), 4.61-4.52 (m, 6H, H-7), 4.40 (d, J = 8.4 Hz, 3H, H-1'), 3.89 (d, J = 9.5 Hz, 3H, H-5'), 3.75-3.66 (m, 3H, H-9a), 3.52 (t, J = 9.5 Hz, 3H, H-4'), 3.48 (t, J = 9.5 Hz, 3H, H-3'), 3.49-3.45 (m, 3H, H-9b), 3.30 (dd, J = 9.5, 8.4 Hz, 3H, H-2'), 2.09-1.99 (m, 6H, H-8).
^{13}C-NMR (D_2O, 175 MHz): **δ** = 172.5 (CO_2H, C-6'), 163.5 (C_{Ar}, C-1, C-2, C-3), 145.4 ($C_{Triazole}$, C-5), 124.9 ($CH_{Triazole}$, C-6), 102.3 (CH, C-1'), 75.4 (CH, C-3'), 74.5 (CH, C-5'), 72.8 (CH, C-2'), 71.2 (CH, C-4'), 66.2 (CH_2, C-9), 46.9 (CH_2, C-7), 46.4 (CH_2, C-4), 29.3 (CH_2, C-8).

m/z calcd for $C_{39}H_{57}N_{15}O_{21}H_2$ $[M+2H]^{2+}$ 536.7000, **found MS(ESI)**: **$[M+2H]^{2+}$** = 536.700**8**.

b) Glycodendrimer 4b

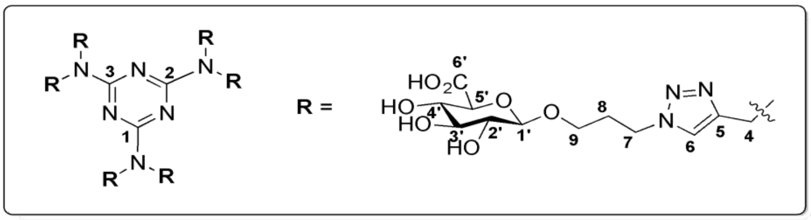

Following the general procedure a solution of protected hexamer **3b** (100 mg, 0.035 mmol) in 2 mL of anhydrous MeOH was sequentially treated with 0.5 N NaOMe/MeOH solution (27.0 equiv) and 0.2 M NaOH aq solution (9.0 equiv) to afford glycodendrimer **4b** in quantitative yield as a white powder.

^{1}H-NMR (D_2O, 700 MHz): **δ** = 7.73 (br s, 3H, H-9), 4.88 (s br, 6H, H-4), 4.41-4.33 (m, 6H, H-7), 4.38 (d, J = 8.4 Hz, 3H, H-1'), 3.89 (d, J = 9.5 Hz, 3H, H-5'), 3.71-3.65 (m, 3H, H-9a), 3.54 (t, J = 9.5 Hz, 3H, H-4'), 3.48 (t, J = 9.5 Hz, 3H, H-3'), 3.46-3.41 (m, 3H, H-9b), 3.32 (dd, J = 9.5, 8.4 Hz, 3H, H-2'), 2.10-2.01 (m, 6H, H-8).
^{13}C-NMR (D_2O, 125 MHz): **δ** = 172.3 (CO_2H, C-6'), 164.1 (C_{Ar}, C-1, C-2, C-3), 144.5 ($C_{Triazole}$, C-5), 124.4 ($CH_{Triazole}$, C-6), 102.2 (CH, C-1'), 75.2 (CH, C-3'), 74.5 (CH, C-5'), 72.6 (CH, C-2'), 71.2 (CH, C-4'), 66.3 (CH_2, C-9), 46.9 (CH_2, C-7), 42.3 (CH_2, C-4), 29.4 (CH_2, C-8).

m/z calcd for $C_{75}H_{108}N_{24}O_{42}H$ $[M+H]^+$ 2017.7126, **found MS(MALDI-TOF)**: **$[M+H]^+$** = 2017.7142

c) **Glycodendrimer 4c**

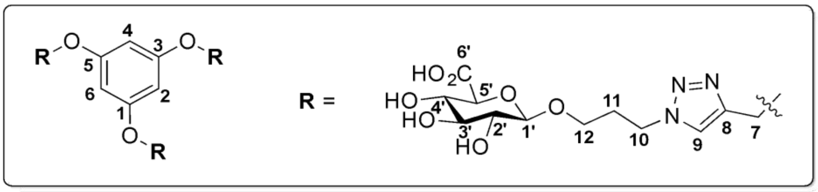

Following the general procedure a solution of protected trimer **3c** (100 mg, 0.0695 mmol) in 2 mL of anhydrous MeOH was treated sequentially with 0.5 N NaOMe/MeOH solution (13.5 equiv) and 0.2 M NaOH aq solution (4.5 equiv) to afford glycodendrimer **4c** in quantitative yields as a white powder.

^{1}H-NMR (D_2O, 500 MHz): **δ** = 7.98 (br s, 3H, H-9), 6.16 (br s, H-2, H-4, H-6), 5.00 (s br, 6H, H-7), 4.49-4.41 (m, 6H, H-10), 4.33 (d, J = 7.9 Hz, 3H, H-1'), 3.86 (d, J = 9.6 Hz, 3H, H-5'), 3.75-3.68 (m, 3H, H-12a), 3.55 (t, J = 9.6 Hz, 3H, H-4'), 3.49 (t, J = 9.6 Hz, 3H, H-3'), 3.50-3.43 (m, 3H, 12b), 3.32 (dd, J = 9.6, 7.7 Hz, 3H, H-2'), 2.14-2.06 (m, 6H, H-11).
^{13}C-NMR (D_2O, 125 MHz): **δ** = 172.4 (CO_2H, C-6'), 159.3 (C_{Ar}, C-1, C-3, C-5), 142.9 ($C_{Triazole}$, C-8), 125.5 ($CH_{Triazole}$, C-9), 102.2 (CH, C-1'), 95.8 (C_{Ar}, C-2, 4, 6), 75.2 (CH, C-3'), 74.6 (CH, C-5'), 72.7 (CH, C-2'), 71.3 (CH, C-4'), 66.3 (CH_2, C-12), 61.1 (CH_2, C-7), 47.0 (CH_2, C-10), 29.4 (CH_2, C-11).

m/z calcd for $C_{42}H_{57}N_9O_{24}H$ $[M+H]^+$ 1072.3589, **found MS(MALDI-TOF)**: **$[M+H]^+$** = 1072.3595

d) **Glycodendrimer 4d**

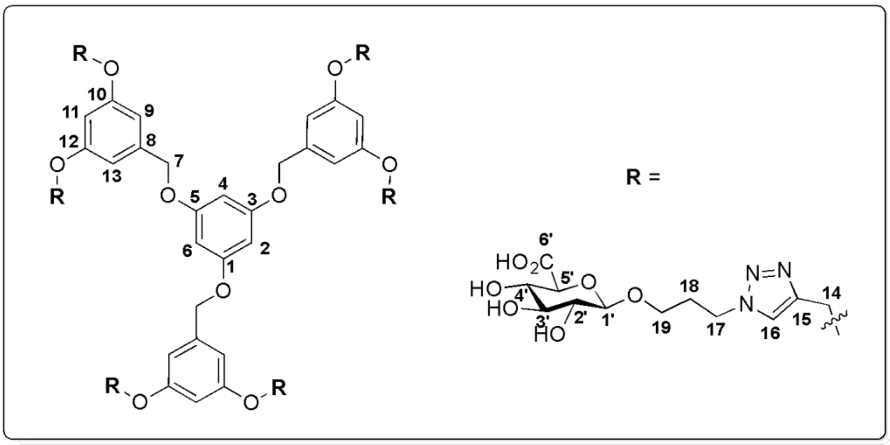

Following the general procedure a solution of protected hexamer **3d** (100 mg, 0.031 mmol) in 2 mL of anhydrous MeOH was sequentially treated with 0.5 N NaOMe/MeOH solution (27.0 equiv) and 0.2 M NaOH aq solution (9.0 equiv) to afford glycodendrimer **4d** in quantitative yield as a white powder.

^{1}H-NMR (D_2O, 500 MHz): **δ** = 7.67 (br s, 6H, H-16), 6.28 (br s, 9H, H-9, H-11, H-13), 5.85 (br s, 3H, H-2, H-4, H-6), 4.70-4.63 (br s, 12H, H-14), 4.54-4.30 (m, 12H, H-17), 4.27-4.17 (m, 6H, H-7), 4.25 (d, J = 7.9 Hz, 6H, H-1'), 3.79 (d, J = 9.6 Hz, 6H, H-5'), 3.64-3.53 (m, 6H, H-19a), 3.47 (t, J = 9.6 Hz, 6H, H-4'), 3.40 (t, J = 9.6 Hz, 6H, H-3'), 3.38 - 3.29 (m, 6H, 19b), 3.23 (dd, J = 9.6, 7.7 Hz, 6H, H-2'), 1.98-1.75 (m, 12H, H-18).

^{13}C-NMR (D_2O, 125 MHz): **δ** = 171.9 (CO_2H, C-6’), 165.7 (C_{Ar}, C-1, C-3, C-5), 159.0 (C_{Ar}, C-10, C-12), 142.8 ($C_{Triazole}$, C-15), 139.4 (C_{Ar}, C-8), 124.8 ($CH_{Triazole}$, C-16), 117.0 (C_{Ar}, C-9, C-13), 106.4 (C_{Ar}, C-2, C-4, C-6, C-11), 102.3 (CH, C-1'), 75.2 (CH, C-4'), 74.4 (CH, C-5’), 72.6 (CH, C-3’), 71.2 (CH, C-2’), 66.4 (CH_2, C-19), 60.9 (CH_2, C-14), 47.1 (CH_2, C-7), 47.0 (CH_2, C-17), 29.5 (CH_2, C-18).

m/z calcd for $C_{99}H_{126}N_{18}O_{51}H$ [M+H]$^+$ 2383.7892, **found MS(MALDI-TOF)**: **[M+H]$^+$ MS(MALDI-TOF)**: **[M+H]$^+$** = 2383.7912.

e) Glycodendrimer 4e

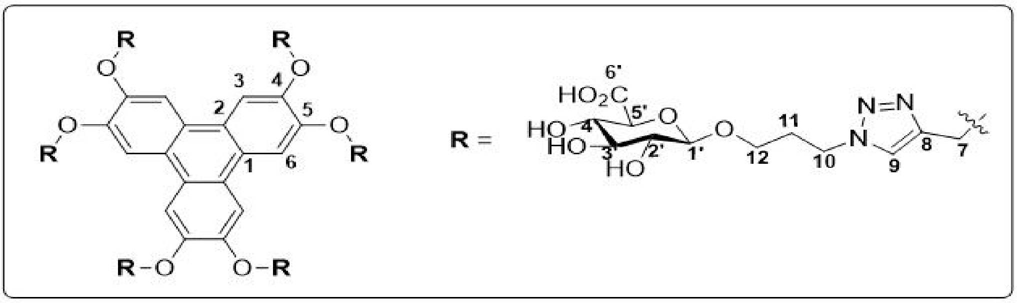

Following the general procedure a solution of protected hexamer **3e** (60 mg, 0.0196 mmol) in 5 mL of anhydrous MeOH was sequentially treated with 0.5 N NaOMe/MeOH solution (27 equiv) for 48 hours and 0.2 M NaOH aq solution (9 equiv) for 48 hours to afford glycodendrimer **4e** in quantitative yield as a brown powder.

^{1}H-NMR (D_2O, 700 MHz): **δ** = 7.88 (br s, 6H, H-9), 7,28 (br s, 6H, H-3, H-6), 5.15 (br s, 12H, H-7), 4.29 (br signal, 18H, H-10, H-1’), 3,69-3,03 (br m, 36H, H-12, H-2’, H-3’, H-4’, H-5’), 1.99 (br signal, 12H, H-11).

^{13}C-NMR (D_2O, 175 MHz): **δ** = 174.7 (CO_2H, C-6’), 146,6 (C_{Ar}, C-4, C-5), 142.8 ($C_{Triazole}$, C-8), 126,1 ($CH_{Triazole}$, C-9), 122,7 (C_{Ar}, C-1, C-2), 107,0 (CH_{Ar}, C-3, C-6), 102.0 (CH, C-1'), 75.3 (2 × CH, C-3', C-5’), 72.7 (CH, C-2’), 71.5 (CH, C-4’), 66.1 (CH_2, C-12), 61.6 (CH_2, C-7), 47.0 (CH_2, C-10), 29.9 (CH_2, C-11).

m/z calcd for $C_{90}H_{114}N_{18}O_{48}H$ [M+H]$^+$ 2215.7106, **found MS(MALDI-TOF)**: **[M+H]$^+$ MS(MALDI-TOF)**: **[M+H]$^+$** = 2215.7116

f) Glycodendrimer 4f

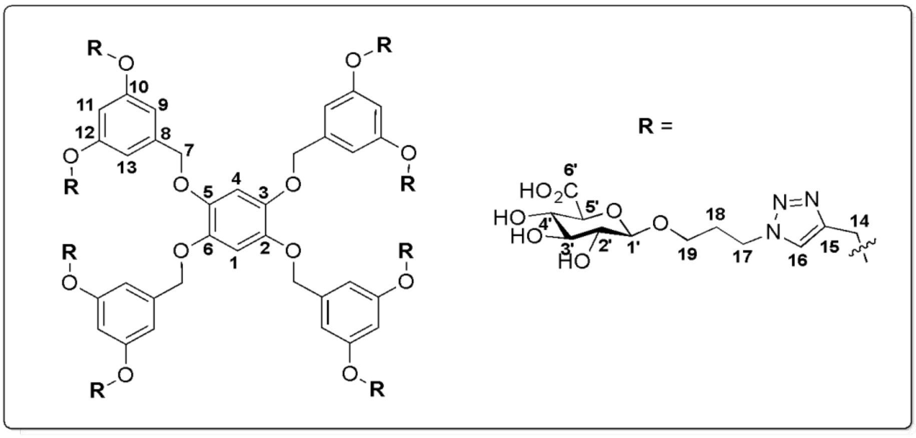

Following the general procedure a solution of protected octamer **3f** (100 mg, 0.0234 mmol) in 2 mL of anhydrous MeOH was sequentially treated with 0.5 N NaOMe/MeOH solution (36.0 equiv) and 0.2 M NaOH aq solution (12.0 equiv) to afford glycodendrimer **4f** in quantitative yield as a white powder.

^{1}H-NMR (D_2O, 500 MHz): **δ** = 8.01 (br s, 8H, H-16), 6.61 (d, *J* = 2.2 Hz, 8H, H-9, H-13), 6.46 (t, *J* = 2.2 Hz, 4H, H-11), 6.29 (s, 2H, H-1, H-4), 5.11 (s, 16H, H-14), 4.50 (s, 8H, H-7), 4.50-4.45 (m, 16H, H-17), 4.34 (d, *J* = 7.7 Hz, 8H, H-1'), 3.89 (d, *J* = 9.6 Hz, 8H, H-5'), 3.74-3.69 (m, 8H, H-19a), 3.55 (t, *J* = 9.4 Hz, 8H, H-4'), 3.50-3.45 (m, 8H, H-19b), 3.48 (t, *J* = 9.4 Hz, 8H, H-3'), 3.30 (dd, *J* = 9.6, 7.7 Hz, 8H, H-2'), 2.16-2.08 (m, 16H, H-18).
^{13}C-NMR (D_2O, 125 MHz): **δ** 172.1 (CO_2H, C-6'), 158.6 (C_{Ar}, C-10, C-12), 143.6 (C_{Ar}, C-2, C-3, C-5, C-6), 142.9 ($C_{Triazole}$, C-15), 129.4 (C_{Ar}, C-8), 125.6 ($CH_{Triazole}$, C-16), 107.1 (C_{Ar}, C-9, C-13), 102.2 (CH, C-1'), 101.8 (C_{Ar}, C-11), 100.4 (C_{Ar}, C-1, C-4), 75.2 (CH, C-4'), 74.3 (CH, C-5'), 71.6 (CH, C-2'), 71.2 (CH, C-3'), 66.4 (CH_2, C-19), 63.4 (CH_2, C-7), 61.2 (CH_2, C-14), 47.1 (CH_2, C-17), 29.4 (CH_2, C-18).

m/z calcd for $C_{130}H_{166}N_{24}O_{68}H$ $[M+H]^+$ 3152.0342, **found MS(MALDI-TOF)**: **$[M+H]^+$ MS(MALDI-TOF)**: **$[M+H]^+$** = 3152.0357

g) Glycodendrimer 9a

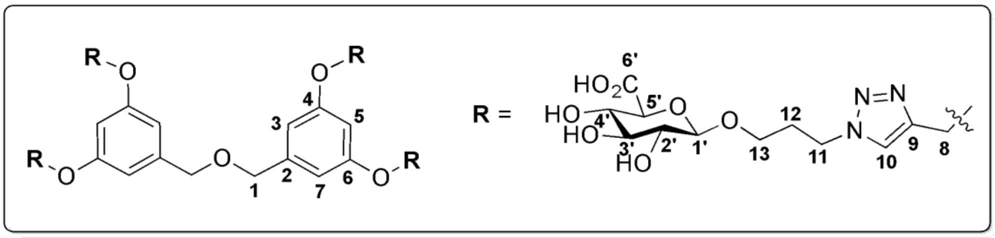

Following the general procedure a solution of protected tetramer **8a** (126 mg, 0.061 mmol) in 5 mL of anhydrous MeOH was sequentially treated with 0.5 N NaOMe/MeOH solution (18 equiv) for 20 h and 0.2 M NaOH aq solution (6 equiv) for 20 h to afford glycodendrimer **9a** in quantitative yield as a white powder.

^{1}H NMR (D_2O, 250 MHz): **δ** = 7.70 (s, 4H, H-10), 6.33-6.15 (m, 6H, H-3, H-5, H-7), 4.88-4.74 (m, 8H, H-8), 4.27-4.12 (m, 12H, H-11, H-1'), 4.05-3.93 (m, 4H, H-4'), 3.70

(d, J = 9.0 Hz, 4H, H-5’), 3.55-3.47 (m, 4H, H-13a), 3.46-3.24 (m, 12H, H-1, H-3’, H-13b), 3.17-3.12 (m, 4H, H-2’), 1.84 (s, 8H, H-12). (66H)
^{13}C NMR (D_2O, 62.5 MHz): **δ** = 172.6 (CO_2H, C-6’), 159.2 (C_{Ar}, C-4, C-6), 143.2 ($C_{Triazole}$, C-9), 141.0 (C_{Ar}, C-2), 125.4 ($CH_{Triazole}$, C-10), 107.3 (C_{Ar}, C-3, C-7), 102.6 (CH, C-1’), 101.4 (CH, C-5), 75.5 (CH, C-3’ or C-4’), 74.8 (CH, C-5’), 73.0 (CH, C-2’), 71.6 (CH, C-3’ or C-4’), 66.7 (CH_2, C-1, C-13), 61.2 (CH_2, C-8), 47.4 (CH_2, C-11), 29.8 (CH_2, C-12).

m/z calcd for $C_{62}H_{82}N_{12}O_{33}H_2$ [M+2H]$^{2+}$ 762.2626, **found MS(ESI)**: **[M+2H]$^{2+}$** = 762.2643.

h) Glycodendrimer 9b

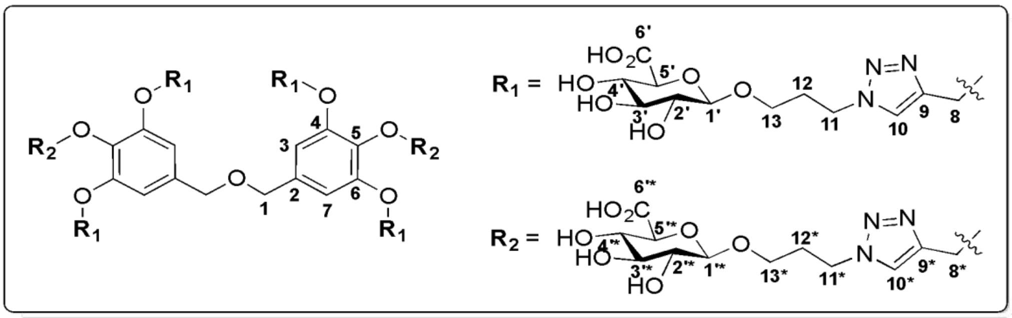

Following the general procedure a solution of protected hexamer **8b** (99.3 mg, 0.033 mmol) in 7 mL of anhydrous MeOH was sequentially treated with 0.5 N NaOMe/MeOH solution (27 equiv) for 48 hours and 0.2 M NaOH aq solution (9 equiv) for 48 hours to afford glycodendrimer **9b** in quantitative yield as a white powder.

^{1}H NMR (D_2O, 250 MHz): **δ** = 7.96 (s, 4H, H-10), 7.72 (s, 2H, H-10*), 6.64 (s, 4H, H-3, H-7), 5.08-4.89 (m, 12H, H-8, H-8*), 4.48-4.24 (m, 22H, H-1, H-11, H-11*, H-1’, H-1’*), 3.81 (d, J = 8.7 Hz, 6H, H-5’, H-5’*), 3.74-3.56 (m, 6H, H-13a, H-13a*), 3.57-3.35 (m, 18H, H-3’, H-3’*, H-4’, H-4’*, H-13b, H-13b*), 3.26 (t, J = 8.2 Hz, 6H, H-2’, H-2’*), 2.08-1.87 (m, 12H, H-12, H-12*).

^{13}C NMR (D_2O, 62.5 MHz): **δ** = 172.9 (CO_2H, C-6’), 151.4 (C_{Ar}, C-4, C-6), 143.2 ($C_{Triazole}$, C-9), 135.1 (C_{Ar}, C-2) 134.9 (C_{Ar}, C-5), 125.81 ($CH_{Triazole}$, C-10), 108.0 (C_{Ar}, C-3, C-7), 102.2 (CH, C-1’, C-1’*), 75.3 (CH, C-3' and C-3’*, or C-4’ and C-4’*), 74.7, 72.7 (CH, C-5’, C-5’*), 71.8 (CH, C-2’, C-2’*), 71.4 (CH, C-3' and C-3’*, or C-4’ and C-4’*), 66.3 (CH_2, C-13, C-13*), 66.2 (CH_2, C-1), 62.0 (CH_2, C-8, C-8*), 47.1 (CH_2, C-11), 46.9 (C-11*), 29.5 (CH_2, C-12*), 29.4 (CH, C-12).

m/z calcd for $C_{86}H_{116}N_{18}O_{49}H$ [M+H]$^{+}$ 2185.7211, **found MS(MALDI-TOF)**: **[M+H]$^{+}$** = 2185.7221.

i) Glycodendrimer 9c

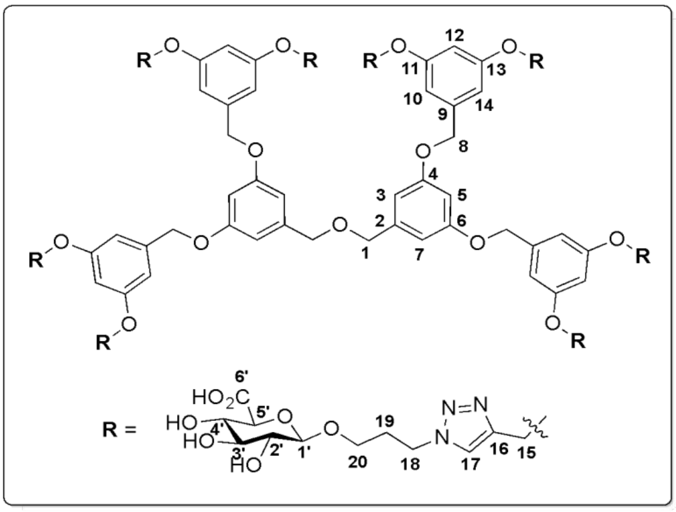

Following the general procedure a solution of protected octamer **8c** (113 mg, 0.026 mmol) in 8 mL of anhydrous MeOH was sequentially treated with 0.5 N NaOMe/MeOH solution (36 equiv) for 48 h and 0.2 M NaOH aq solution (12 equiv) for 48 h to afford glycodendrimer **9c** in quantitative yield as a white powder.

^{1}H NMR (D_2O, 250 MHz): **δ** = 7.67 (s, 8H, H-17), 6.38-5.91 (m, 18H, H-3, H-5, H-7, H-10, H-12, H-14), 4.74-4.51 (m, 16H, H-15), 4.47-4.00 (m, 32H, H-8, H-18, H-1'), 3.80 (d, J = 8.3 Hz, 8H, H-5'), 3.63-3.15 (m, 44H, H-1, H-20, H-2', H-3', H-4'), 1.87 (s, 16H, H-19).

^{13}C NMR (D_2O, 62.5 MHz): **δ** = 172.3, (CO_2H, C-6'), 159.7 (C_{Ar}, C-4, C-6), 159.3 (C_{Ar}, C-11, C-13), 143.1 ($C_{Triazole}$, C-16), 139.6 (C_{Ar}, C-2, C-9), 125.1 ($CH_{Triazole}$, C-17), 106.6 (CH, C-3, C-7, C-10, C-14), 102.6 (CH, C-1'), 101.4 (CH, C-5, C-12), 75.5 (CH, C-3' or C-4'), 74.8 (CH, C-5'), 73.0 (CH, C-2'), 71.6 (CH, C-3' or C-4'), 66.7 (CH_2, C-1, C-20), 61.0 (CH_2, C-15), 47.4 (CH_2, C-8, C-18), 29.8 (CH_2, C-19).

m/z calcd for $C_{138}H_{174}N_{24}O_{69}H$ [M+H]$^+$ 3273.0956, **found MS(MALDI-TOF)**: **[M+H]$^+$** = 3273.0916.

j) Glycodendrimer 12a

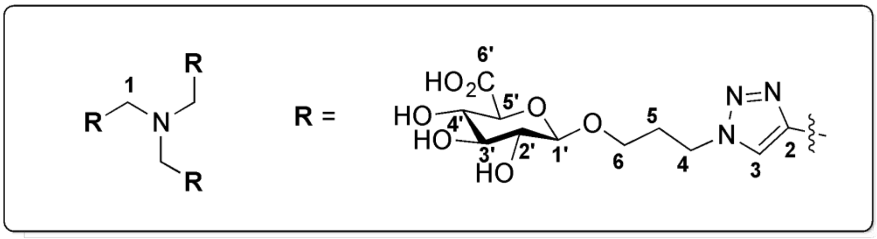

Following the general procedure a solution of protected trimer **11a** (100 mg, 0.0723 mmol) in 2 mL of anhydrous MeOH was sequentially treated with 0.5 N NaOMe/MeOH solution (13.5 equiv) and 0.2 M NaOH aq solution (4.5 equiv) to afford glycodendrimer **12a** in quantitative yield as a white powder.

^{1}**H-NMR** (D_2O/CD_3OD (9:1), 500 MHz): **δ** = 8.27 (br s, 3H, H-3), 4.61-4.45 (m, 6H, H-4), 4.54 (br s, 6H, H-1), 4.44 (d, *J* = 7.7 Hz, 3H, H-1'), 3.90 (d, *J* = 9.6 Hz, 3H, H-5'), 3.85-3.78 (m, 3H, H-6a), 3.64-3.58 (m, 3H, H-6b), 3.51 (t, *J* = 9.4 Hz, 3H, H-4'), 3.46 (t, *J* = 9.4 Hz, 3H, H-3'), 3.27 (overlapped H-2'), 2.25-2.14 (m, 6H, H-5).

^{13}**C-NMR** (D_2O/CD_3OD (9:1), 125 MHz): **δ** = 173.1 (CO_2H, C-6'), 136.9 ($C_{Triazole}$, C-2), 126.3 ($CH_{Triazole}$, C-3), 103.3 (CH, C-1'), 76.3 (CH, C-3'), 75.5 (CH, C-5'), 73.8 (CH, C-2'), 72.3 (CH, C-3'), 67.5 (CH_2, C-6), 48.4 (overlapped CH_2, C-1), 30.4 (CH_2, C-5).

m/z calcd for $C_{36}H_{54}N_{10}O_{21}H$ [M+H]$^+$ 963.3538, **found MS(ESI)**: **[M+H]$^+$** = 963.3543

k) Glycodendrimer 12b

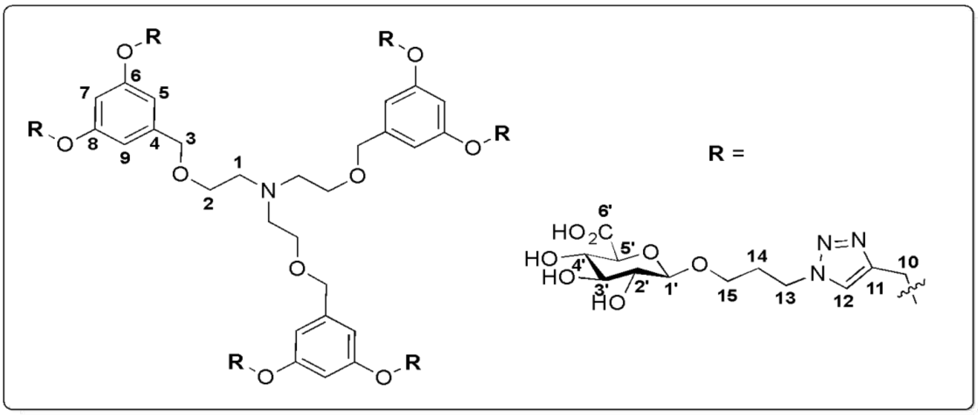

Following the general procedure a solution of protected hexamer **11b** (100 mg, 0.0308 mmol) in 2 mL of anhydrous MeOH was sequentially treated with 0.5 N NaOMe/MeOH solution (27.0 equiv) and 0.2 M NaOH aq solution (9.0 equiv) to afford glycodendrimer **12b** in quantitative yield as a white powder.

^{1}**H-NMR** (D_2O, 700 MHz): **δ** = 7.96 (br s, 6H, H-12), 6.64 (d, *J* = 2.2 Hz, 6H, H-5, H-9), 6.50 (t, *J* = 2.2 Hz, 3H, H-7), 4.77 - 4.68 (br s, 12H, H-10), 4.52 - 4.32 (m, 12H, H-13), 4.30 (d, *J* = 7.8 Hz, 6H, H-1'), 4.17 - 4.05 (m, 6H, H-3), 3.81 (d, *J* = 9.6 Hz, 6H, H-5'), 3.67 - 3.53 (m, 12H, H-2, H-15a), 3.50 (t, *J* = 9.6 Hz, 6H, H-4'), 3.42 (t, *J* = 9.6 Hz, 6H, H-3'), 3.40 - 3.28 (m, 6H, 15b), 3.21 (dd, *J* = 9.6, 7.8 Hz, 6H, H-2'), 2.96 – 2.86 (m, 6H, H-1), 2.01-1.82 (m, 12H, H-14).

^{13}**C-NMR** (D_2O, 175 MHz): **δ** = 172.0 (CO_2H, C-6'), 158.8 (C_{Ar}, C-6, C-8), 142.8 ($C_{Triazole}$, C-11), 139.0 (C_{Ar}, C-4), 125.2 ($CH_{Triazole}$, C-12), 117.0 (C_{Ar}, C-5, C-9), 104.2 (C_{Ar}, C-7), 102.3 (CH, C-1'), 75.3 (CH, C-4'), 74.4 (CH, C-5'), 72.9 (CH_2, C-3), 72.7 (CH, C-3'), 71.1 (CH, C-2'), 66.8 (CH_2, C-2), 66.4 (CH_2, C-15), 61.1 (CH_2, C-10), 54.6 (CH_2, C-1), 47.0 (CH_2, C-13), 29.5 (CH_2, C-14).

m/z calcd for $C_{99}H_{135}N_{19}O_{51}H$ [M+H]$^+$ 2406.8627, **found MS(MALDI-TOF)**: **[M+H]$^+$** = 2406.8638.

l) Glycodendrimer 12c

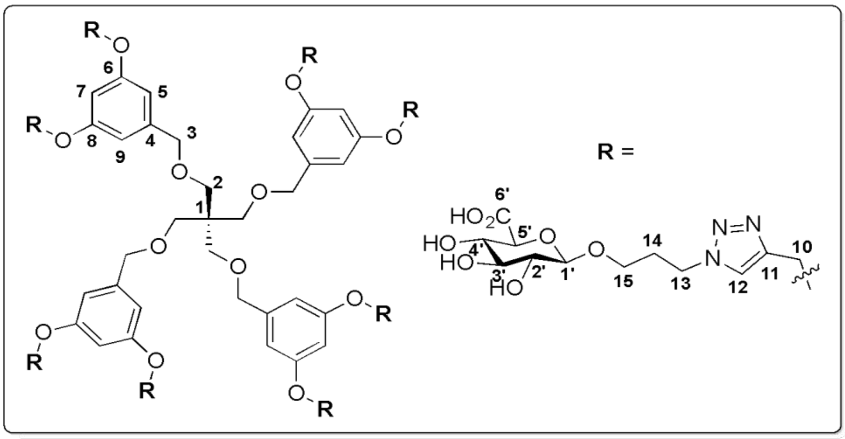

Following the general procedure, a solution of protected octamer **11c** (100 mg, 0.0231 mmol) in 2.0 mL of anhydrous MeOH was sequentially treated with 0.5 N NaOMe/MeOH solution (36.0 equiv) and 0.2 M NaOH aq solution (12.0 equiv) to afford glycodendrimer **12c** in quantitative yield as a white powder.

^{1}H-NMR (D_2O, 700 MHz): **δ** = 7.98 (br s, 8H, H-12), 6.62 (d, *J* = 2.2 Hz, 8H, H-5, H-9), 6.49 (t, *J* = 2.2 Hz, 4H, H-7), 4.75 - 4.65 (br s, 16H, H-10), 4.49-4.35 (m, 12H, H-13), 4.27 (d, *J* = 7.8 Hz, 8H, H-1'), 4.12 - 4.00 (m, 8H, H-3), 3.79 (d, *J* = 9.6 Hz, 8H, H-5'), 3.62-3.39 (m, 16H, H-2, H-15a), 3.52 (t, *J* = 9.6 Hz, 8H, H-4'), 3.41 (t, *J* = 9.6 Hz, 8H, H-3'), 3.45 - 3.31 (m, 8H, 15b), 3.19 (dd, *J* = 9.6, 7.8 Hz, 8H, H-2'), 1.99 - 1.80 (m, 16H, H-14).

^{13}C-NMR (D_2O, 175 MHz): **δ** = 171.9 (CO_2H, C-6'), 159.1 (C_{Ar}, C-6, C-8), 143.0 ($C_{Triazole}$, C-11), 138.7 (C_{Ar}, C-4), 125.1 ($CH_{Triazole}$, C-12), 116.8 (C_{Ar}, C-5, C-9), 104.1 (C_{Ar}, C-7), 102.0 (CH, C-1'), 75.2 (CH, C-4'), 74.3 (CH, C-5'), 73.1 (CH_2, C-3), 72.5 (CH, C-3'), 71.0 (CH, C-2'), 66.5 (CH_2, C-2), 66.2 (CH_2, C-15), 61.0 (CH_2, C-10), 46.9 (CH_2, C-13), 45.3 (CH_2, C-1), 29.3 (CH_2, C-14).

m/z calcd for $C_{129}H_{172}N_{24}O_{68}H$ $[M+H]^+$ 3146.0812, **found MS(MALDI-TOF)**: **$[M+H]^+$** = 3146.0826.

4. Figure S1

5. A (A_1)

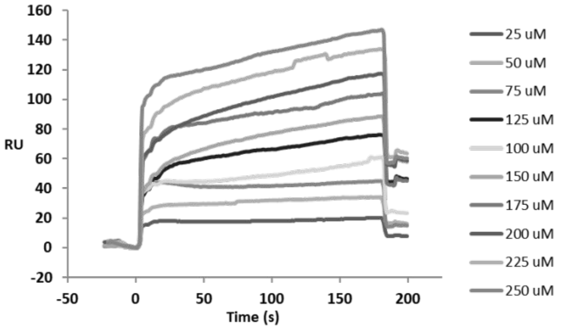

(A_2)

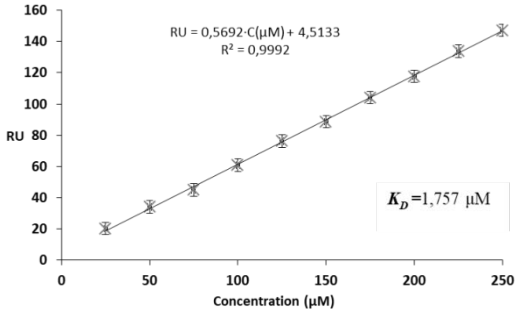

B (B_1)

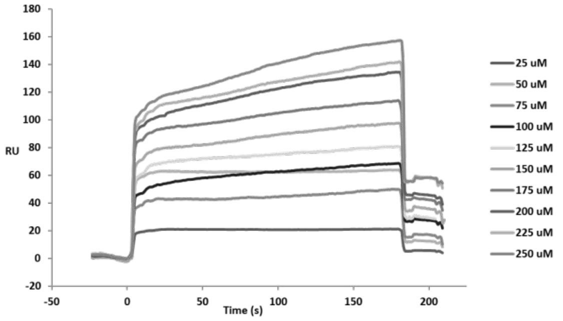

(B_2)

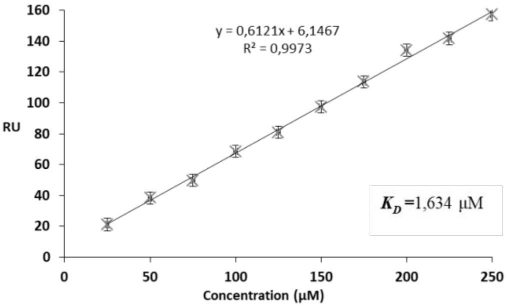

Figure S1. A Binding responses of different concentrations of GD **4c** (A_1) and **9b** (B_1) with DENV2 immobilized on the CM4 chip, showing association and dissociation phases. Responses were reference subtracted and blank corrected. B. Steady state affinity study of the interaction between GD **4c** (A_2) and **9b** (B_2) and DENV2 immobilized on a CM4 chip.

5. Computational Methods

Protein Structure

We used the Cartesian coordinates for a 3D apo form of envelope protein (E) Dengue Virus 2 Thailand 16681/84 with PDB accession code 3C5X[8]. The protocol for protein preparation for MD simulations was the same as we have described in previous works.[6]

Docking

Ligand preparation. 3D structures for compounds hexamer 12b and octamer 12c were built using the interactive molecular graphics program PyMOL[9] (URL: https://pymol.org/2/) and the carbohydrate builder web server glycam (URL: https://glycam.org/). The ground state geometry of the ligand was optimized using the program Gaussian 16 (URL: https://gaussian.com/gaussian16/)[10] and fitted to the atoms as AMBER atom types and RESP charges using the program antechamber (AmberTools 20, URL: https://ambermd.org/)[11].

We used AutoDock Vina (URL: http://vina.scripps.edu)[12] as docking program, adopting a basic docking protocol (flexible ligand and rigid receptor) to predict the 3D geometry of the complexes formed by the envelope protein Dengue Virus 2 with dendrimers. Hexadendrimer XX and octadendrimer XX were docked in the domain III (DIII) of the protein (residues 292-395).[13] The most favorable poses were selected according to its predicted docking energy and visual inspection. The resultant complexes were used as the starting point for molecular dynamics studies.

Molecular dynamics simulation

The selected complexes for **12b** and **12c** were immersed in cubic boxes of TIP3P water molecules[14] large enough to guarantee that the shortest distance between the solute and the edge of the box was greater than 15 Å. Counterions were also added to maintain electro neutrality. We performed molecular dynamics simulations using AMBER 20 package.[11] The starting structures were simulated in the NPT ensemble with the periodic boundary conditions and particle mesh Ewald method to treat long-range electrostatic effects.[15] The protocol was as follows: three consecutive minimizations were performed: (i) involving only hydrogen atoms, (ii) involving only the water molecules and ions, and (iii) involving the entire system. The system was then heated and equilibrated in two steps: (i) 20 ps of MD heating the whole system from 100 to 300 K and (ii) equilibration of the entire system during 100 ps at 300 K. The equilibrated structure was the starting points for 50 ns of MD simulations carried out using the *pmemd_cuda.SPFP* at constant temperature (300K) and pressure (1 atm) and the standard ff14SB force field parameter. The constraint algorithm SHAKE[16] was used to keep bonds involving H atoms at their equilibrium length, allowing a 2 fs time step for the integration of Newton's equations of motion. The *cpptraj*[17] module in AMBER20 was employed for data processing and geometry analysis of the calculated trajectories.

Analysis of MD trajectories.

The stability of the complexes (DENV2-glycodendrimer **12b** and DENV2-glycodendrimer **12c**) were evaluated by calculating the root-mean-square-deviation (RMSD) of the Cα atoms along the trajectories, using their starting structures as reference. Additionally, the root-mean-square-fluctuation (RMSF) of each residue, relative to the corresponding average value, was calculated once each snapshot had been fitted to its initial structure. The effective binding free energies between the ligands and the more relevant residues in the binding site were qualitatively estimated using the MM/GBSA.[18] MM/GBSA is a popular approach to estimate the free energy of the binding of small ligands to biological macromolecules. MM/GBSA takes into account a MM interaction term, a solvation contribution through a generalized born (GB) model, and a surface area (SA) contribution to account for the non-polar part of desolvation. The MM part estimates the enthalpic contributions for the protein–ligand interactions (bonded, electrostatic, Van der Waals). The polar solvation energy represents the electrostatic interaction between the solute and the continuum solvent. In addition, three non-polar solvation terms include cavitation, dispersion, and repulsion energies, representing the cost of making a cavity in the solvent, as well as the attractive and repulsive parts of the van der Waals interactions between the solute and the solvent. All these three non-polar solvation terms are free energies and in particular the cavitation energy should have important entropic components, representing the reorganization of the solvent around the solute. In summary, in MM/GBSA, the free energy of a state is estimated from the following sum: $G = Ebnd + Eel + EwdW + Gpol + Gnp - TS$. A 12–6 Lennard-Jones term was used to model de MM contribution. For GB, the solute dielectric constant was set to four while that of the solvent was set to 80, and the dielectric boundary was calculated using a solvent probe radius of 1.4 Å. The polar contribution is calculated using GB, and the non-polar energy is estimated by solvent accessible surface area (SASA).

6. Figures S2-S8

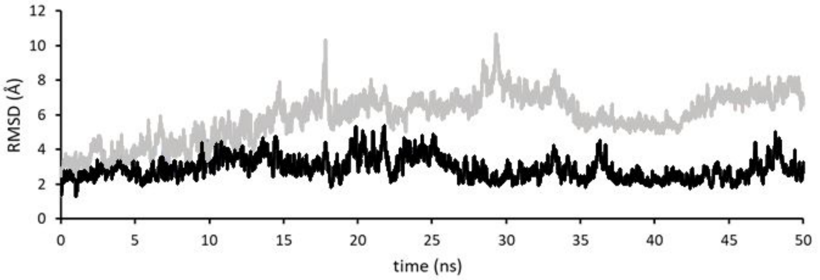

Figure S2. RMSD values along the simulation for DENV2-**12b** relatives to the starting structure. The Y axis shows the RMSD values in Å and the X axis the time in nanoseconds. RMSD values are represented in black for the protein DENV2 and in grey for the dendrimer.

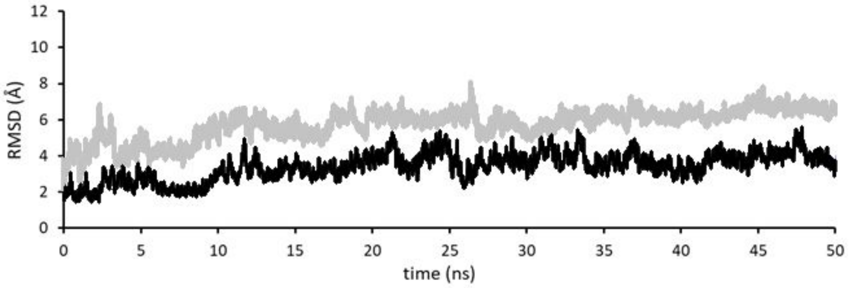

Figure S3. RMSD values along the simulation for DENV2-**12c** relatives to the starting structure. The Y axis shows the RMSD values in Å and the X axis the time in nanoseconds. RMSD values are represented in black for the protein DENV2 and in grey for the dendrimer.

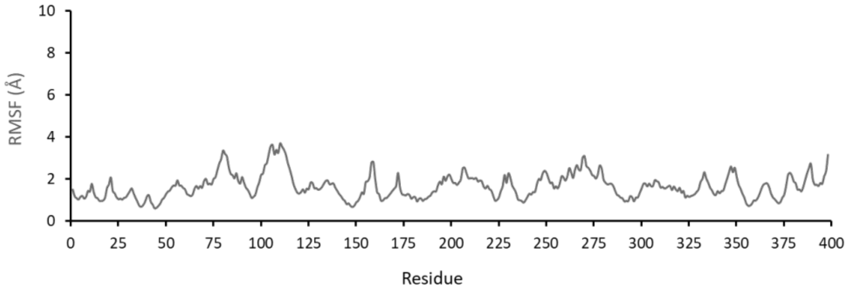

Figure S4. Average fluctuations of the residues of the DENV2-**12b** complex during the simulation. Y axis represents Cα RMSD in Å and X axis, residue number of DENV2.

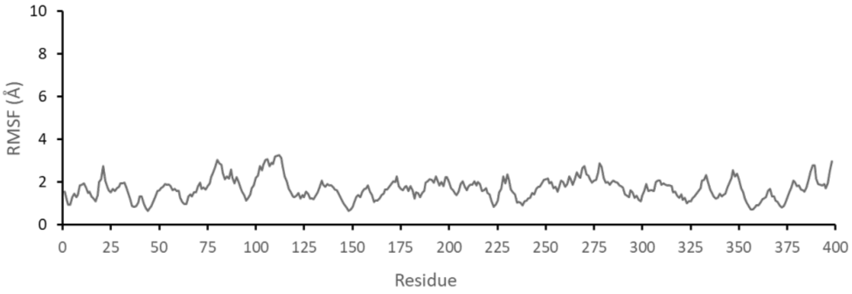

Figure S5. Average fluctuations of the residues of the DENV2-**12c** complex during the simulation. Y axis represents Cα RMSD in Å and X axis, residue number of DENV2.

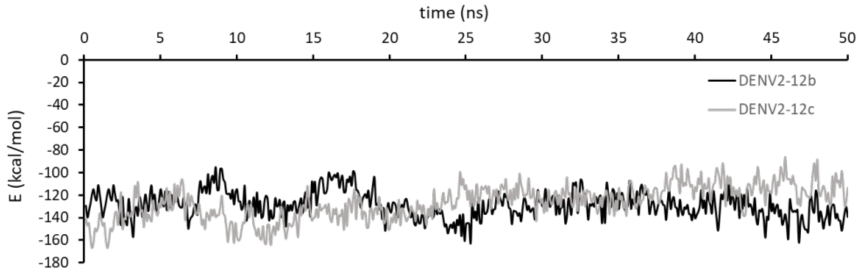

Figure S6. 2D free energy plot for DENV2-**12b** and DENV2-**12c** complexes. The dark grey line shows the energy value at each step of the simulation for DENV2-**12b** complex and light grey line for DENV2-**12c** complex. The X axis shows time in nanoseconds, the left Y axis shows global energy values (kcal/mol).

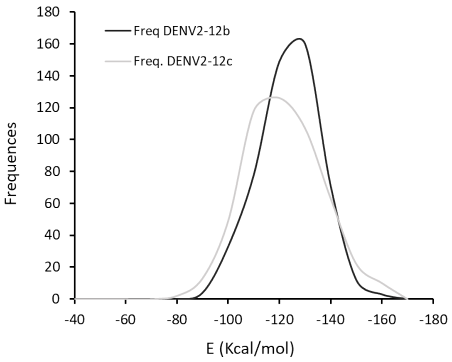

Figure S7. 2D free energy plot of the density energy values achieved by the complexes during the simulation. Dark grey line shows values for DENV2-**12b** and light grey line for DENV2-**12c** complexes. The right Y axis shows the frequency of the values and X axis the energy values (kcal/mol).

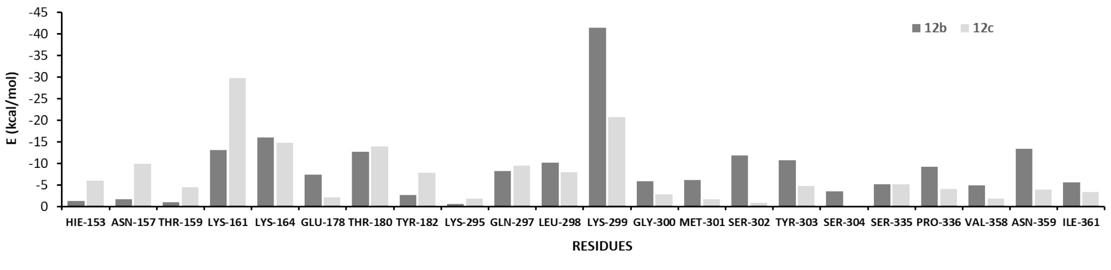

Figure S8. 2D free energy per-residue interaction plot of **12b** (dark grey) and **12c** (light grey). X axis shows the interaction energies, and Y axis the name and number of DENV2 residues.

7. Table S1

Table S1. Description of the interactions in complexes DENV2-**12b** and DENV2-**12c**.

DENV2	DENV2-12b	E (kcal/mol)	Type of interaction	DENV2-12c	E (kcal/mol)	Type of interaction
HIE-153	triazol ring	-1,22	vdW	GlucA	-6,03	HB
ASN-157	GlucA	-1,75	HB	GlucA	-9,96	HB + vdW
THR-159	carbon chain	-1,07	vdW	carbon chain	-4,44	vdW
LYS-161	phenyl ring	-13,11	vdW + HB	GlucA + phenyl ring + triazol ring	-29,79	qq + vdW
HIE-162	triazol ring	-2,92	vdW	GlucA	-3,25	HB
LYS-164	GlucA	-16,04	qq	GlucA	-14,71	qq + vdW
GLU-178	GlucA	-7,39	HB	GlucA	-2,09	HB
LEU-179	carbon chain	-4,82	vdW	carbon chain	-4,30	vdW
THR-180	GlucA	-12,73	HB	GlucA + phenyl ring	-13,99	HB + vdW
GLY-181	phenyl ring	-3,49	vdW	carbon chain	-3,37	vdW
TYR-182	triazol ring	-2,67	ππ	GlucA + carbon chain	-7,83	HB + vdW
LYS-295	GlucA	-0,52	vdW	GlucA	-1,9	HB + vdW
LEU-296	GlucA	-1,59	HB	GlucA	-0,93	HB
GLN-297	triazol ring + carbon chain	-8,20	vdW	GlucA	-9,49	HB
LEU-298	GlucA	-10,13	vdW	GlucA	-7,94	HB
LYS-299	GlucA + phenyl ring	-41,40	qq + vdW	GlucA	-20,68	HB + vdW
GLY-300	GlucA	-5,88	HB	triazol ring	-2,77	HB
MET-301	GlucA	-6,20	HB	GlucA	-1,66	vdW
SER-302	GlucA + triazol ring	-11,81	HB	carbon chain	-0,87	vdW
TYR-303	triazol ring	-10,67	ππ	triazol ring	-4,81	HB
SER-304	GlucA	-3,50	HB	GlucA	-0,16	HB
SER-335	GlucA	-5,11	HB	carbon chain	-5,23	vdW
PRO-336	GlucA	-9,18	HB + vdW	triazol ring	-4,11	vdW
VAL-358	GlucA	-4,85	HB	GlucA	-1,79	HB
ASN-359	GlucA	-13,39	qq + vdW	GlucA	-3,95	HB
ILE-361	GlucA	-5,53	vdW	carbon chain	-3,33	vdW
THR-363	carbon chain	-3,45	vdW	carbon chain	-4,76	vdW
LYS-365	GlucA	-0,56	vdW	GlucA	-4,42	qq

*Type of interaction: charge-charge (qq), hydrogen bond (HB), van der Waals (vdW), π-π stacking (ππ)

8. References

(1) Malkoch, M.; Schleicher, K.; Drockenmuller, E.; Hawker, C. J.; Russell, T. P.; Wu, P.; Fokin, V. V. Structurally Diverse Dendritic Libraries: A Highly Efficient Functionalization Approach using Click Chemistry. *Macromolecules* **2005**, *38*, 3663-3678.

(2) Kawamoto, H.; Nakatsubo, F.; Murakami, K. O-Benzylation of Phloroglucinol via Phloroglucinol Triacetate. *Synthetic Commun.* **1996**, *26*, 531-534.

(3) Fukuda, T.; Matsumoto, E.; Onogi, S.; Miura, Y. Aggregation of Alzheimer Amyloid beta Peptide (1-42) on the Multivalent Sulfonated Sugar Interface. *Bioconjugate Chem.* **2010**, *21*, 1079-1086.

(4) Agrahari, A. K.; Jaiswal, M. K.; Yadav, M. S.; Tiwari, V. K. CuAAC Mediated Synthesis of Cyclen Cored Glycodendrimers of High Sugar Tethers at Low Generation. *Carbohyd. Res.* **2021**, *508*.

(5) Khanam, S.; Rai, S. K.; Verma, D.; Khanna, R. S.; Tewari, A. K. An Efficient and Controlled Synthesis of Persulfonylated G1 Dendrimers via Click Reaction. *Rsc. Adv.* **2016**, *6*, S6952-S6962.

(6) Garcia-Oliva, C.; Cabanillas, A. H.; Perona, A.; Hoyos, P.; Rumbero, A.; Hernaiz, M. J. Efficient Synthesis of Muramic and Glucuronic Acid Glycodendrimers as Dengue Virus Antagonists. *Chem. Eur. J.* **2020**, *26*, 1588-1596.

(7) Bhalla, V.; Singh, H.; Kumar, M.; Prasad, S. K. Triazole-Modified Triphenylene Derivative: Self-Assembly and Sensing Applications. *Langmuir* **2011**, *27*, 15275-15281.

(8) Li, L.; Lok, S. M.; Yu, I. M.; Zhang, Y.; Kuhn, R. J.; Chen, J.; Rossmann, M. G. The Flavivirus Precursor Membrane-Envelope Protein Complex: Structure and Maturation. *Science* **2008**, *319*, 1830-1834.

(9) Schrödinger, L. The PyMOL Molecular Graphics System, **2013**.

(10) Gaussian 09, R. A. 1, mj Frisch, GW trucks, HB Schlegel, Ge Scuseria, Ma Robb, JR Cheeseman, G. Scalmani, V. Barone, B. Mennucci, GA Petersson et al., Gaussian. *Inc., Wallingford CT* **2009**, *121*, 150-166.

(11) Case, D.; Belfon, K.; Ben-Shalom, I.; Brozell, S.; Cerutti, D.; Cheatham, T.; III, V. C.; Darden, T.; Duke, R.; Giambasu, G. AMBER2020, university of California, San Fransisco. *J. Amer. Chem. Soc.* **2020**, *142*, 3823-3835.

(12) Trott, O.; Olson, A. J. Software News and Update AutoDock Vina: Improving the Speed and Accuracy of Docking with a New Scoring Function, Efficient Optimization, and Multithreading. *J. Comput. Chem.* **2010**, *31*, 455-461.

(13) Watterson, D.; Kobe, B.; Young, P. R. Residues in Domain III of the Dengue Virus Envelope Glycoprotein Involved in Cell-Surface Glycosaminoglycan Binding. *J. Gen Virol.* **2012**, *93*, 72-82.

(14) Jorgensen, W. L.; Chandrasekhar, J.; Madura, J. D.; Impey, R. W.; Klein, M. L. Comparison of Simple Potential Functions for Simulating Liquid Water. *J. Chem. Phys.* **1983**, *79*, 926-935.

(15) Darden, T.; York, D.; Pedersen, L. Particle Mesh Ewald: An N· log (N) Method for Ewald sums in Large Systems. *J. Chem. Phys.* **1993**, *98*, 10089-10092.

(16) Ryckaert, J.-P.; Ciccotti, G.; Berendsen, H. J. Numerical Integration of the Cartesian Equations of Motion of a System with Constraints: Molecular D'ynamics of N-alkanes. *J. Comput. Phys.* **1977**, *23*, 327-341.

(17) Roe, D. R.; Cheatham, T. E., III. PTRAJ and CPPTRAJ: Software for Processing and Analysis of Molecular Dynamics Trajectory Data. *J. Chem. Theory Comput.* **2013**, *9*, 3084-3095.

(18) Genheden, S.; Ryde, U. The MM/PBSA and MM/GBSA Methods to Estimate Ligand-Binding Affinities. *Expert Opin. Drug Discov.* **2015**, *10*, 449-461.

9. NMR Spectra of Final GDs: ^{1}H NMR
Glycodendrimer 3a

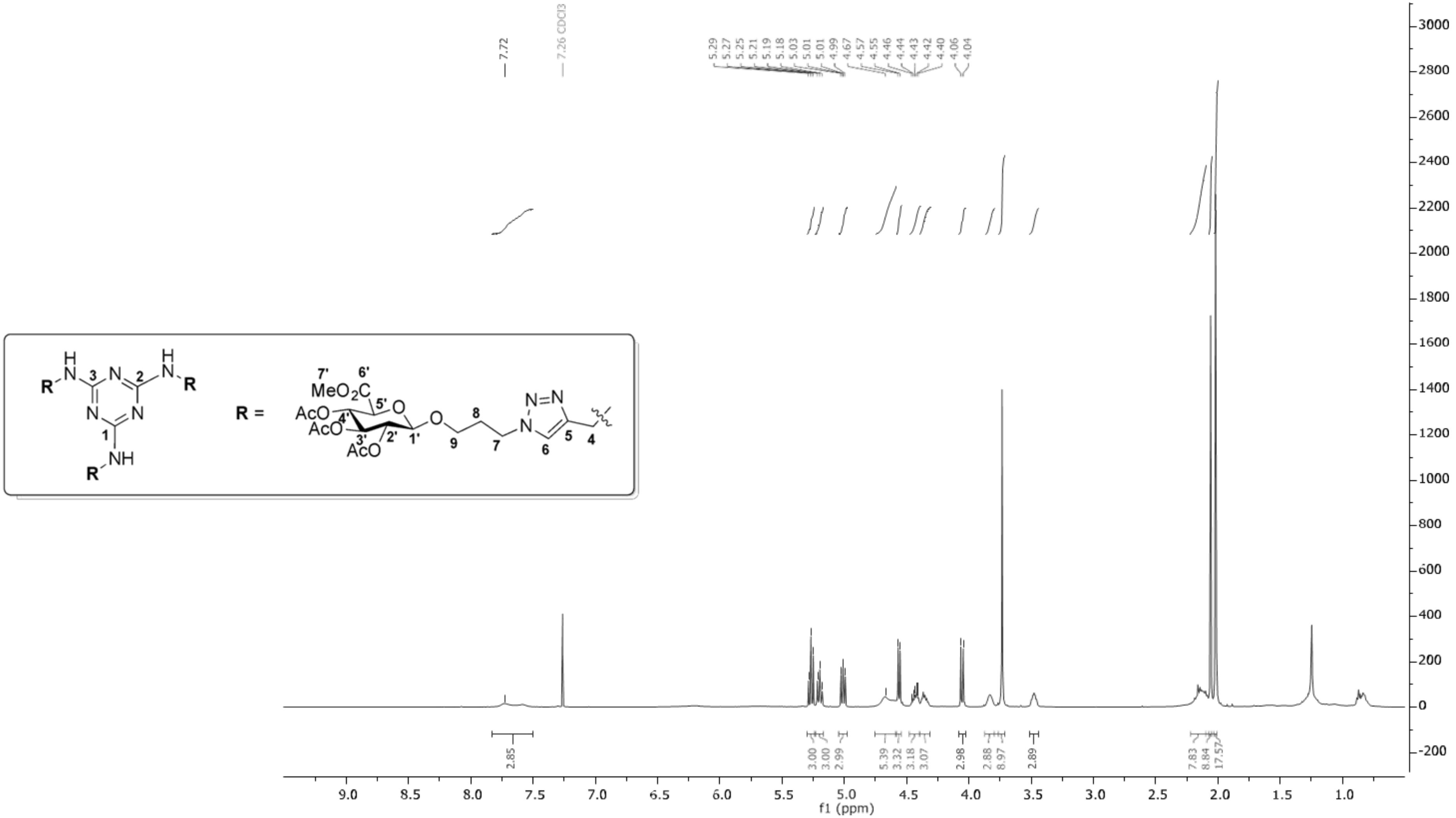

Glycodendrimer 3b

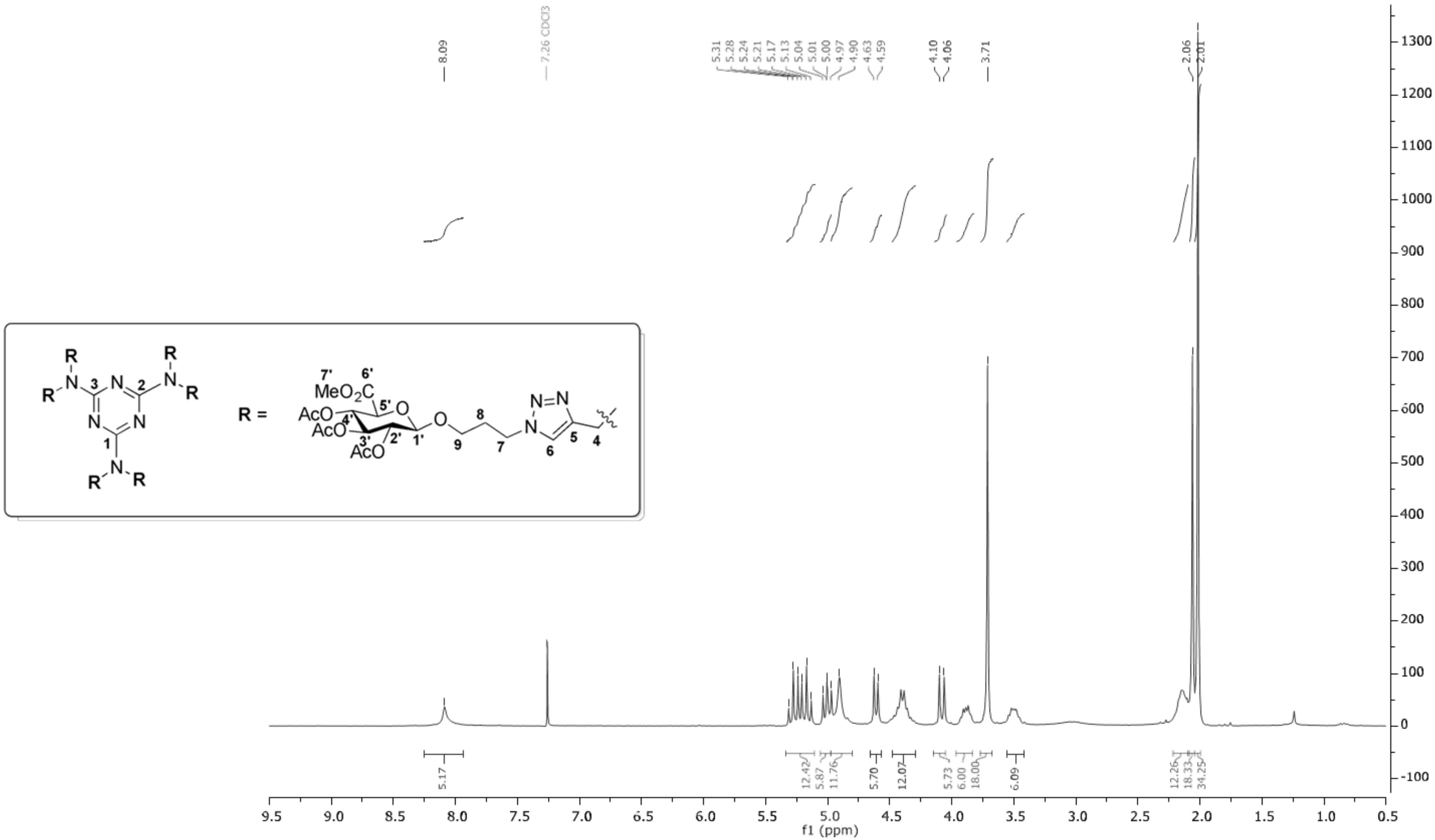

Glycodendrimer 3c

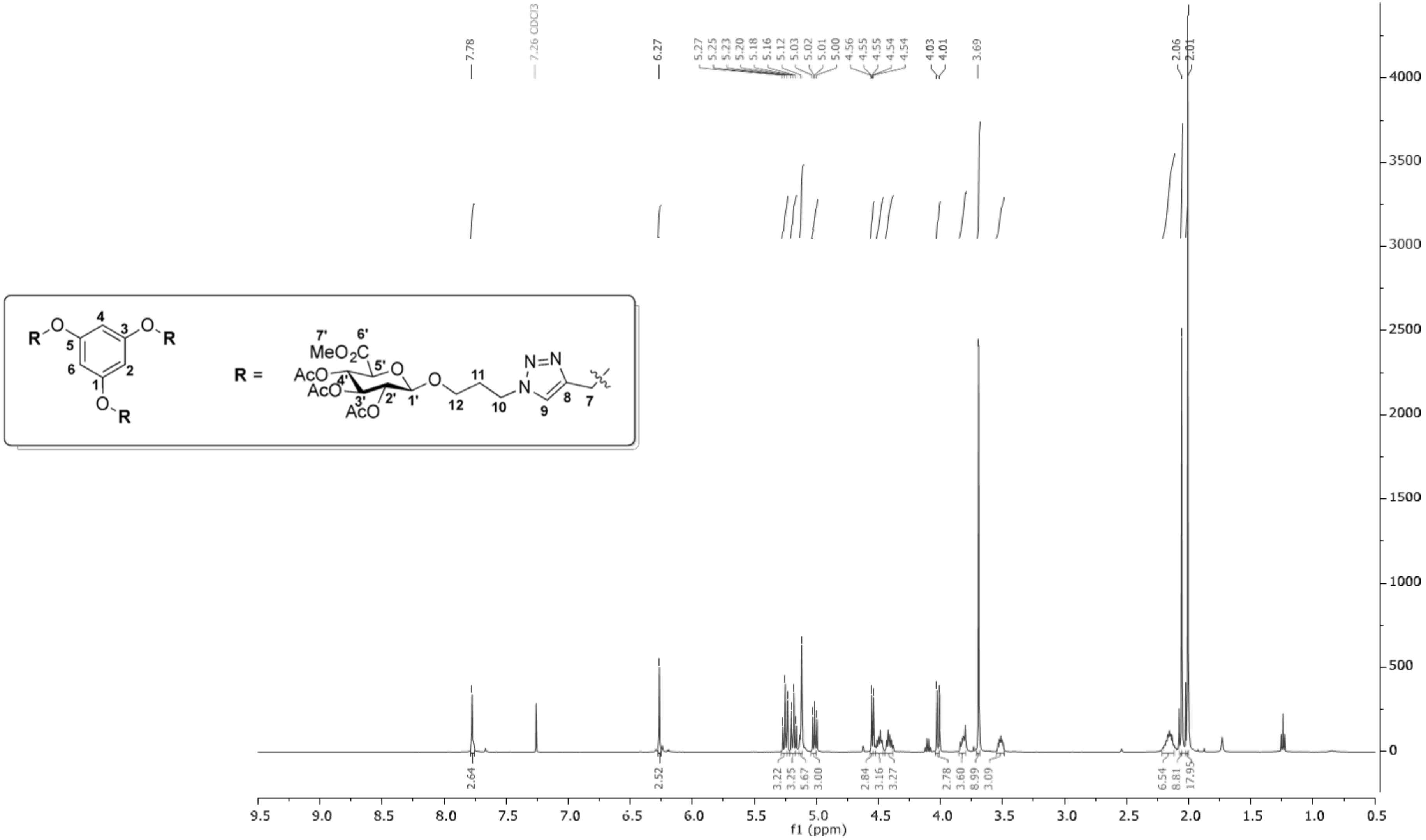

Glycodendrimer 3d

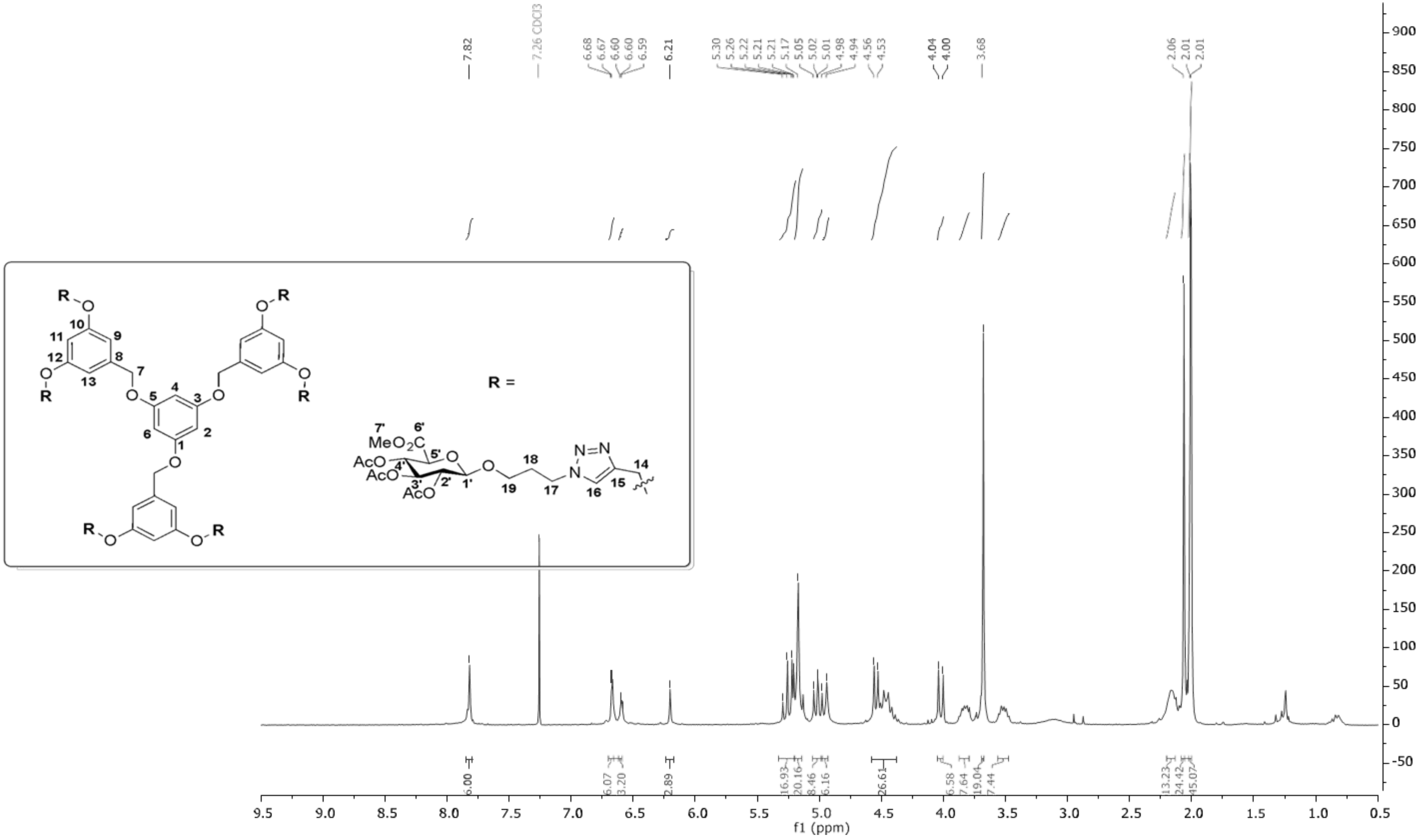

Glycodendrimer 3e

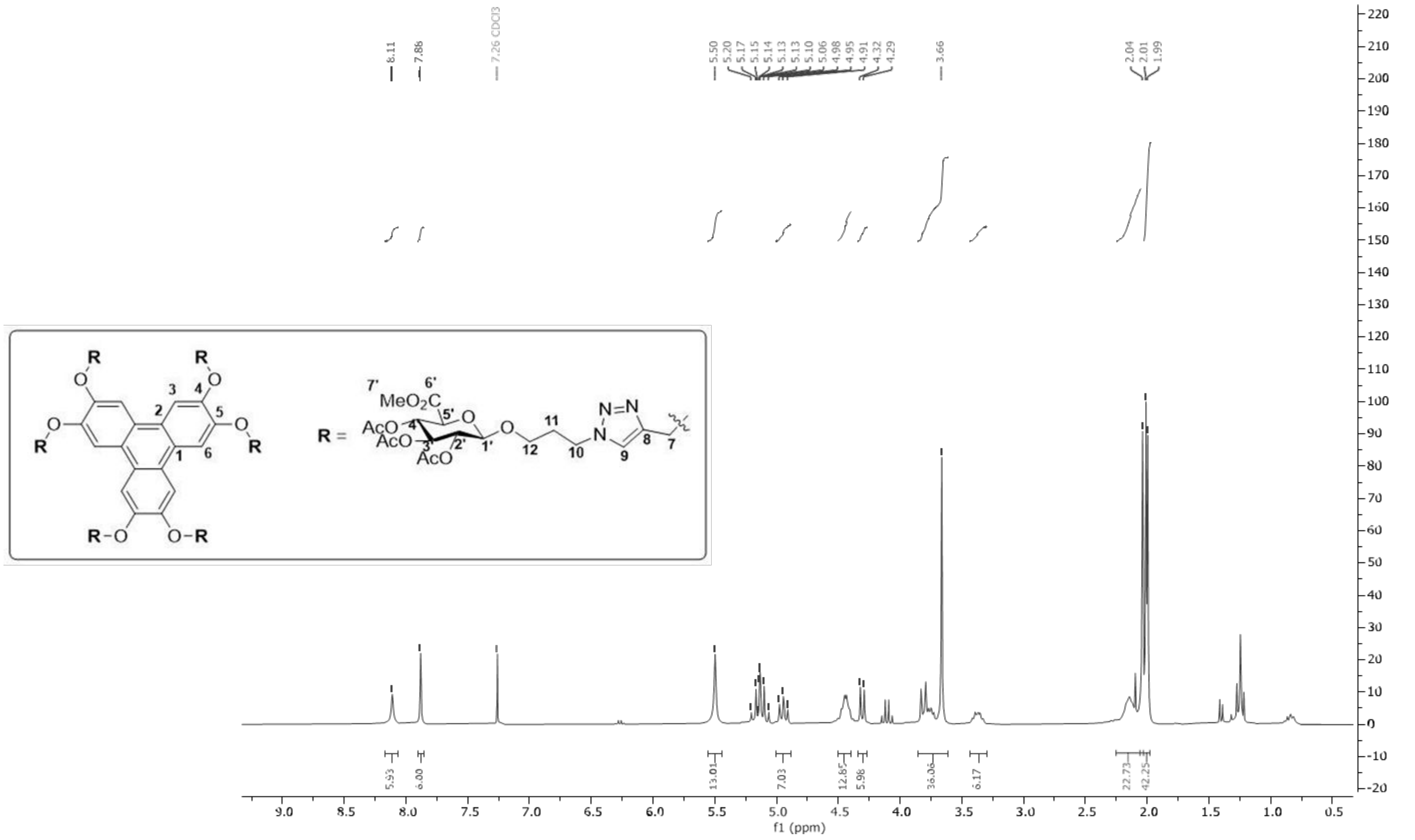

Glycodendrimer 3f

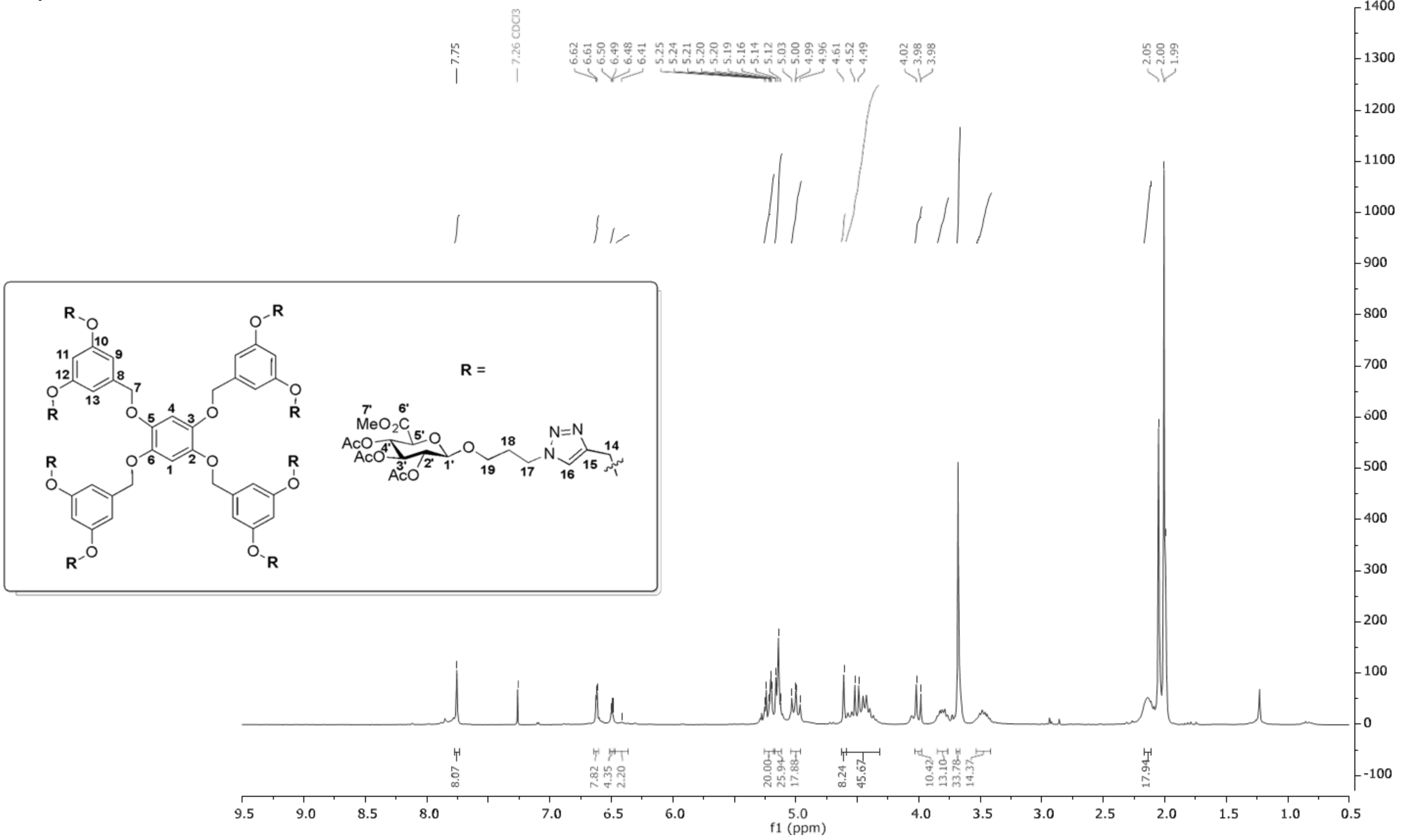

Glycodendrimer 8a

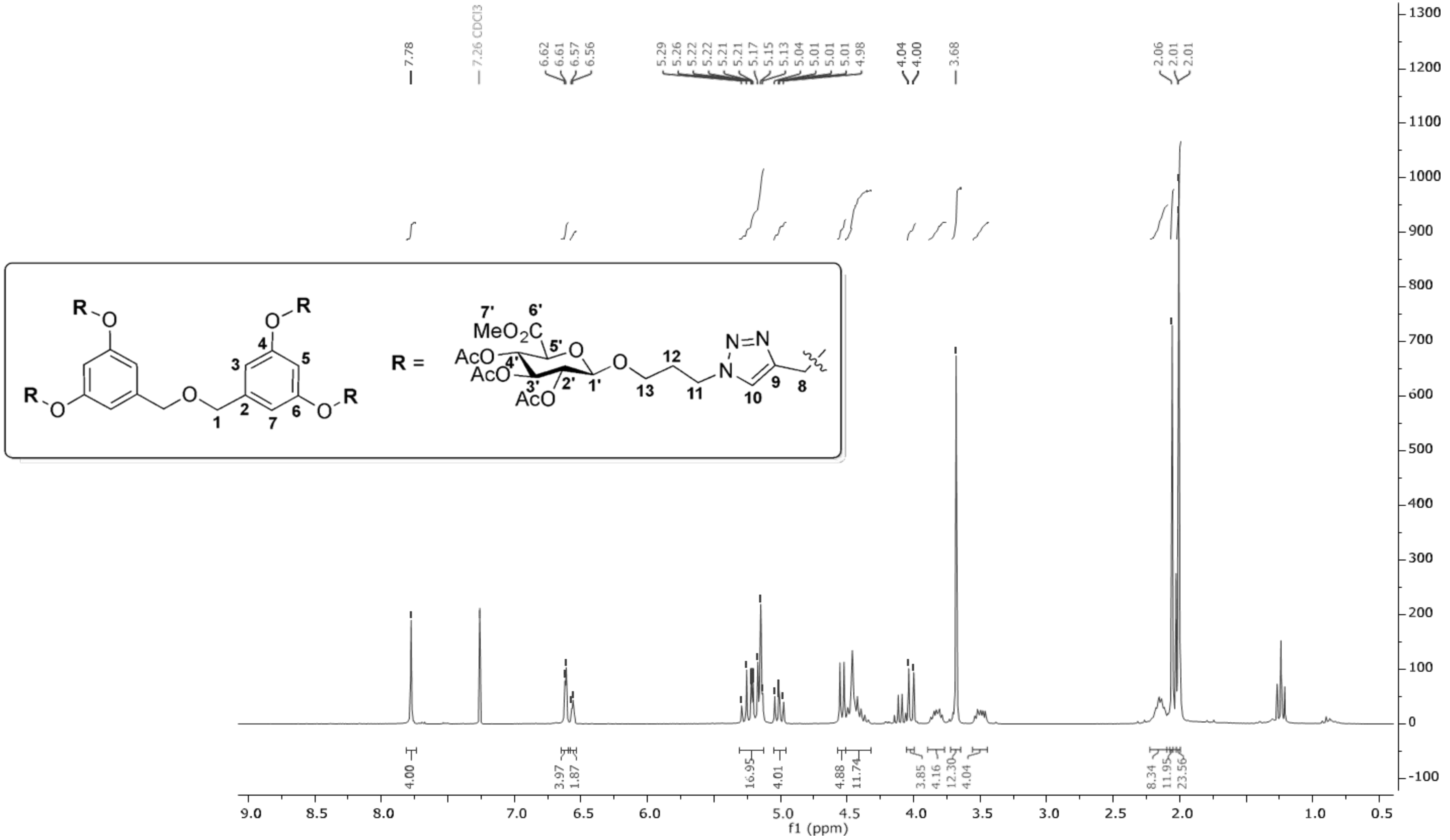

Glycodendrimer 8b

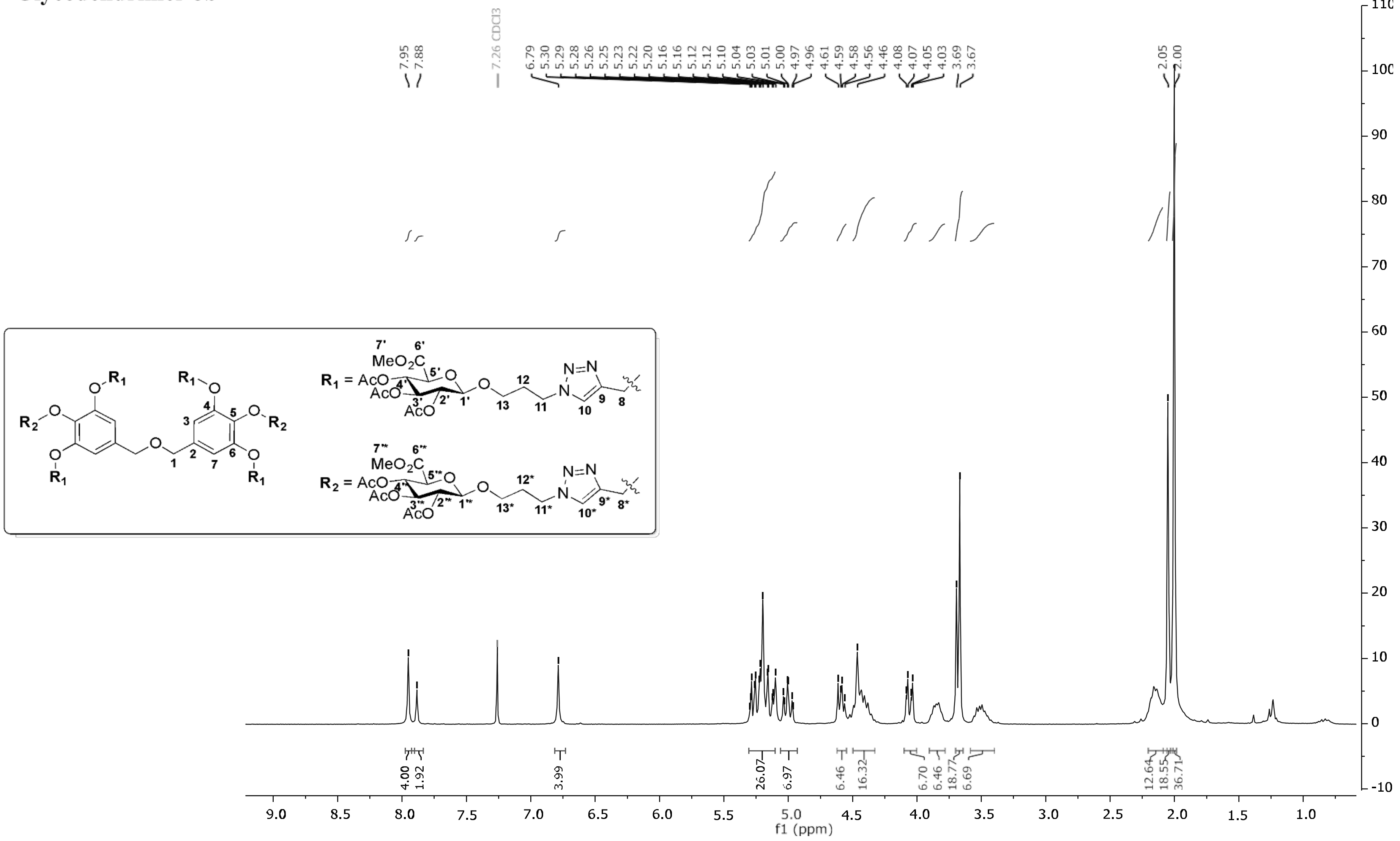

Glycodendrimer 8c

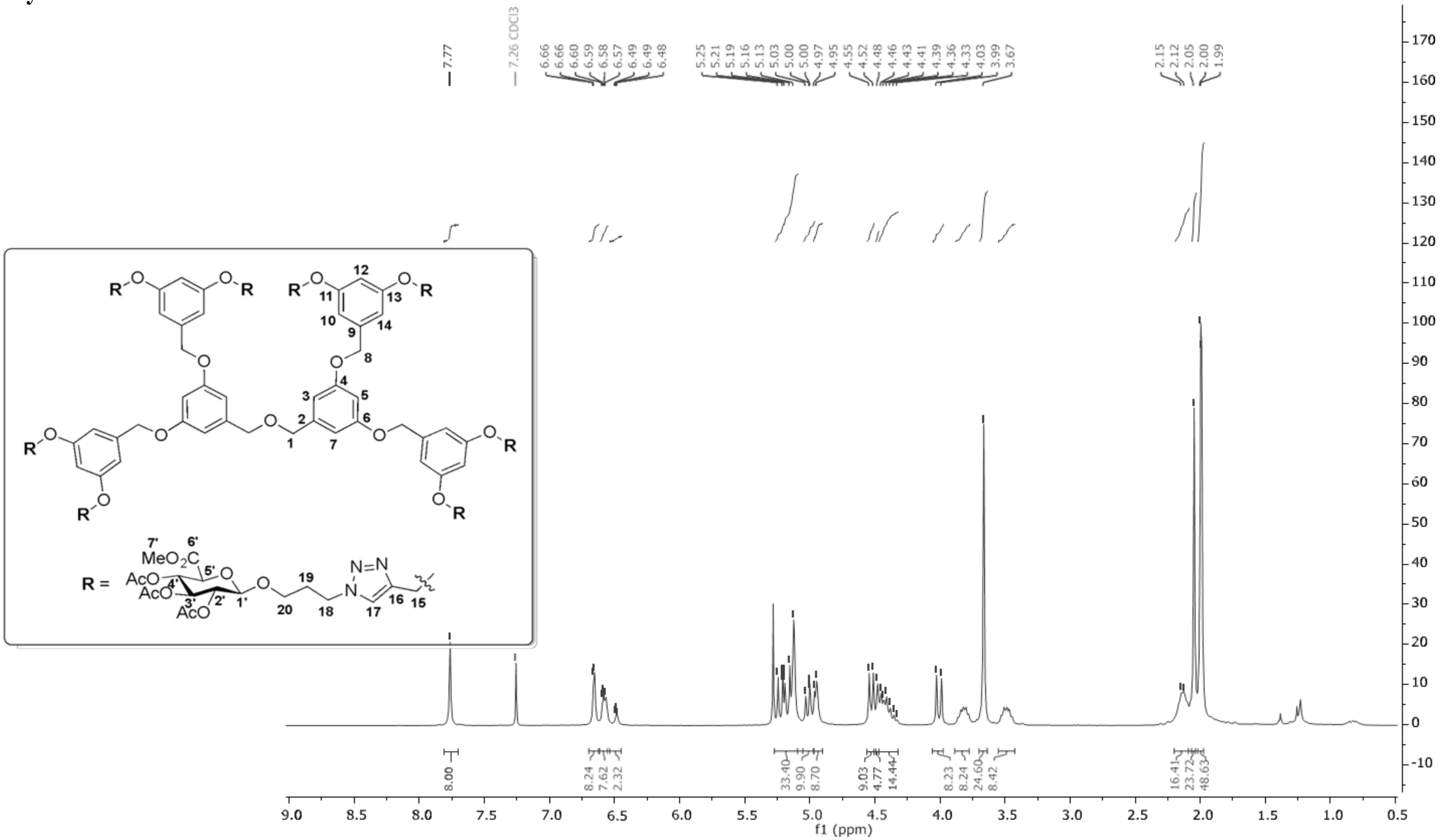

Glycodendrimer 11a

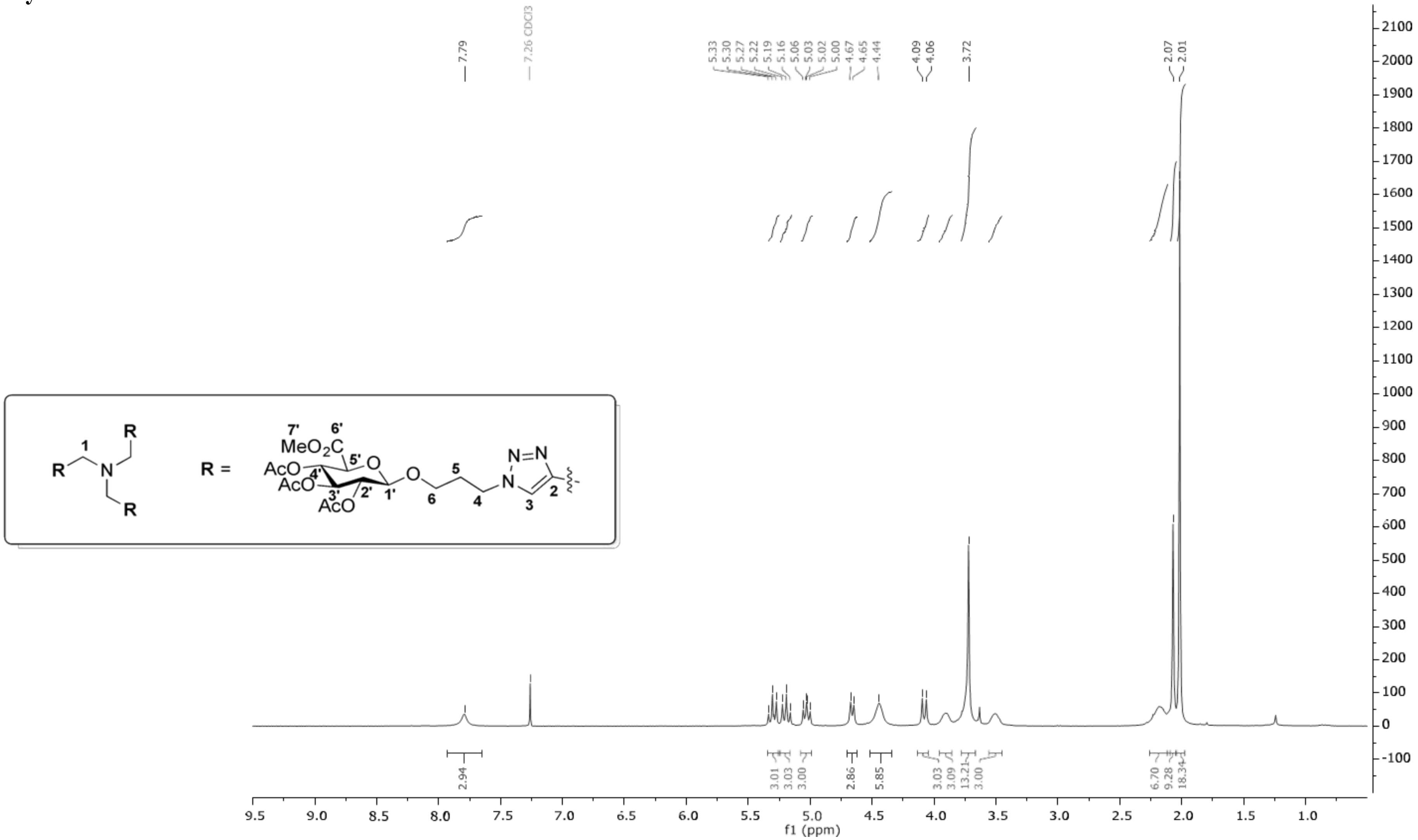

Glycodendrimer 11b

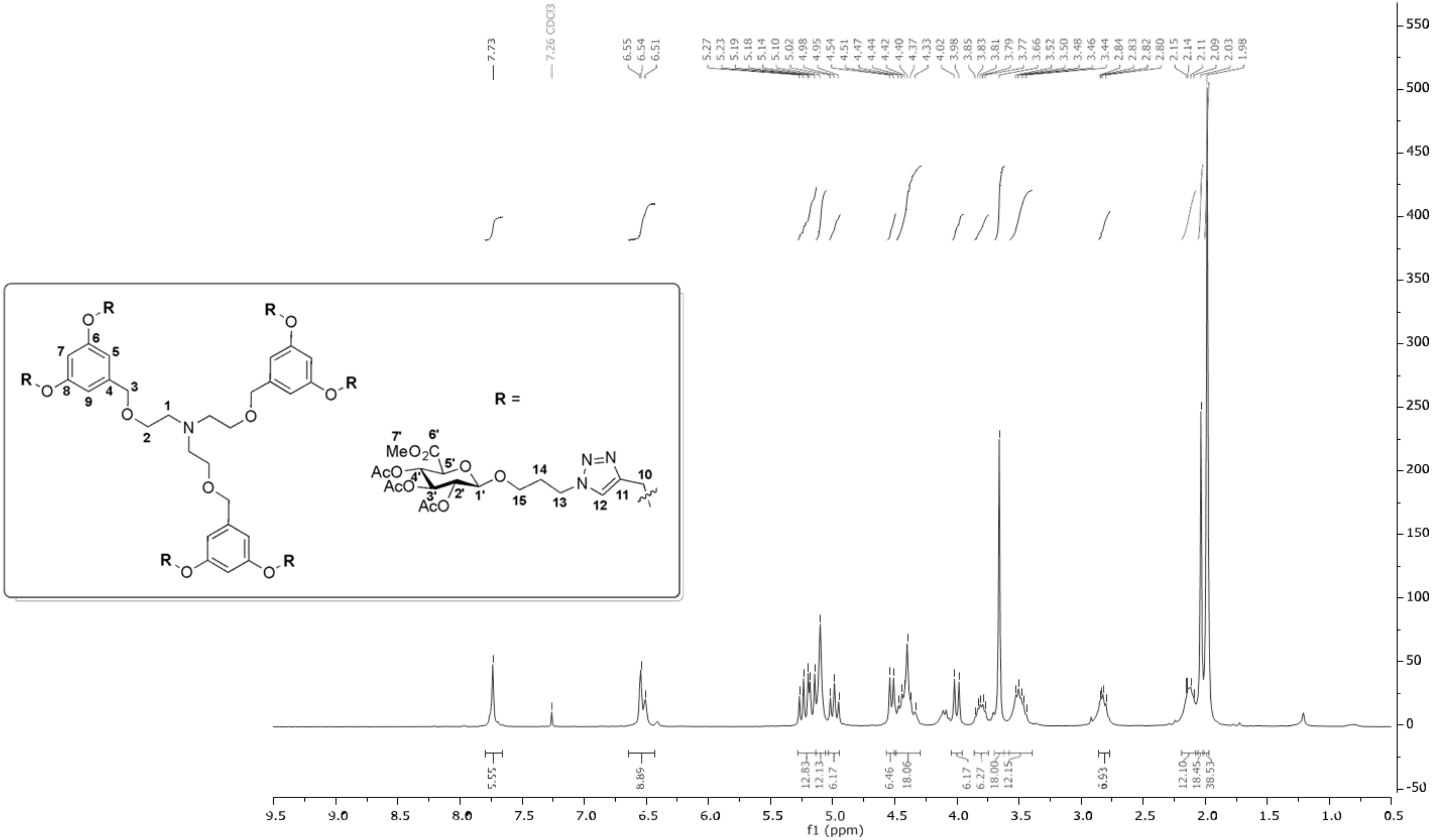

Glycodendrimer 11c

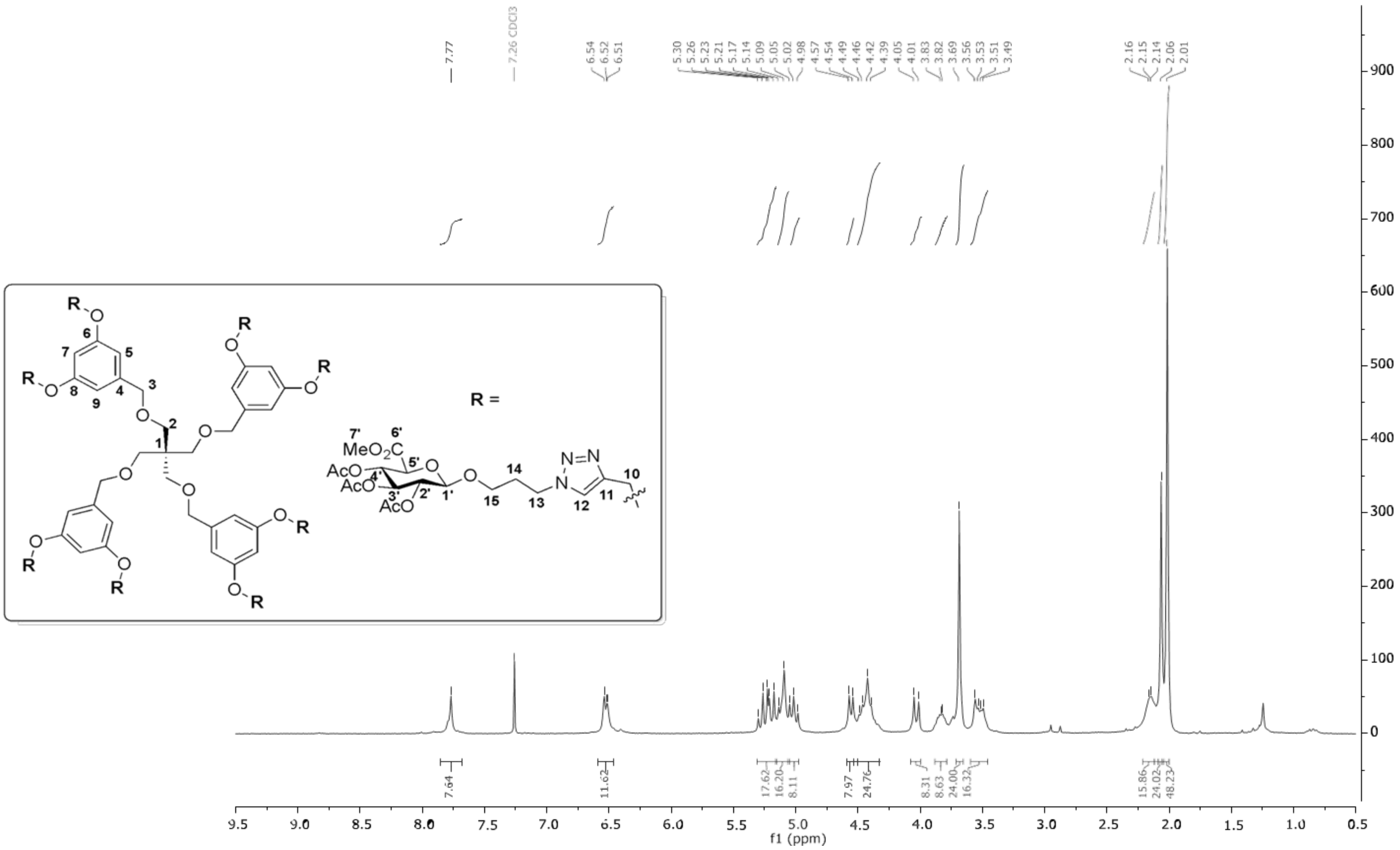

Glycodendrimer 4a

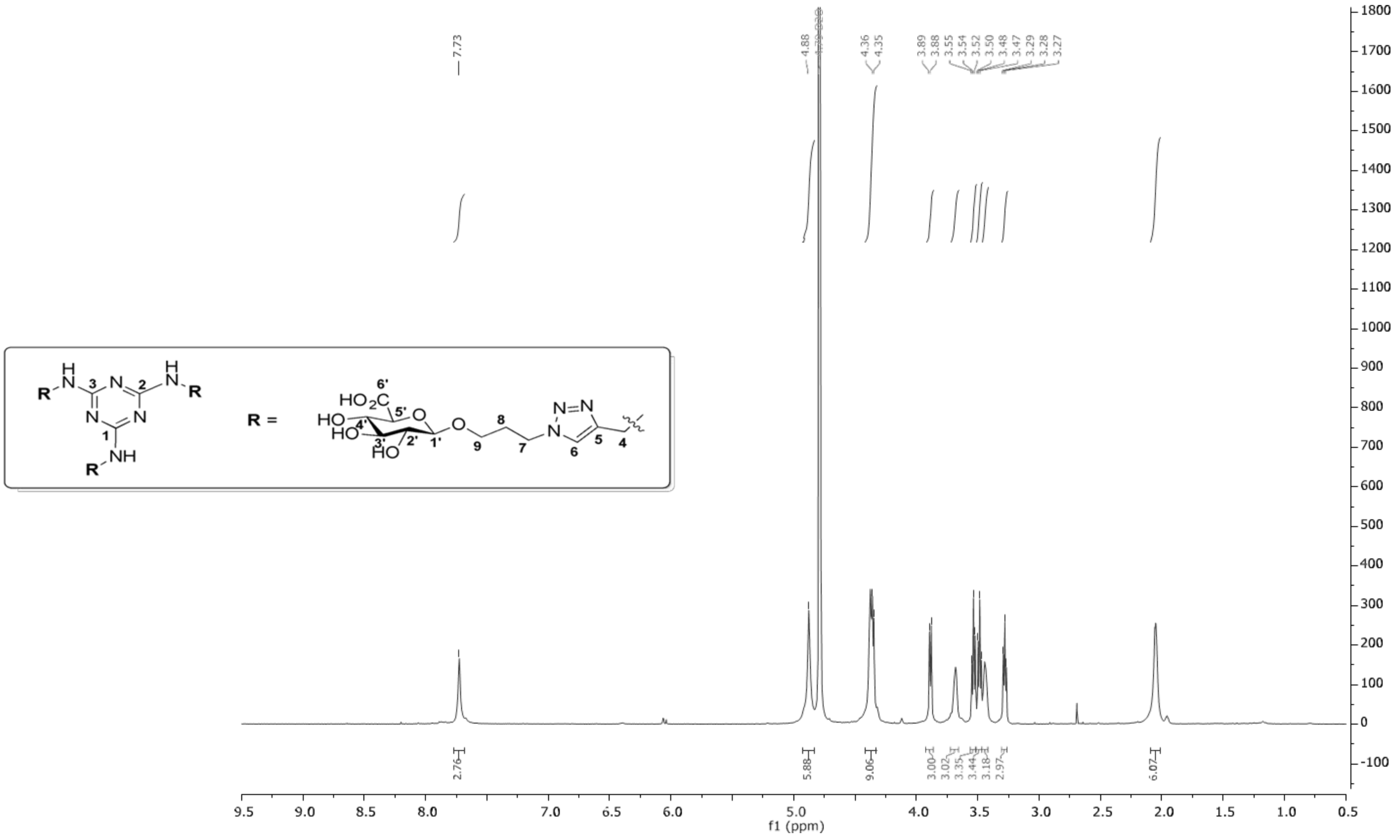

Glycodendrimer 4b

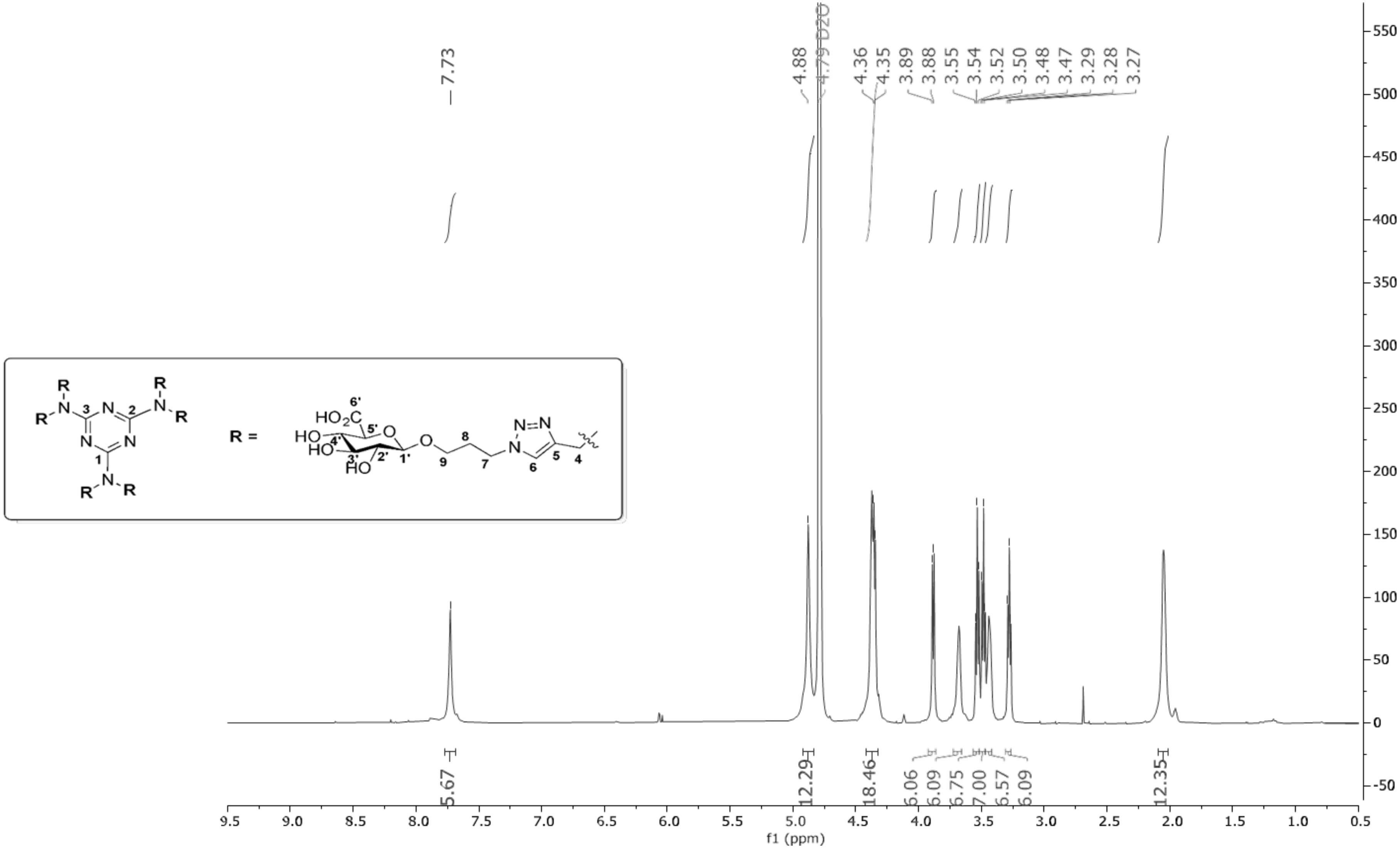

Glycodendrimer 4c

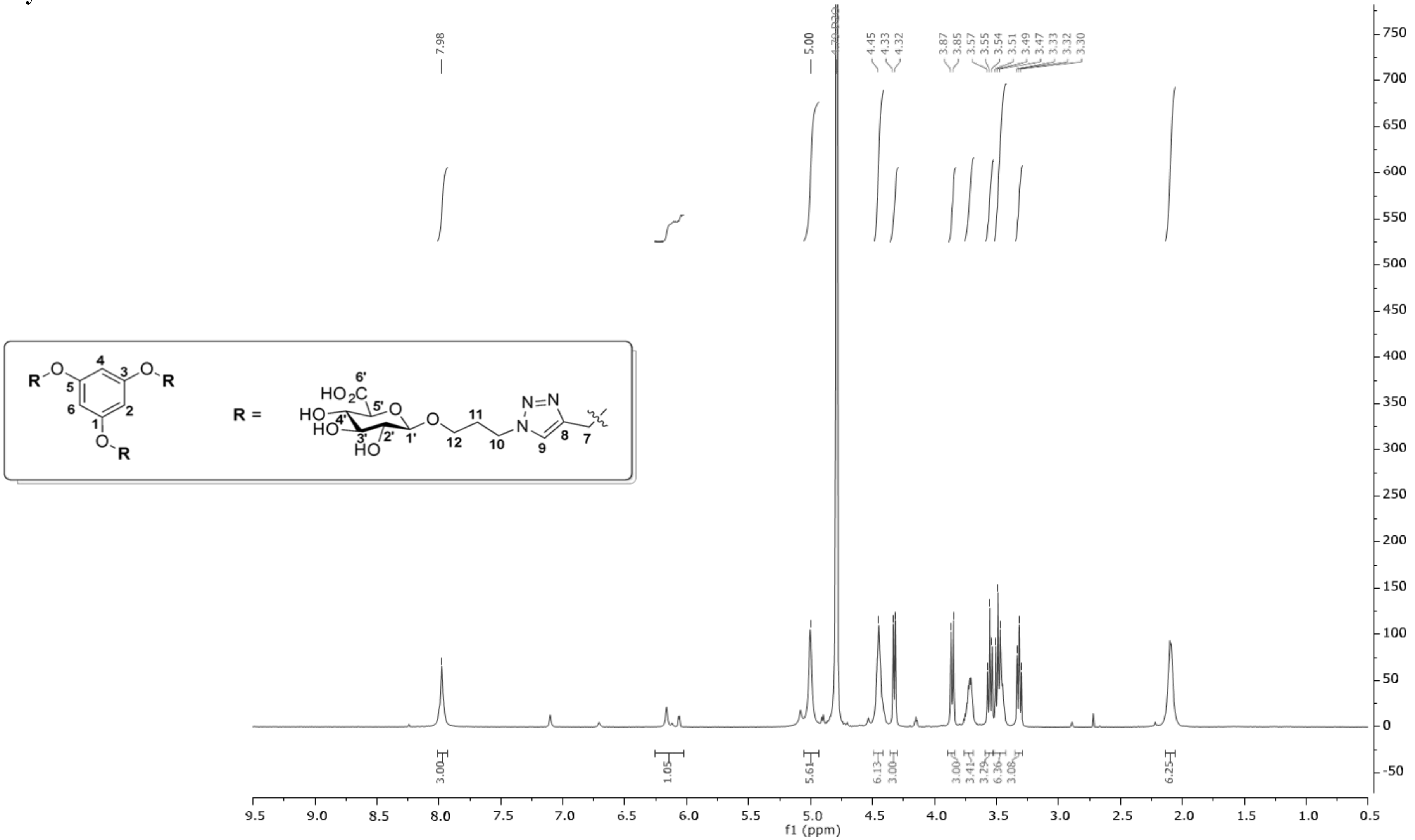

Glycodendrimer 4d

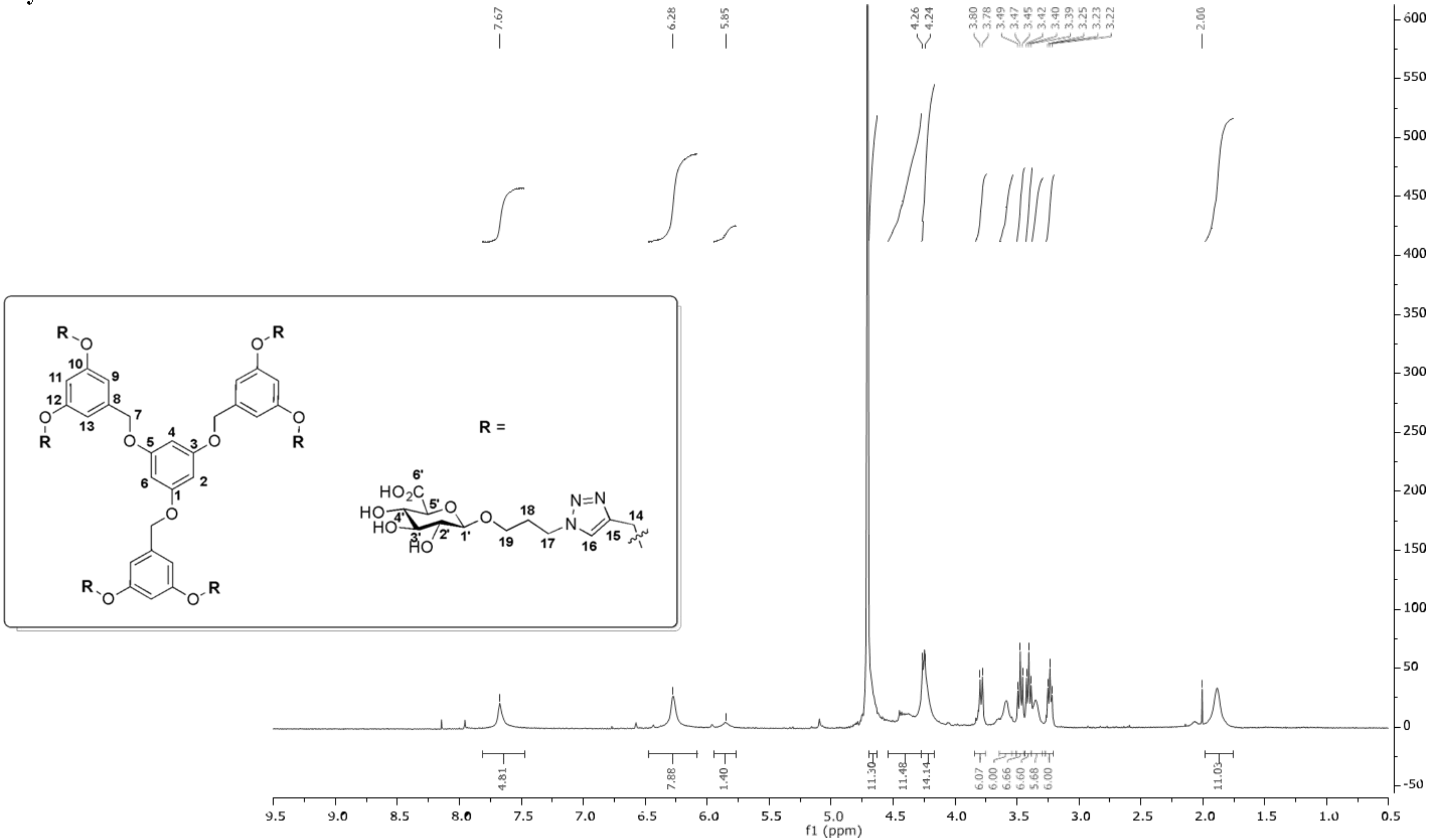

Glycodendrimer 4e

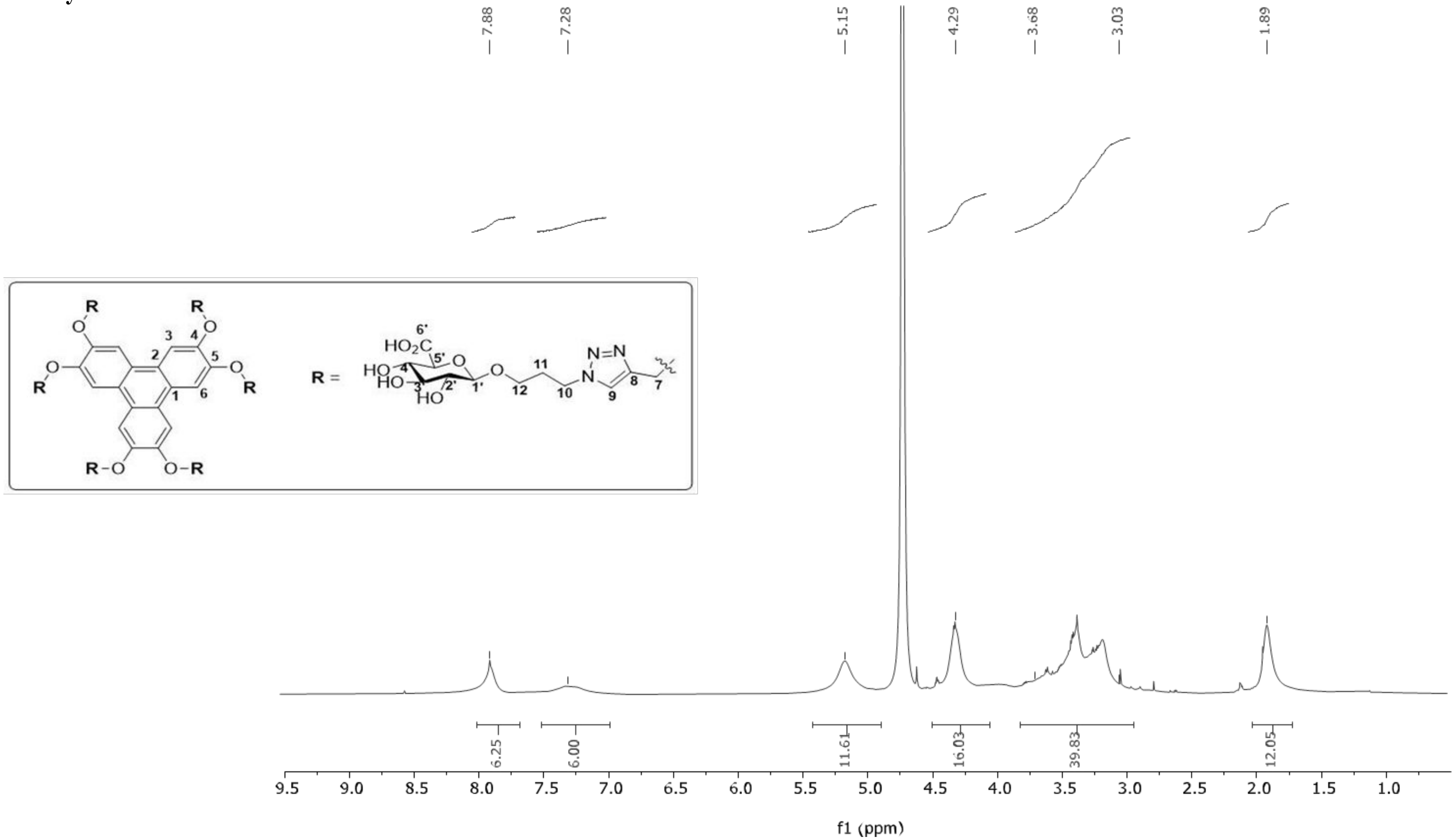

Glycodendrimer 4f

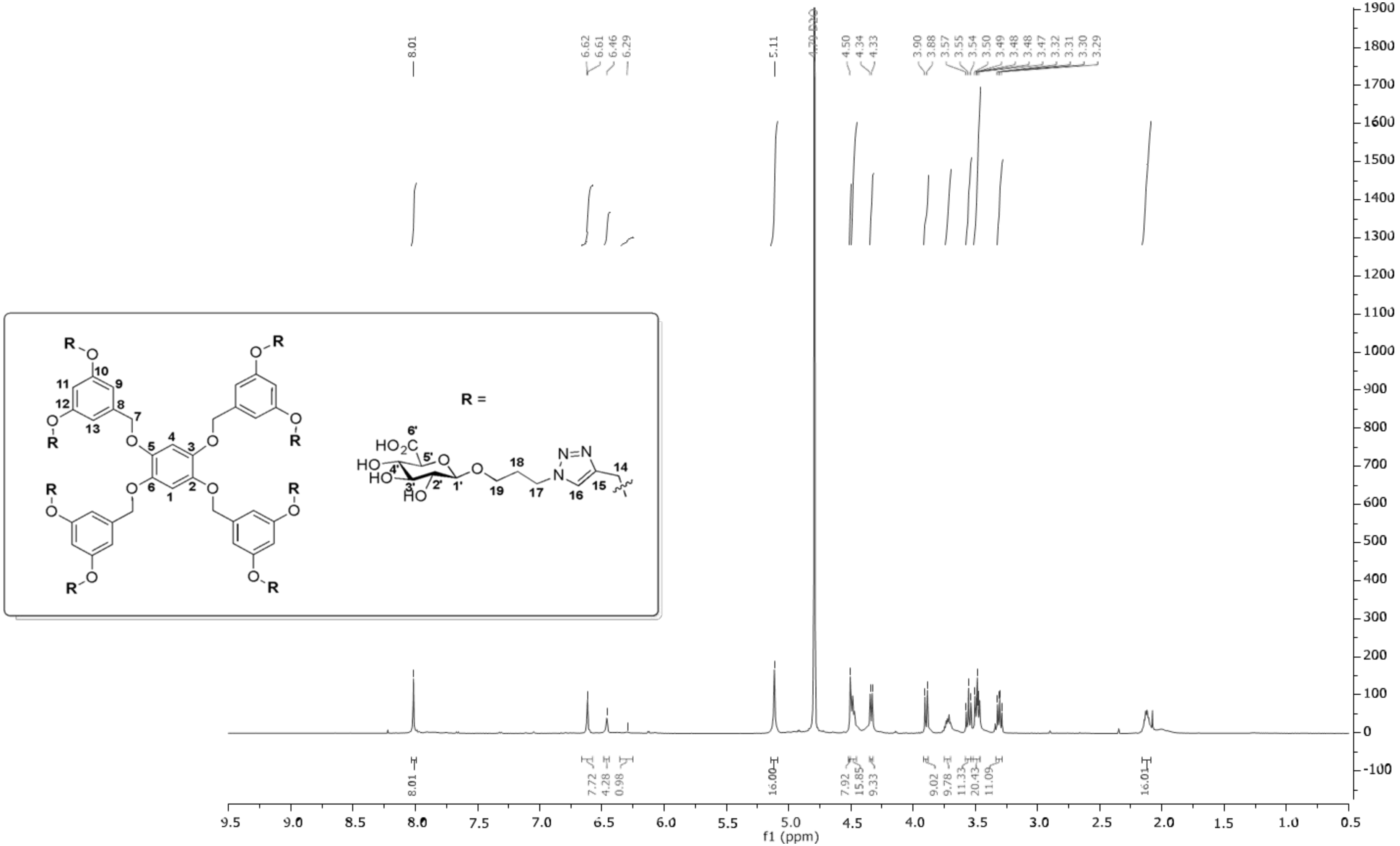

Glycodendrimer 9a

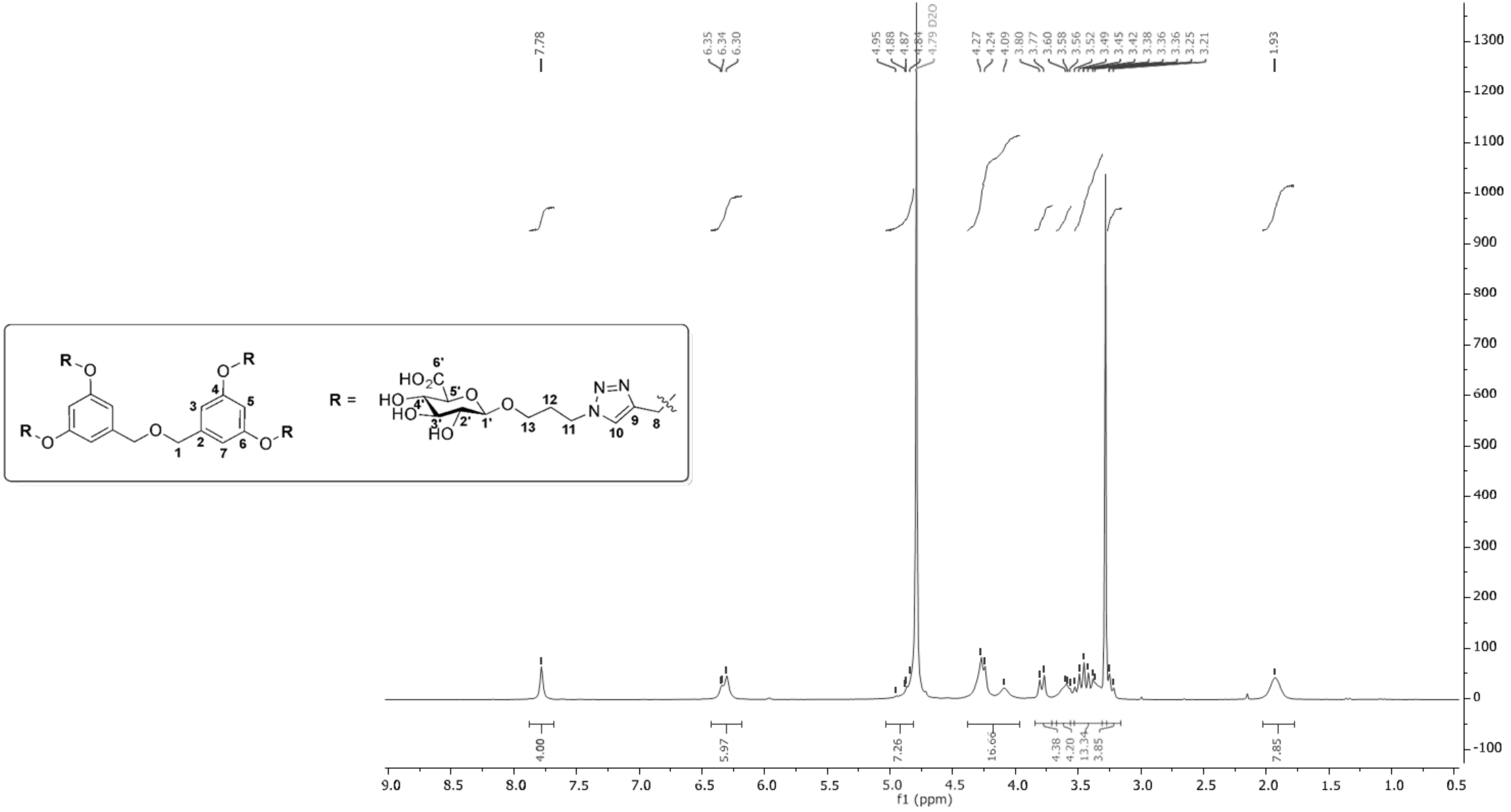

Glycodendrimer 9b

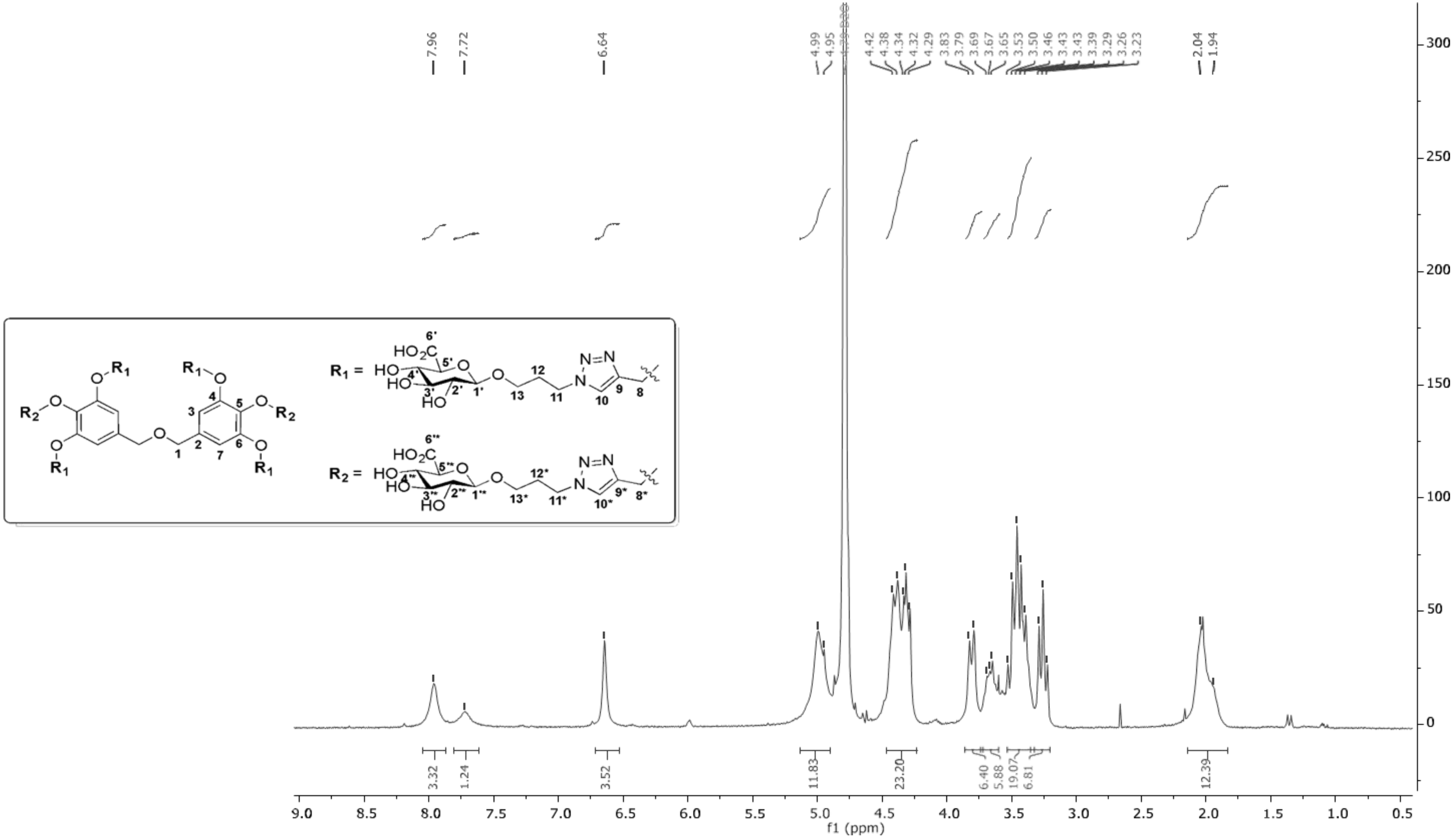

Glycodendrimer 9c

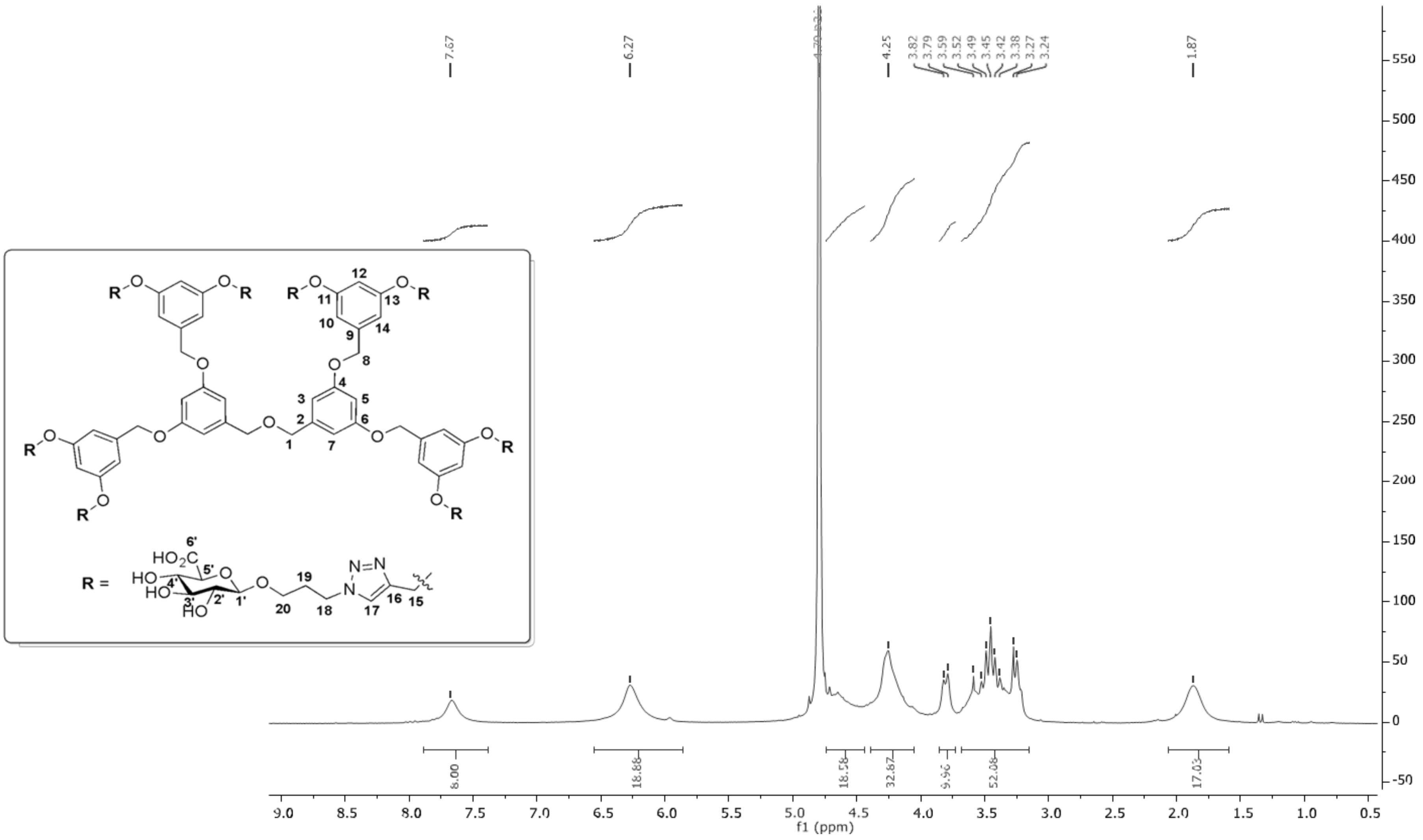

Glycodendrimer 12a

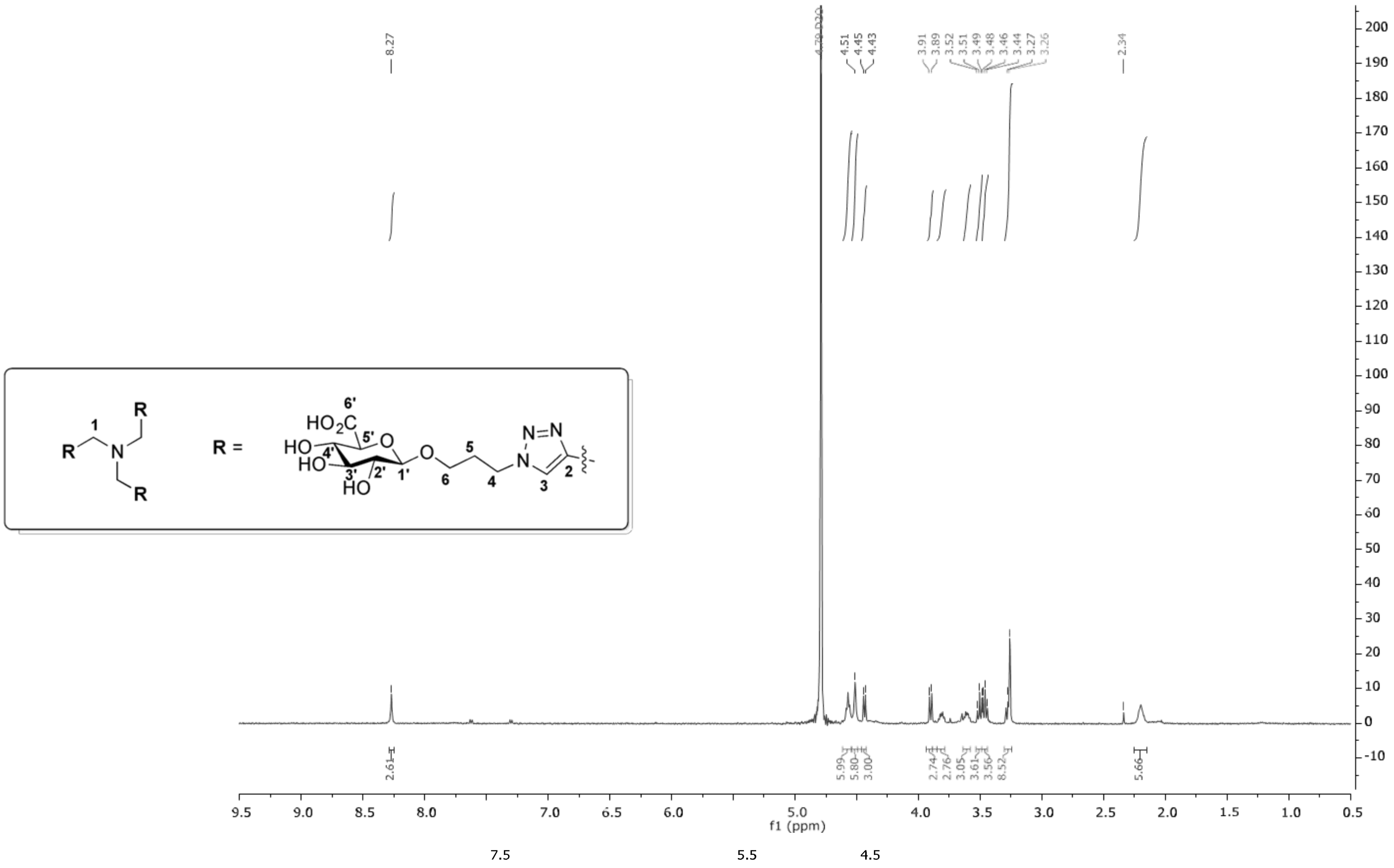

Glycodendrimer 12b

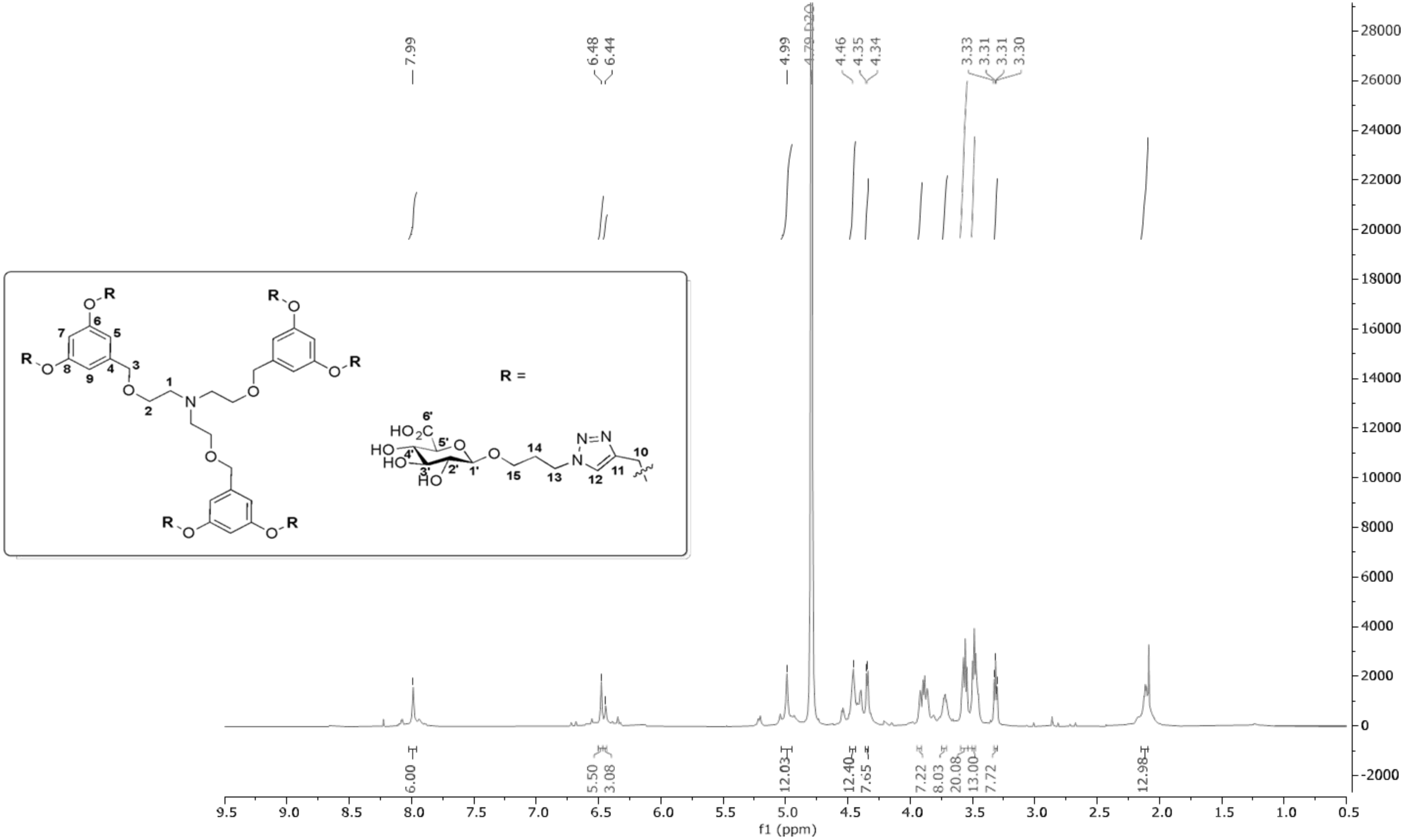

Glycodendrimer 12c

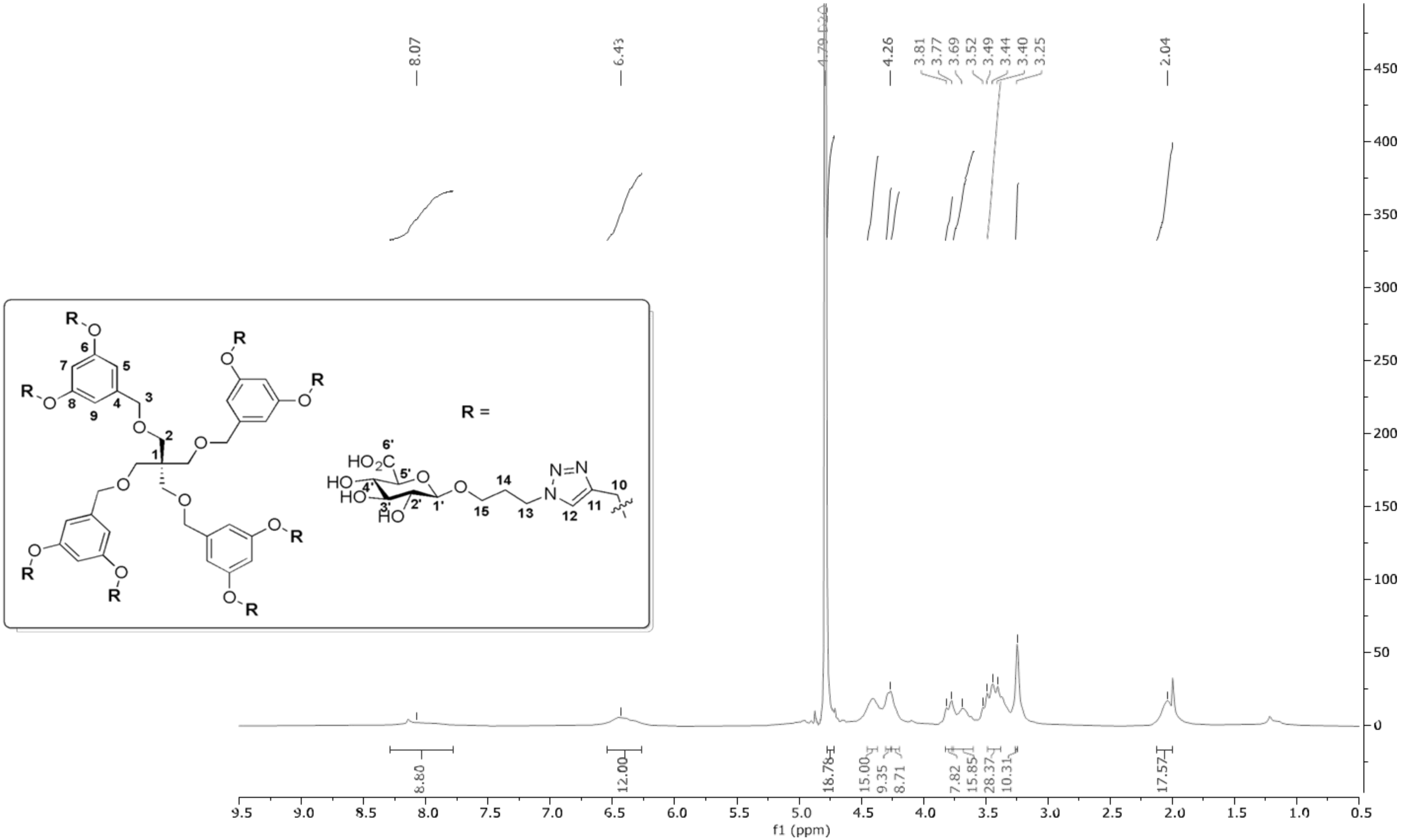

9. NMR Spectra of Final GDs: ^{13}C NMR
Glycodendrimer 3a

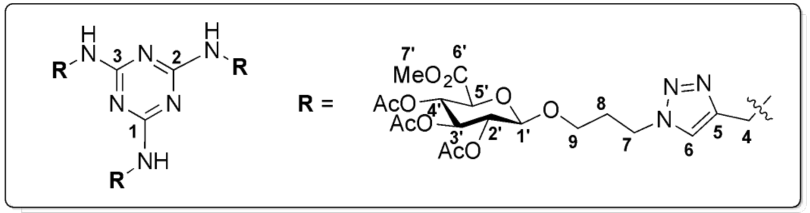

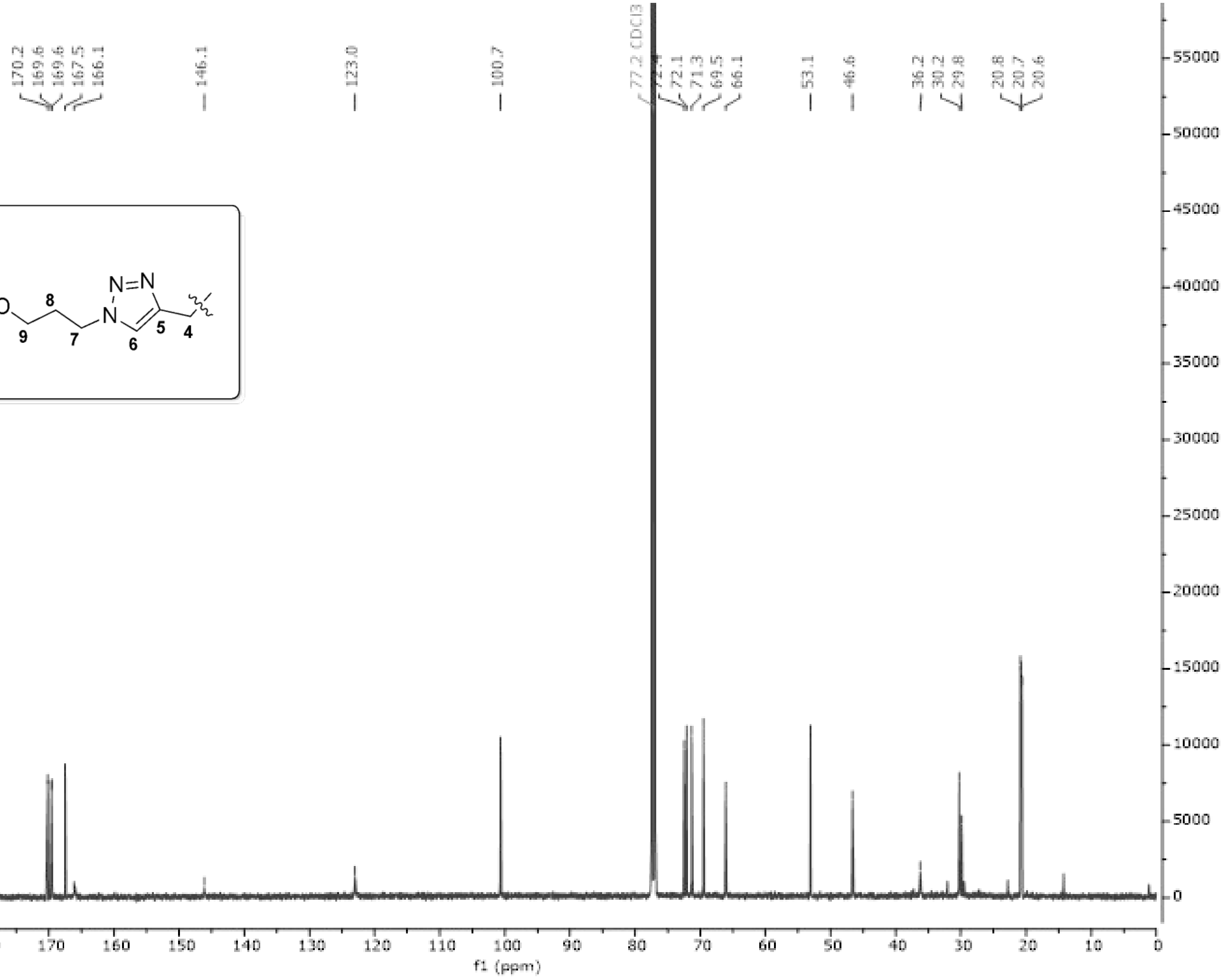

Glycodendrimer 3b

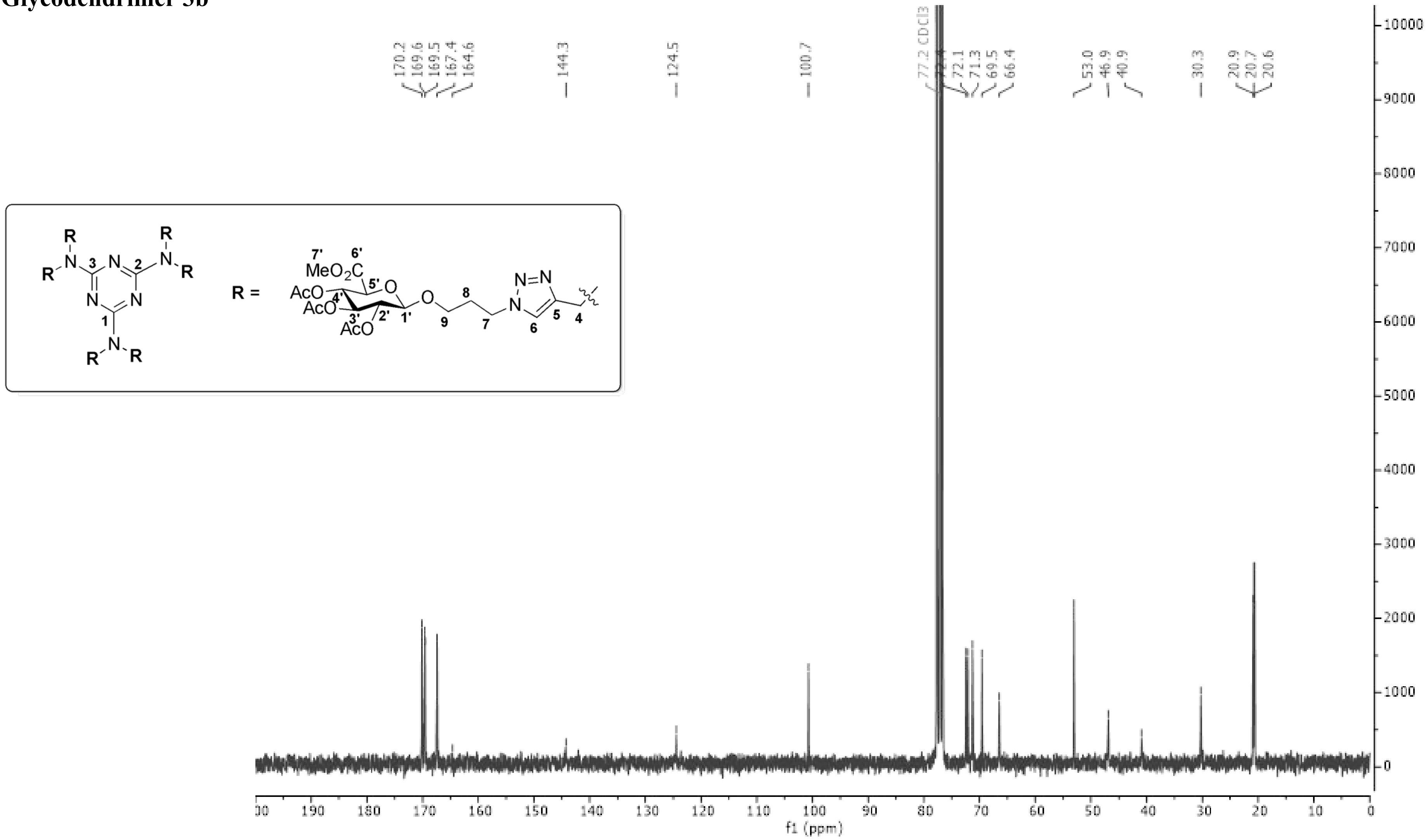

Glycodendrimer 3c

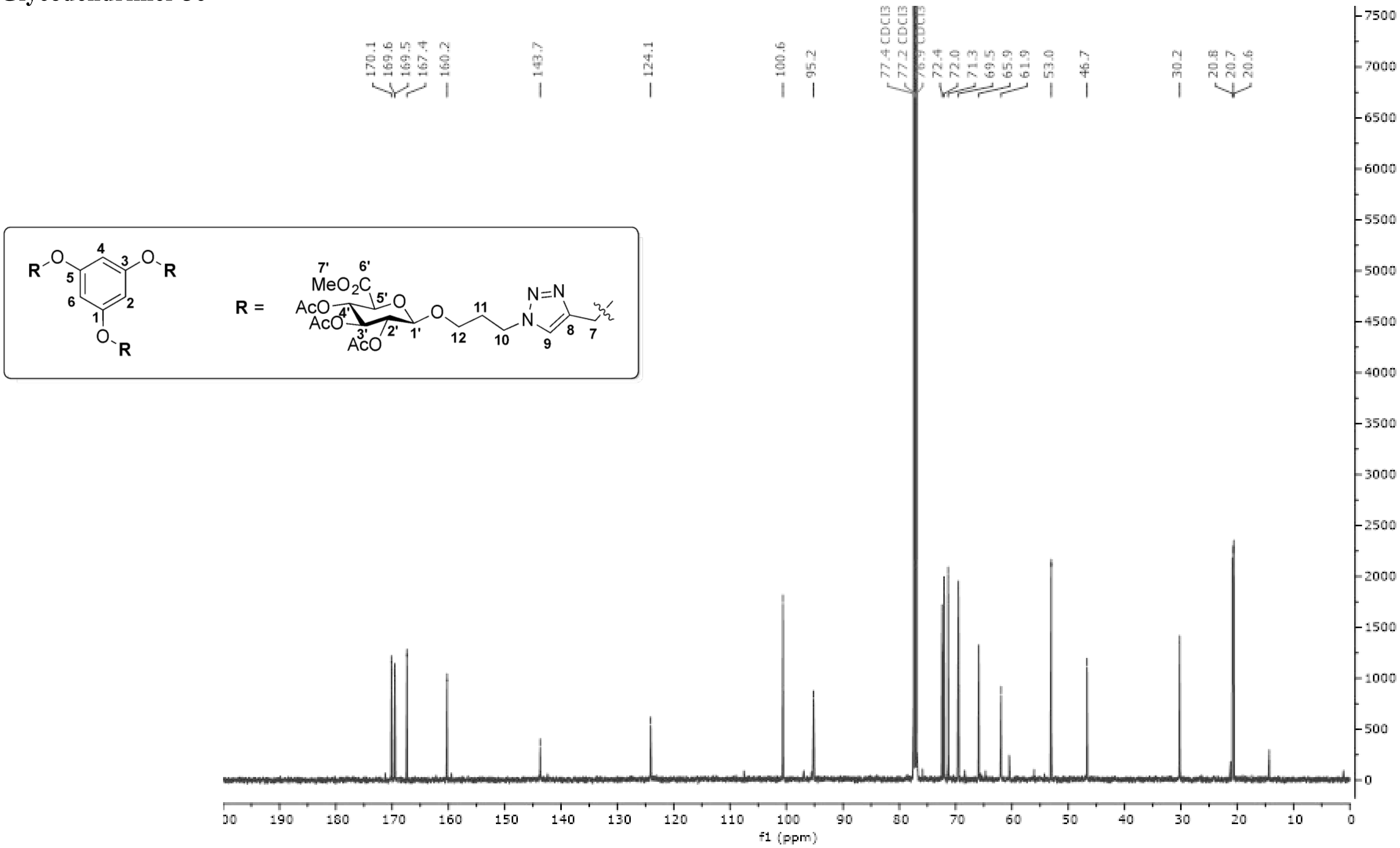

Glycodendrimer 3d

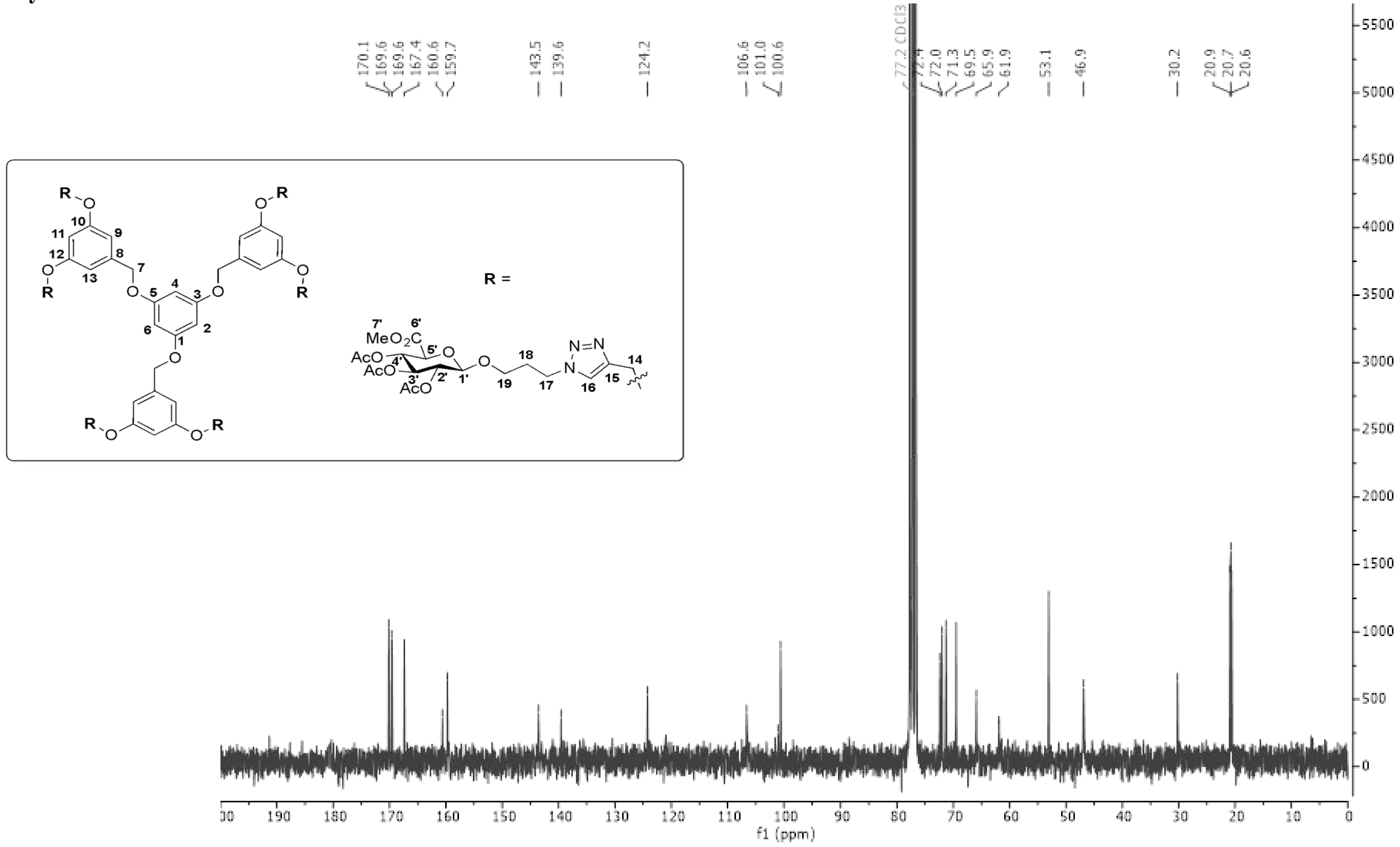

Glycodendrimer 3e

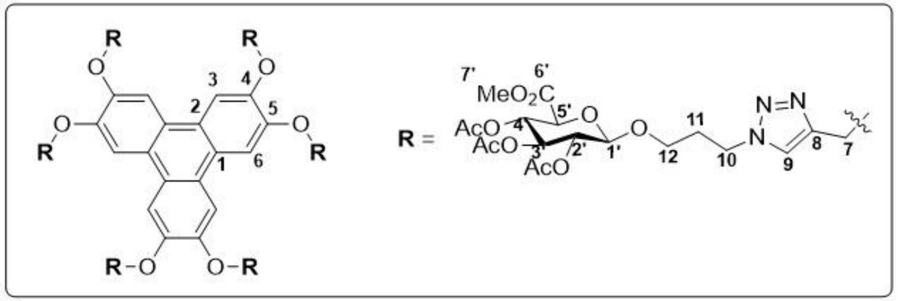

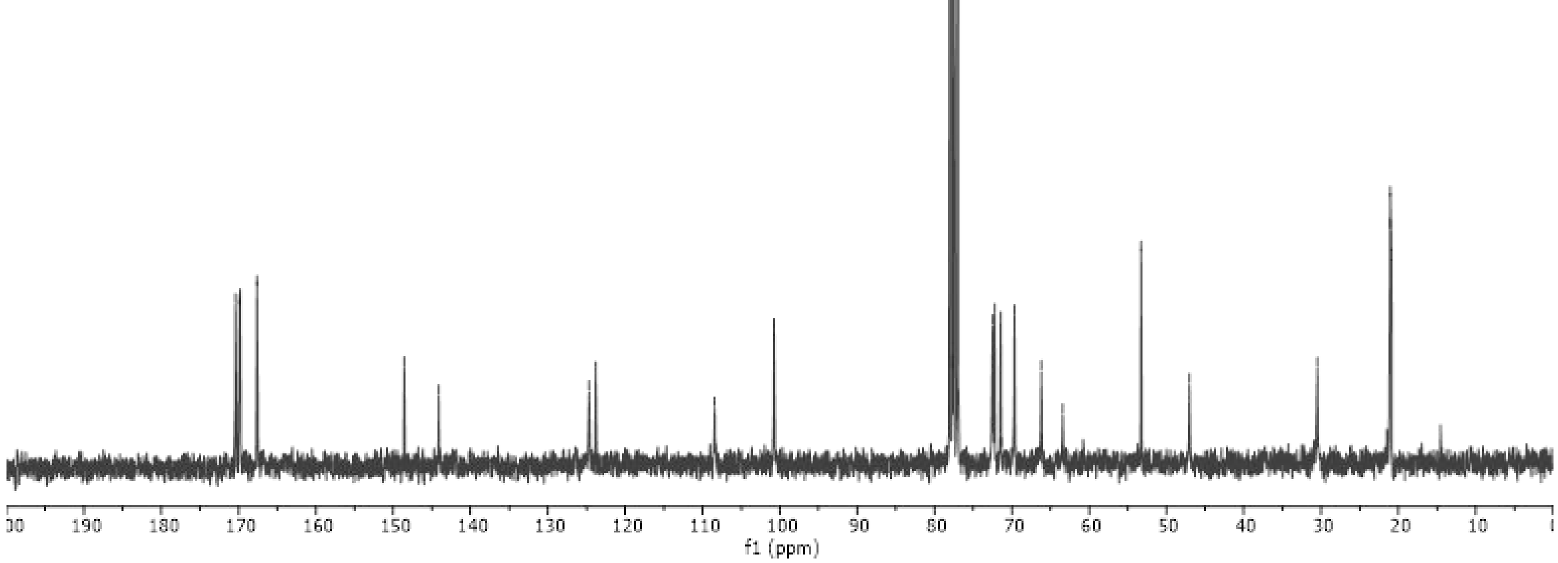

Glycodendrimer 3f

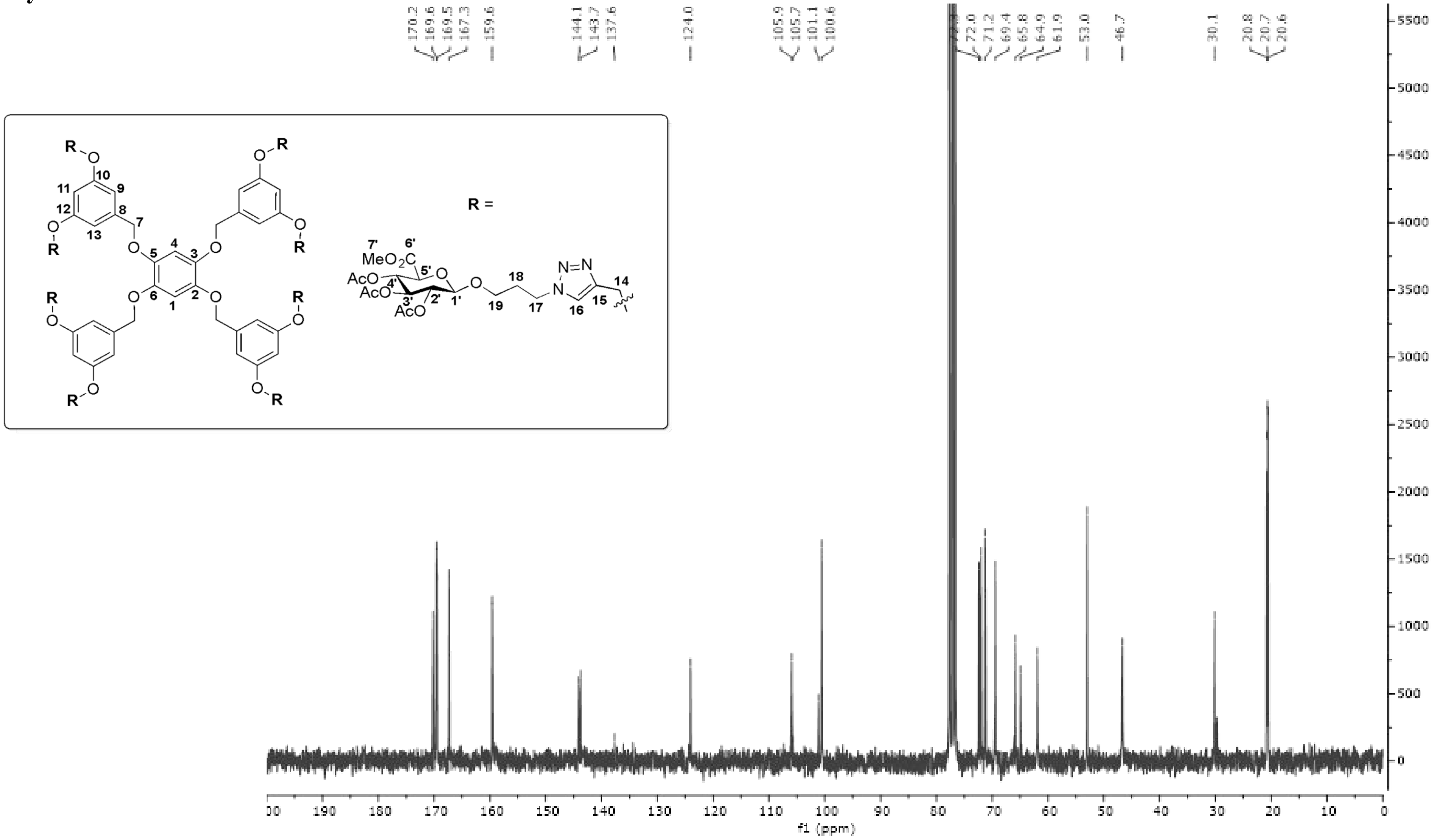

Glycodendrimer 8a

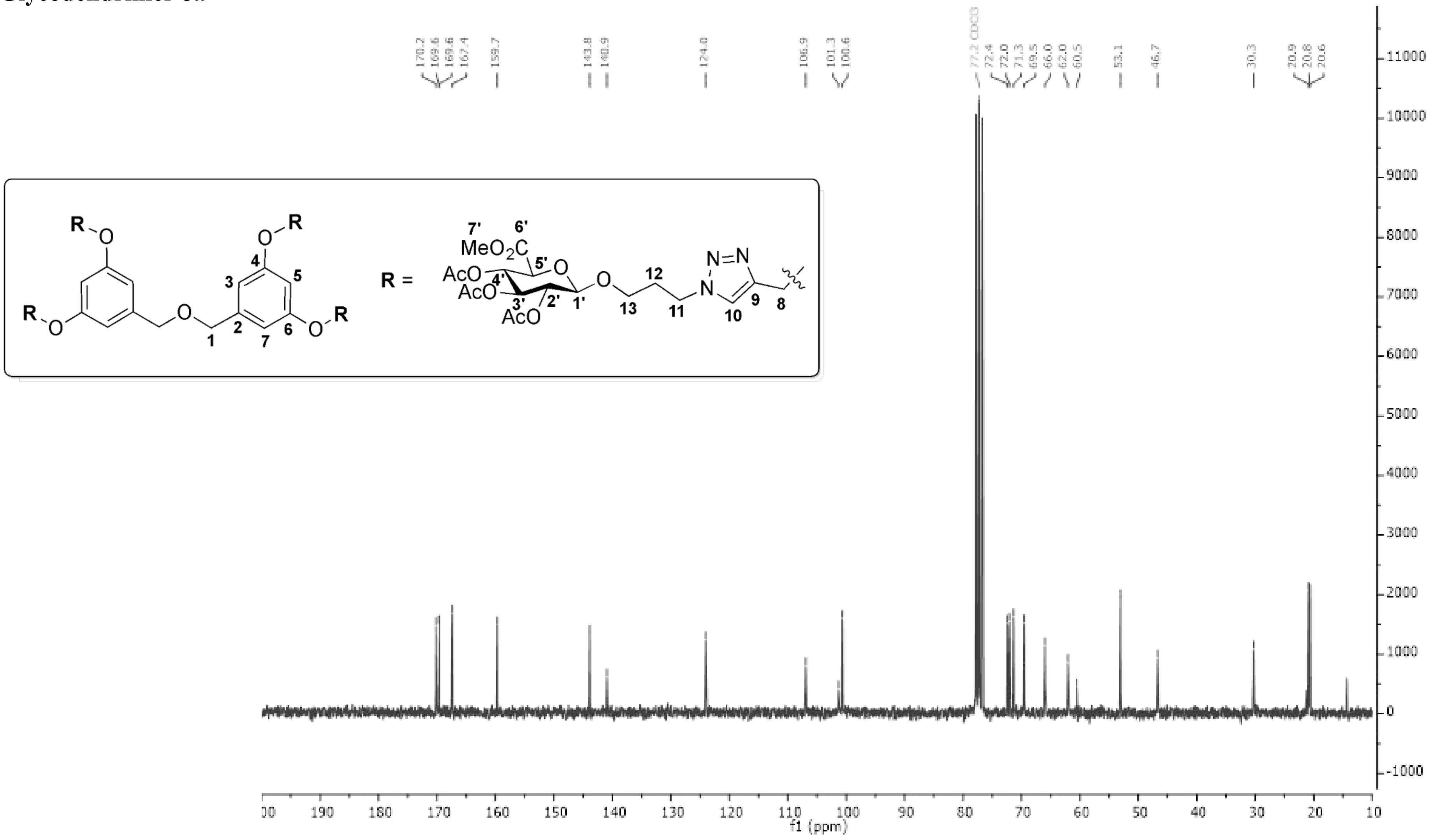

Glycodendrimer 8b

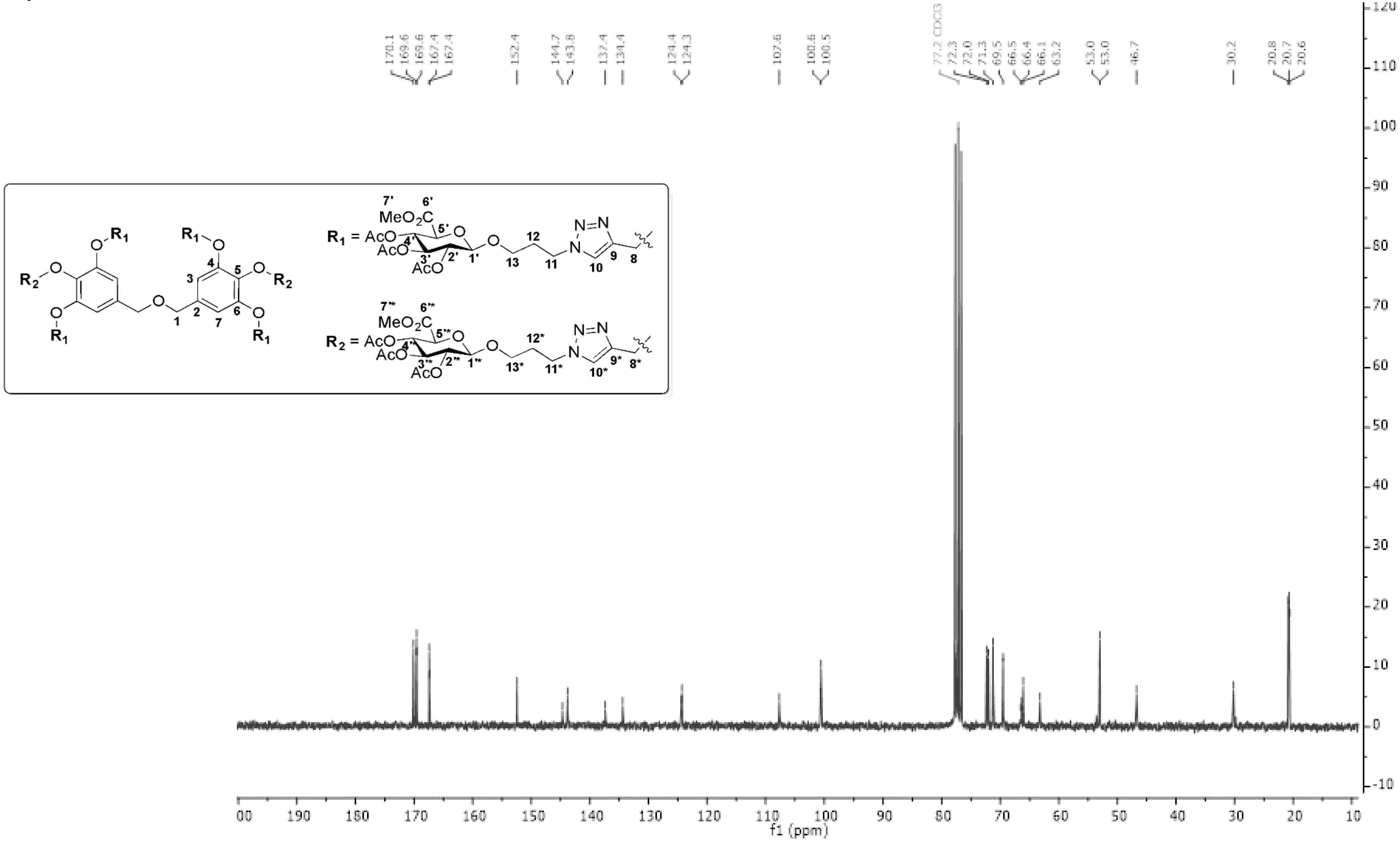

Glycodendrimer 8c

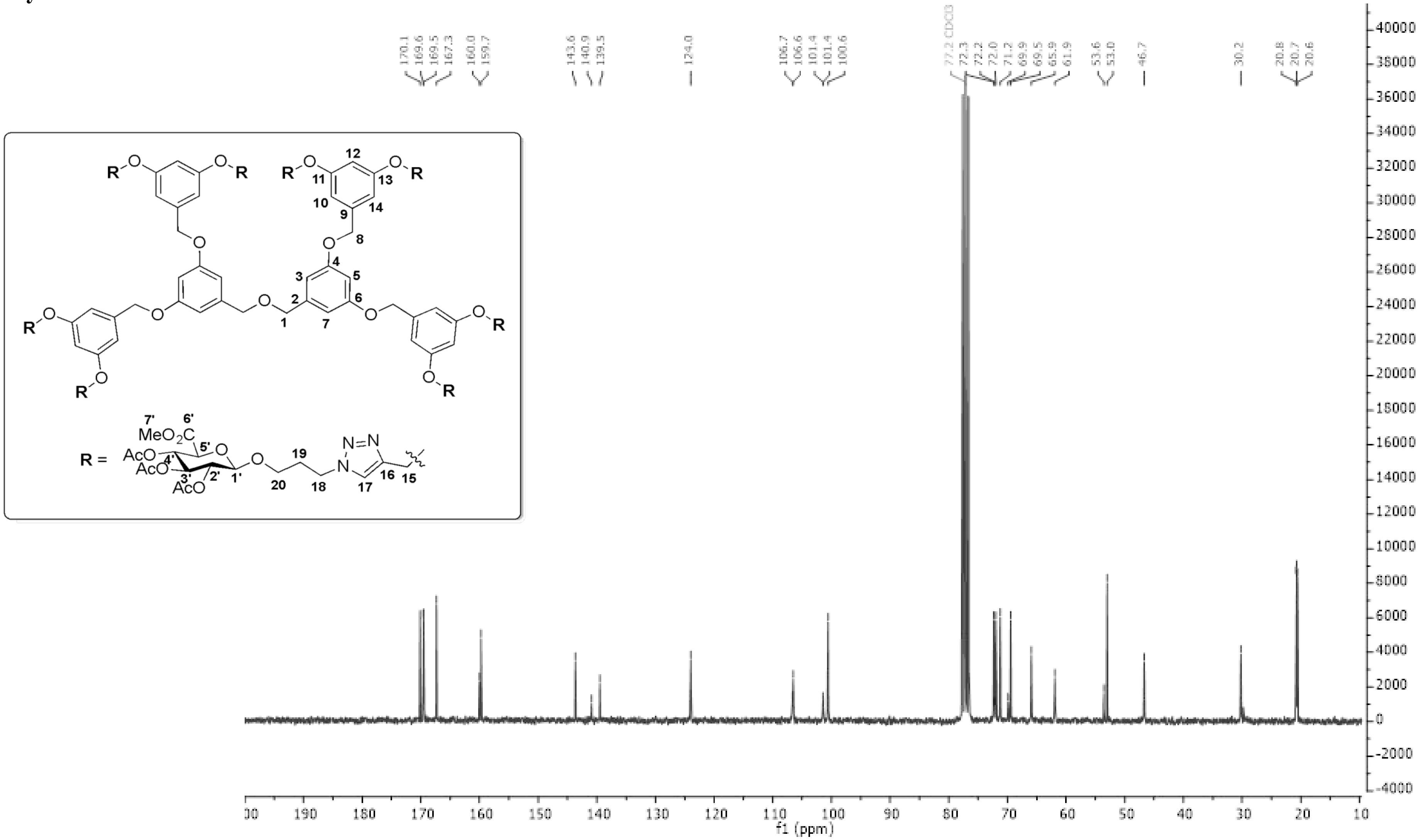

Glycodendrimer 11a

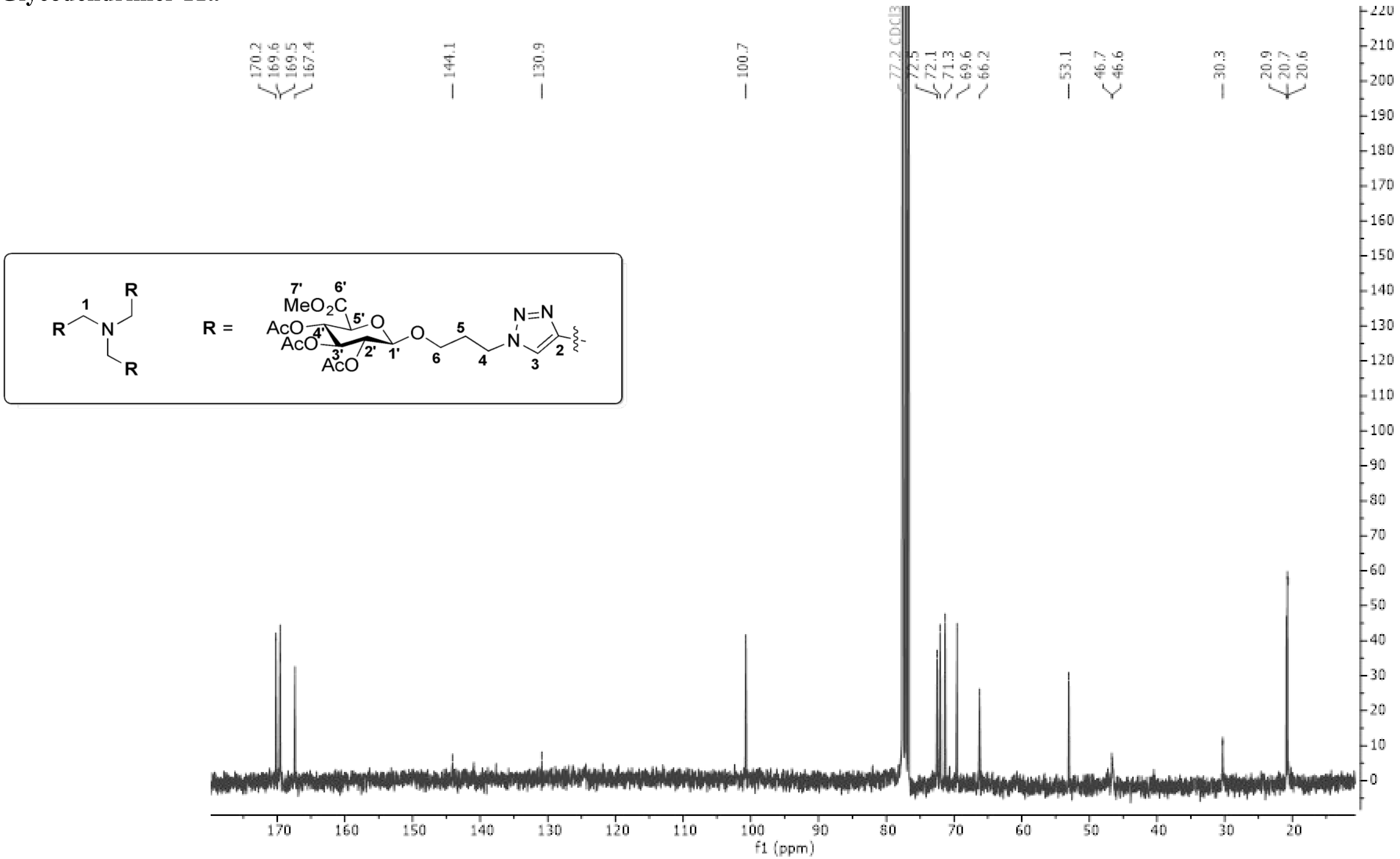

Glycodendrimer 11b

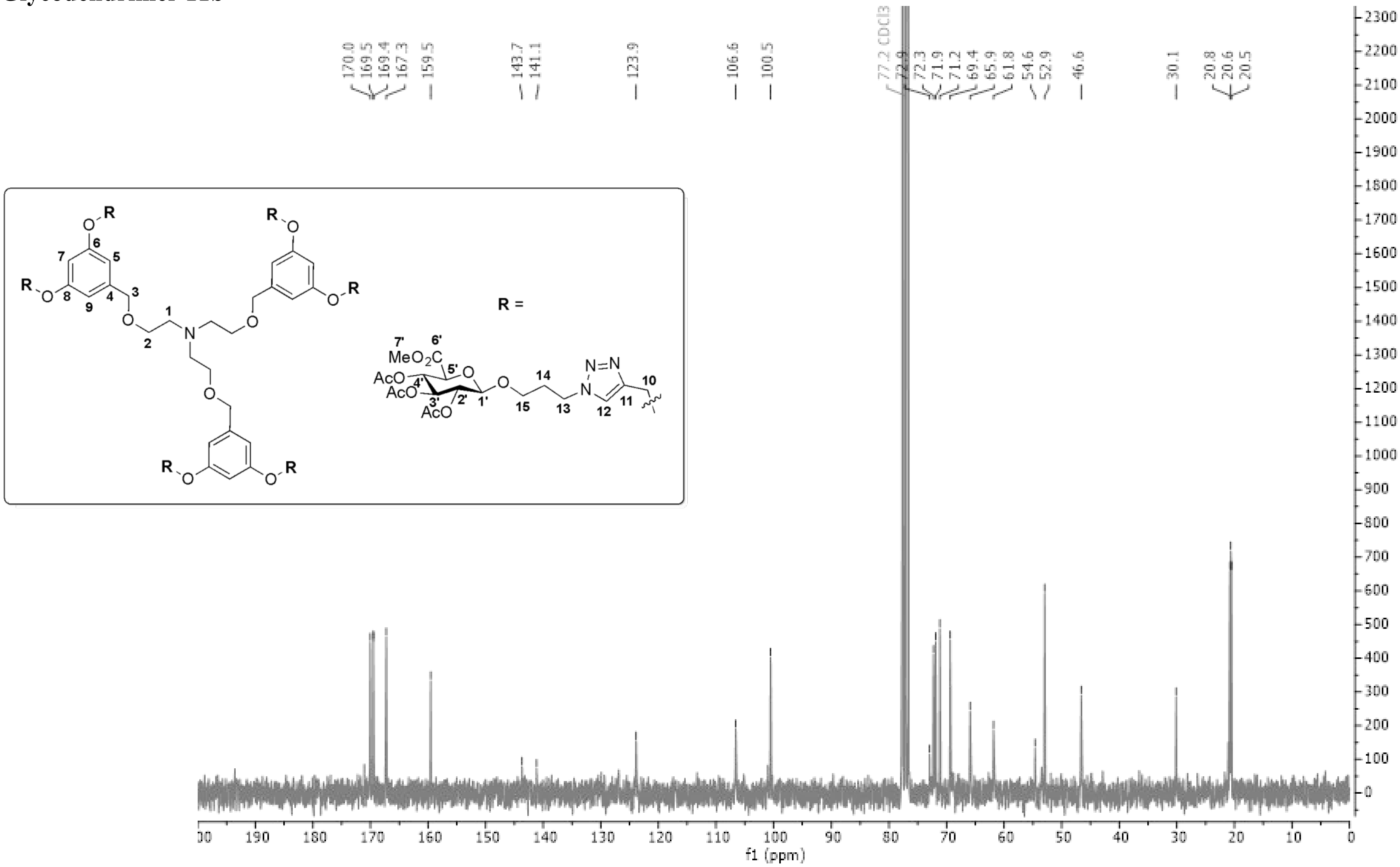

Glycodendrimer 11c

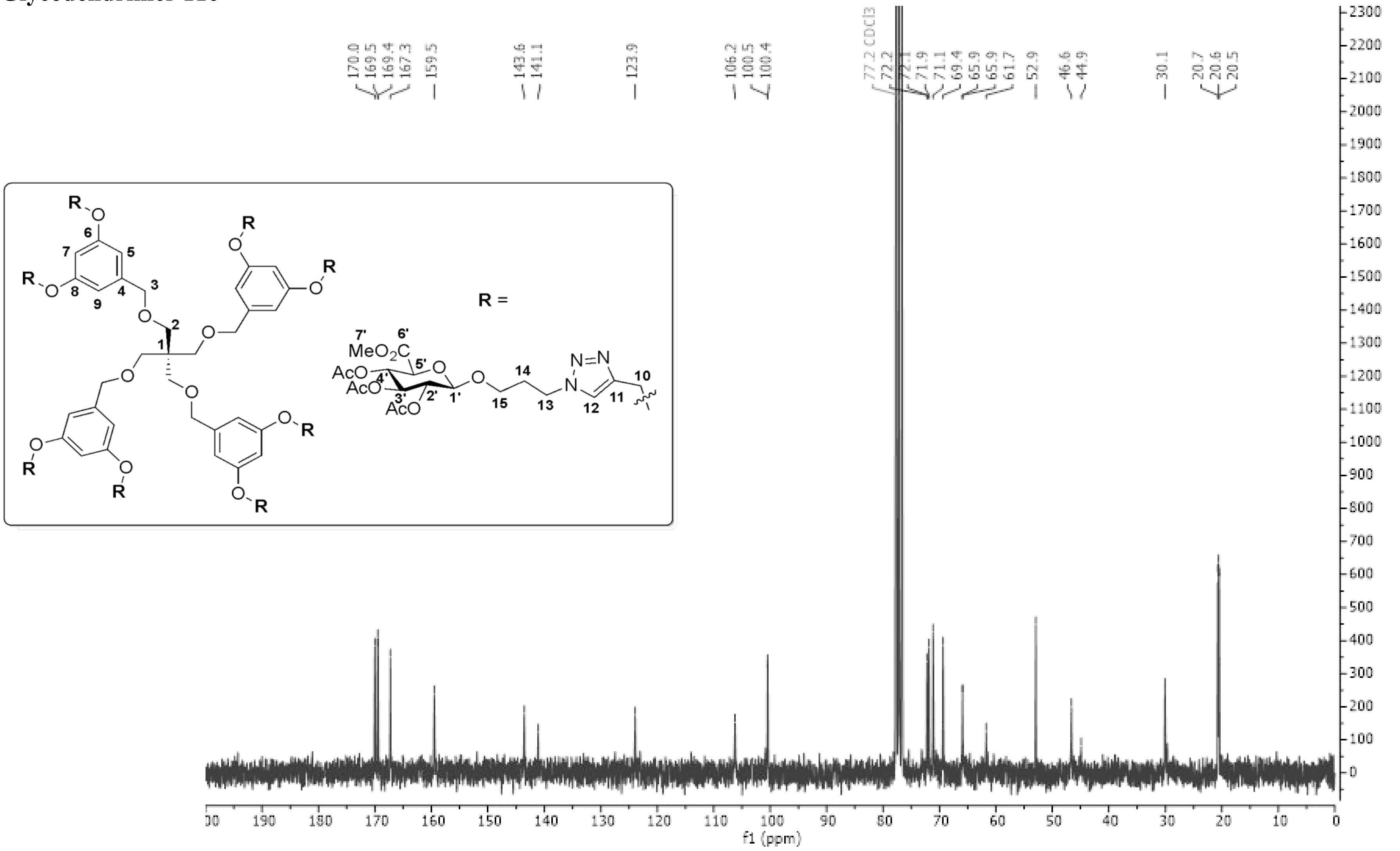

Glycodendrimer 4a

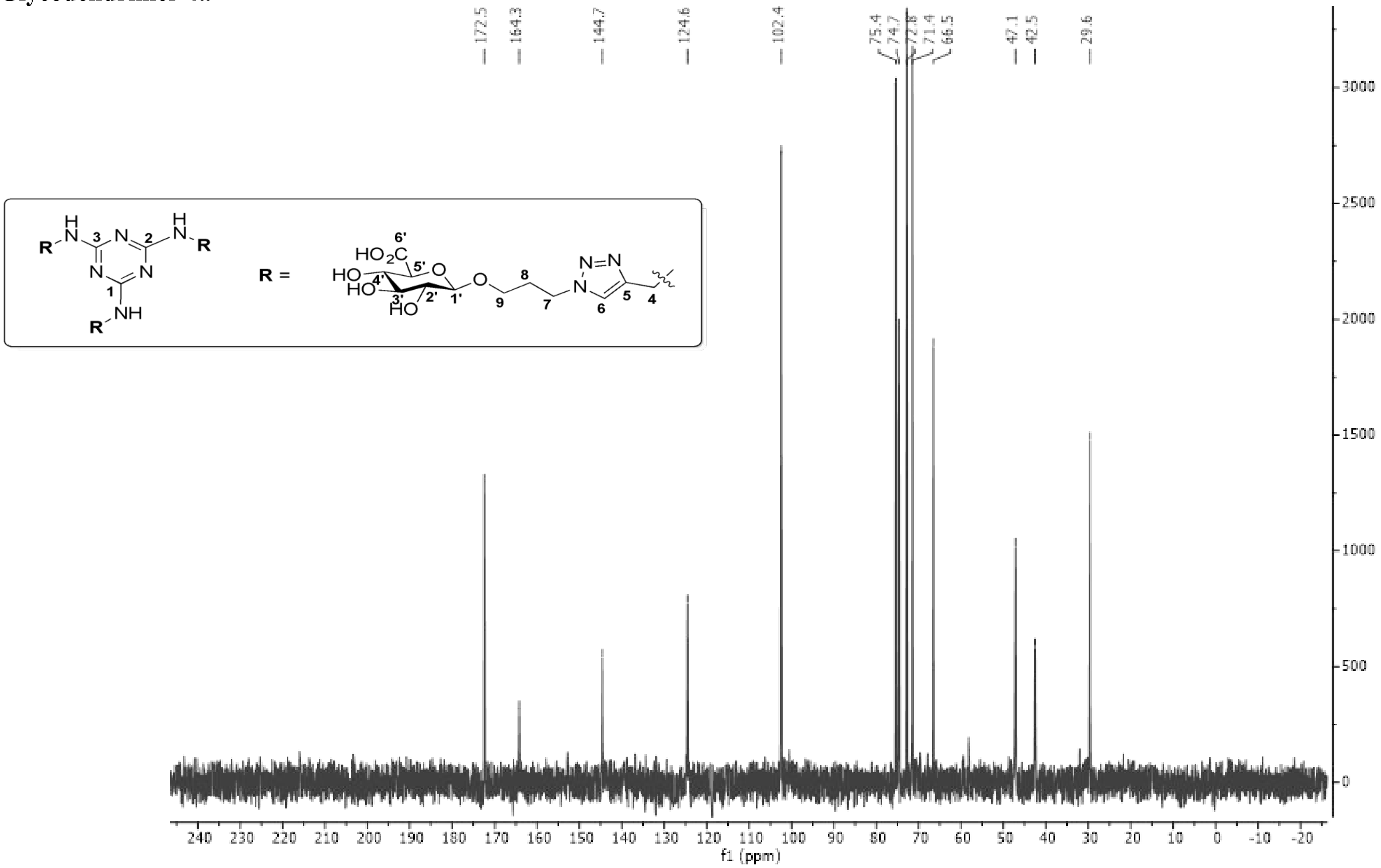

Glycodendrimer 4b

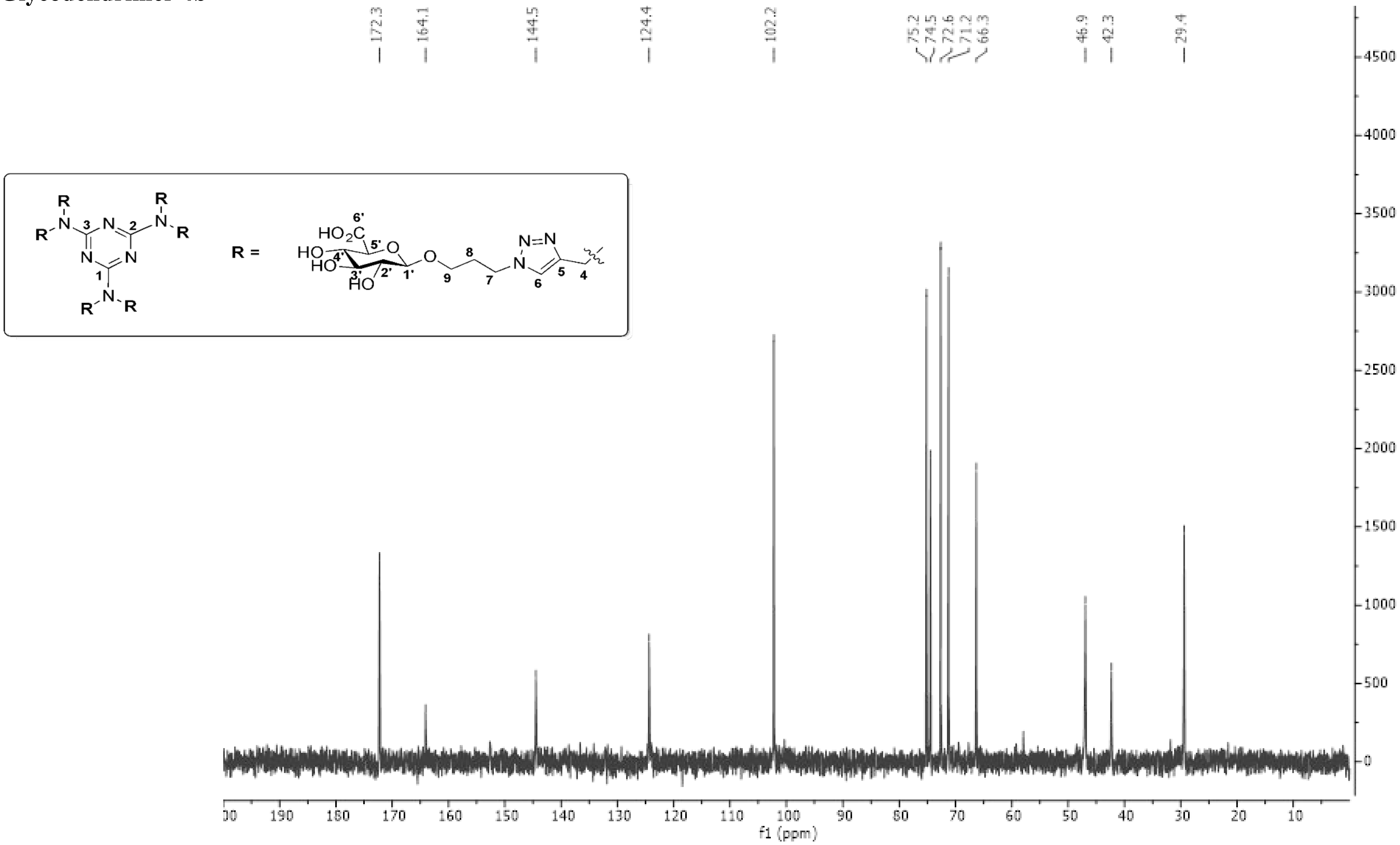

Glycodendrimer 4c

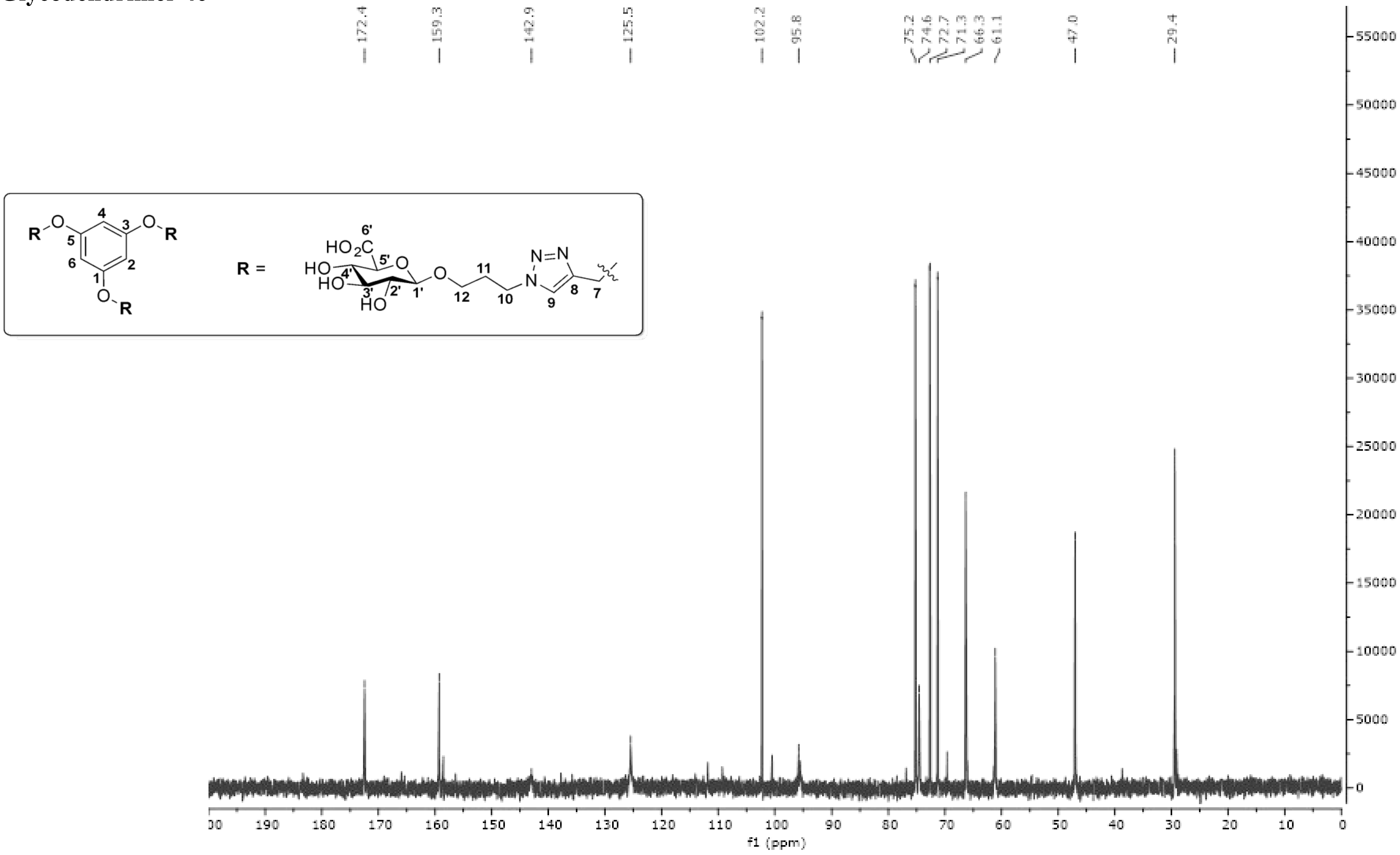

Glycodendrimer 4d

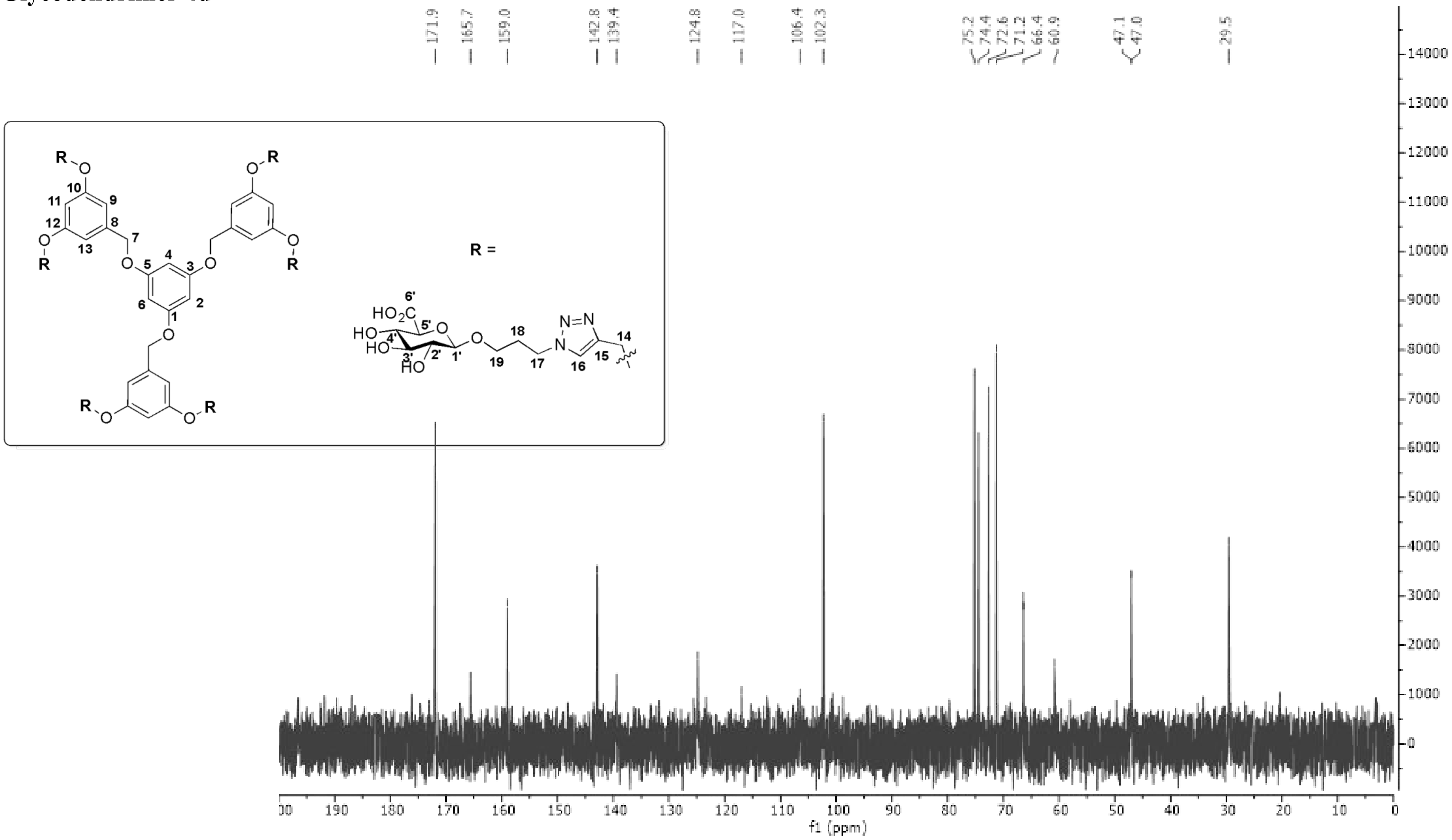

Glycodendrimer 4e

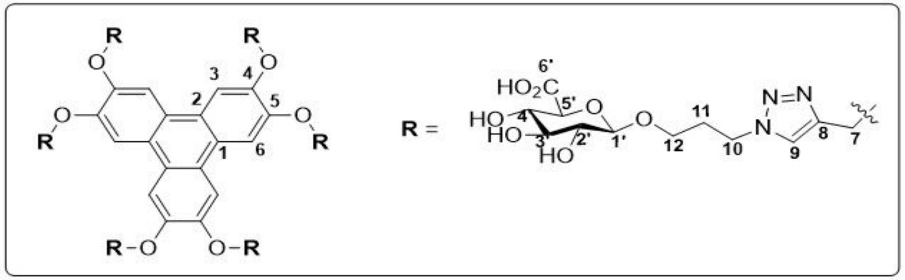

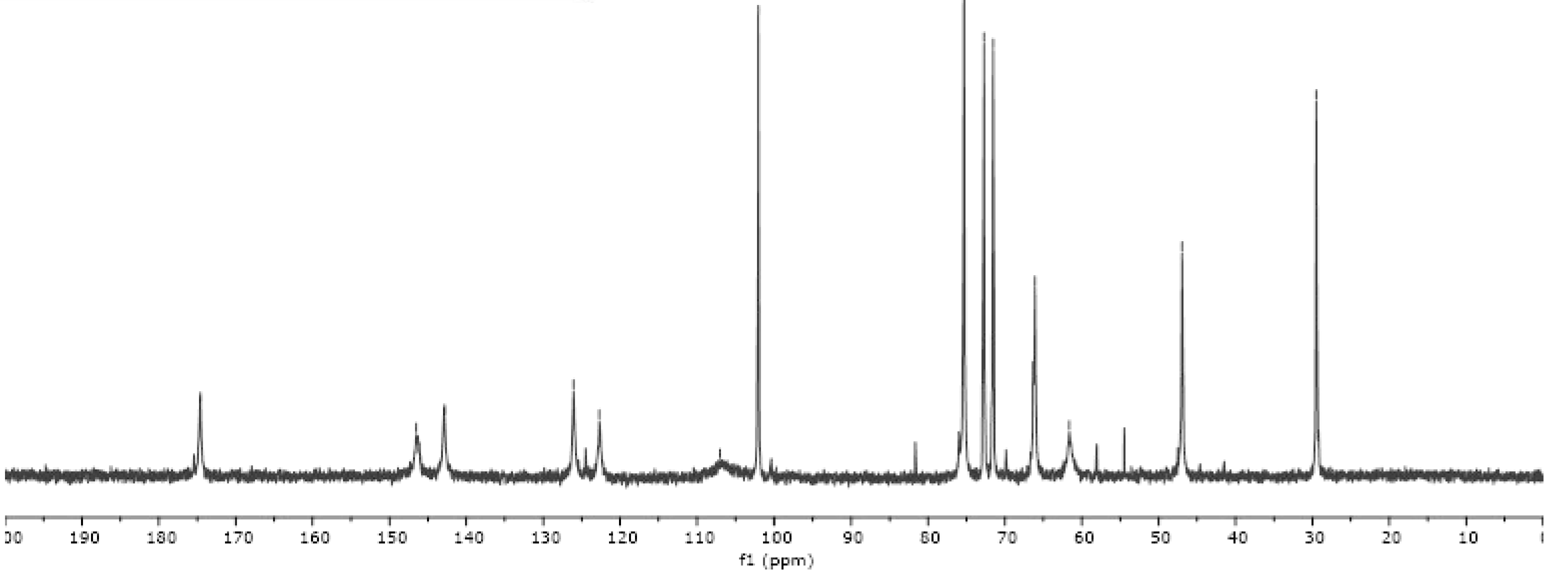

Glycodendrimer 4f

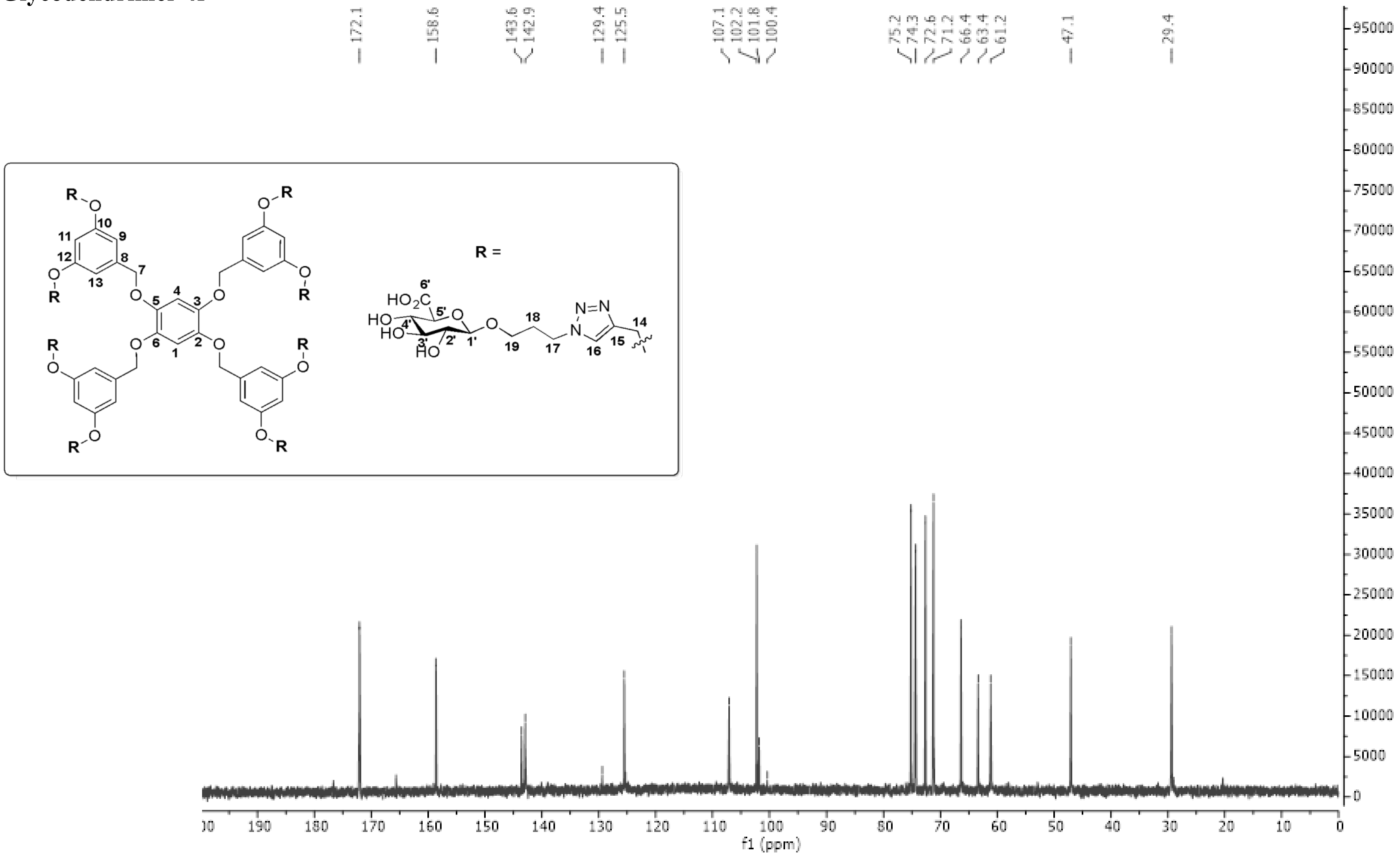

Glycodendrimer 9a

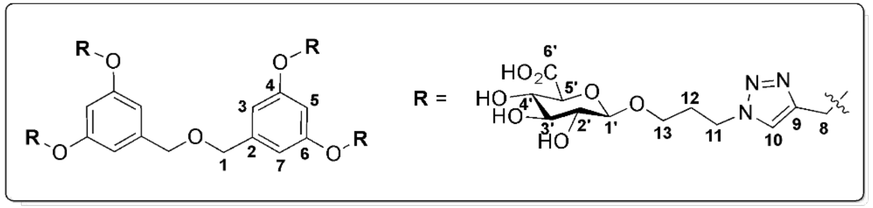

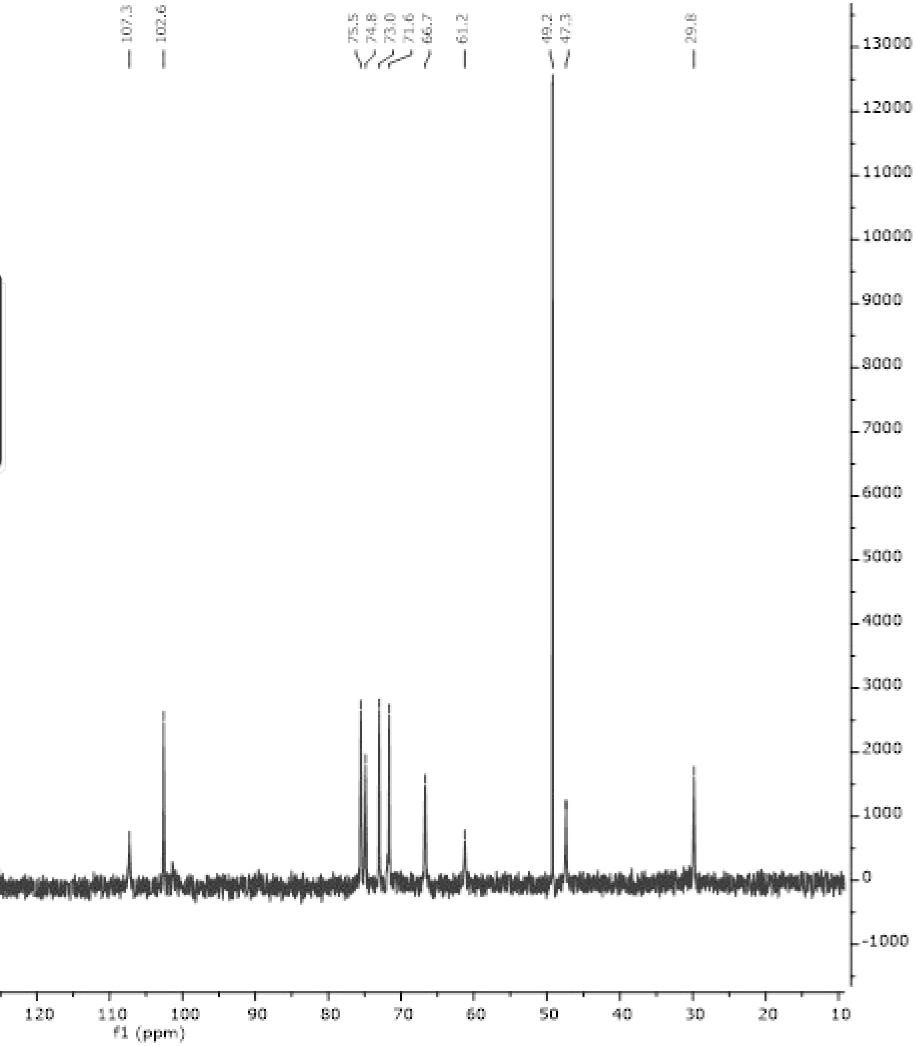

Glycodendrimer 9b

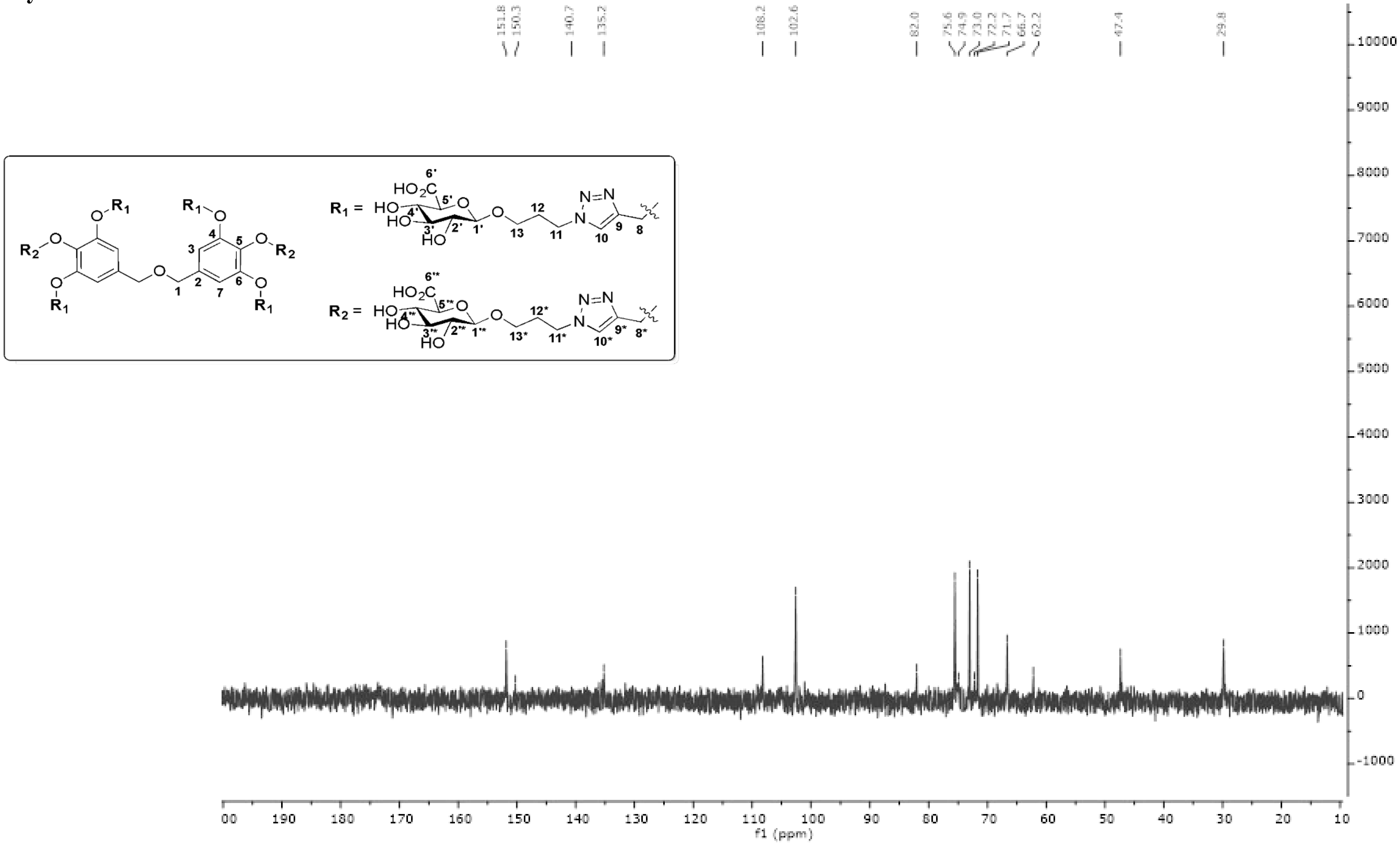

Glycodendrimer 9c

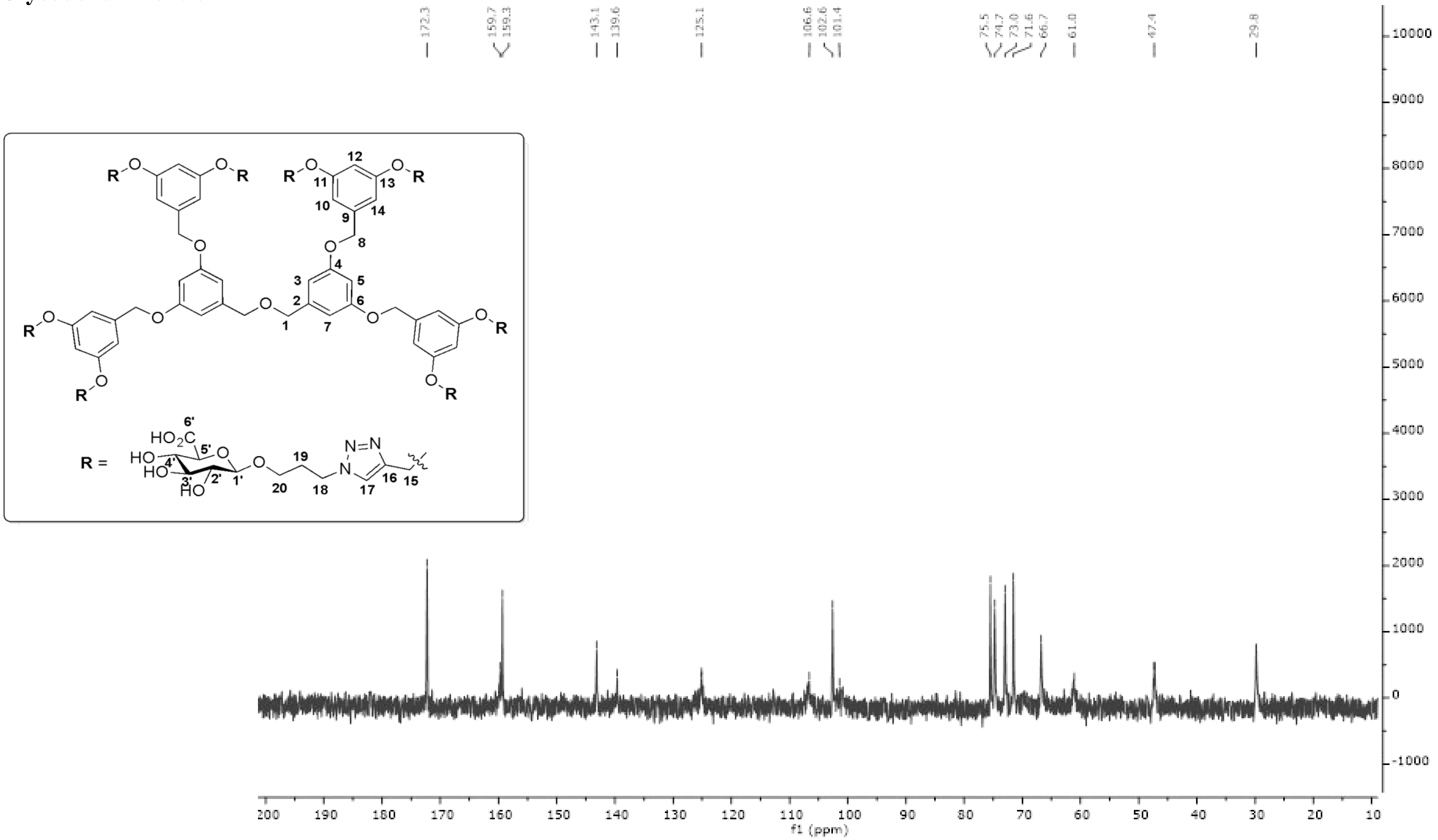

Glycodendrimer 12a

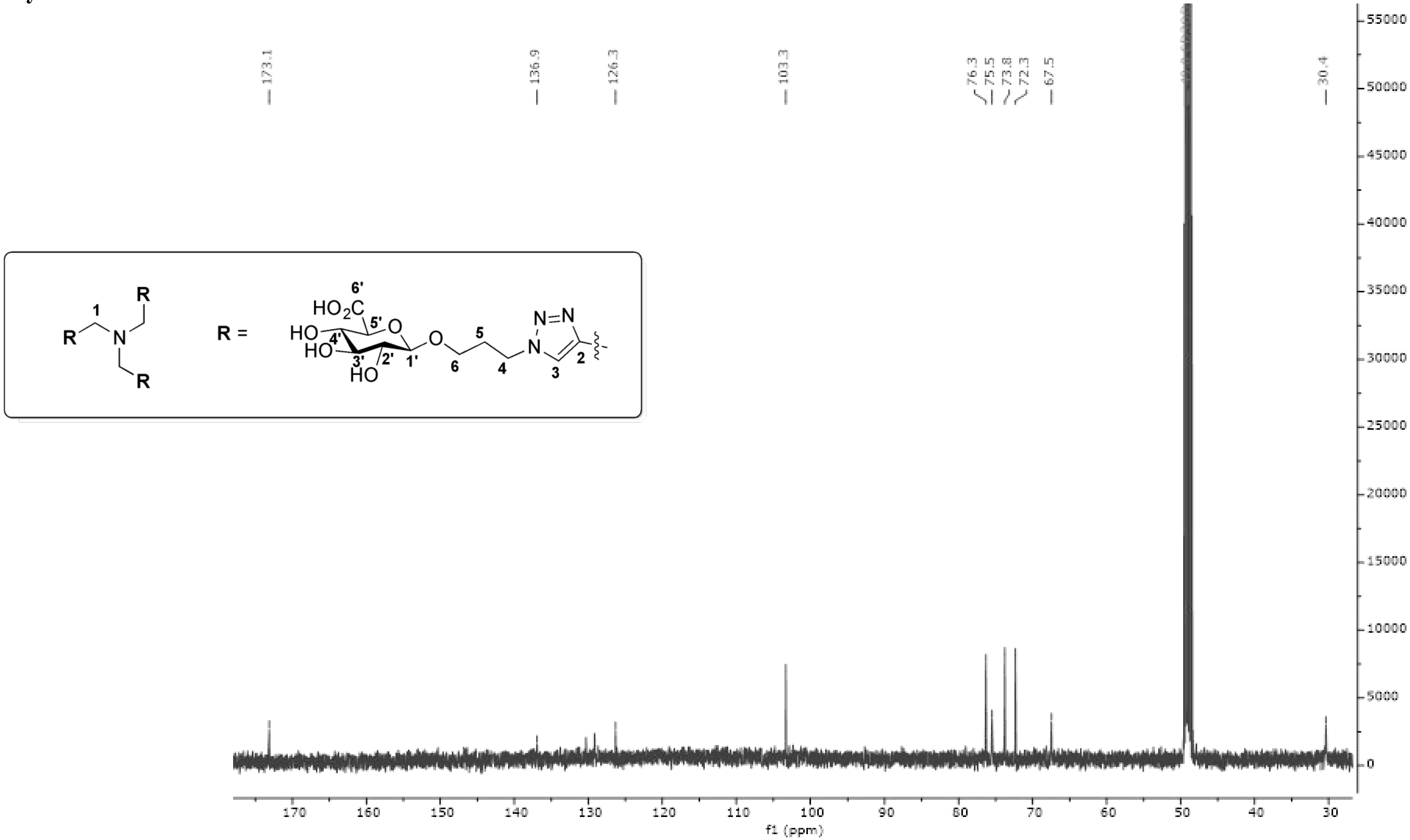

Glycodendrimer 12b

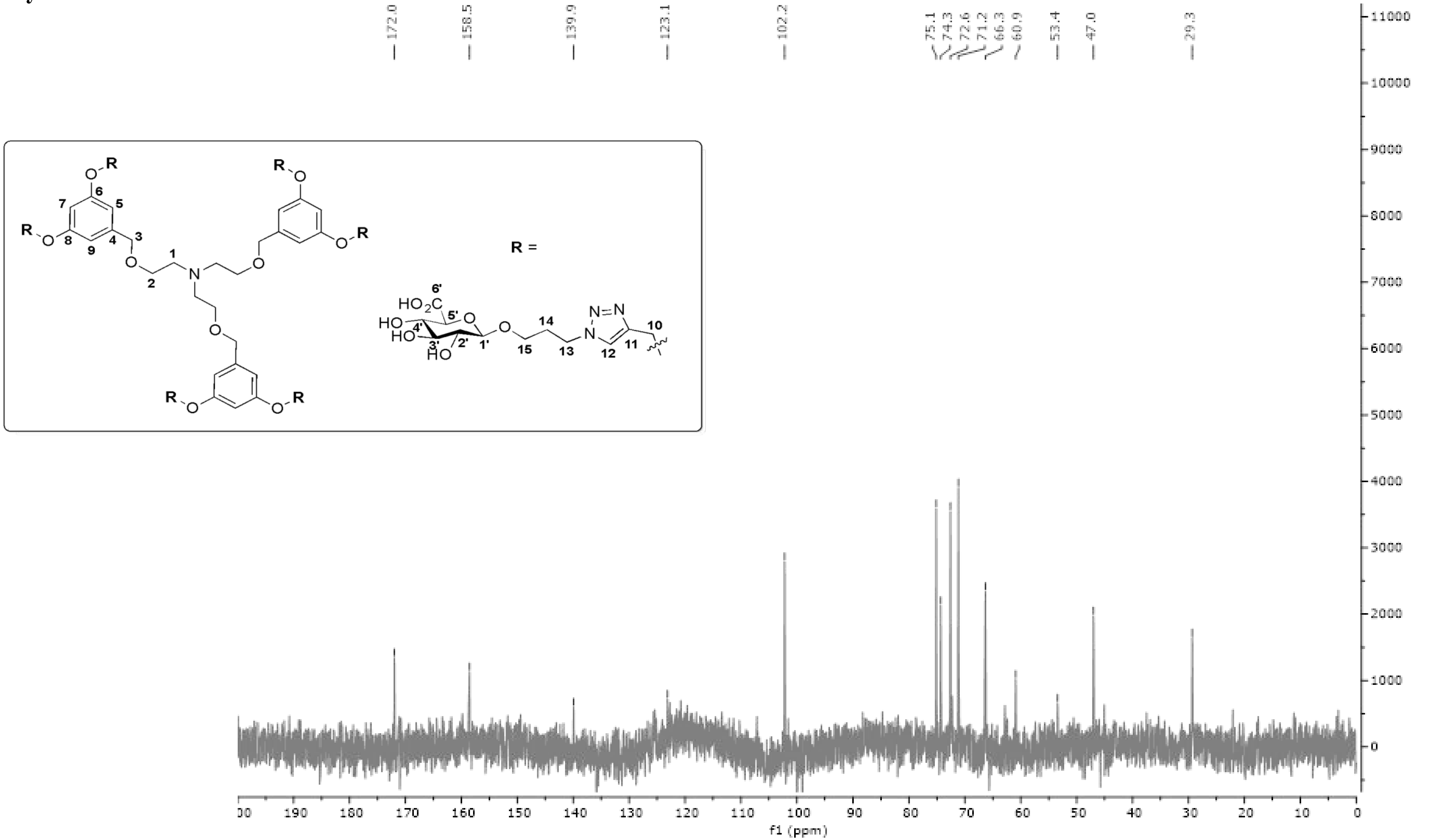

ASSOCIATED CONTENT

This material is available of charge on the ACS Publications website
